# Efficacy and Safety of the Neuroplastogen TSND-201 for the Treatment of PTSD: A Randomized Clinical Trial

**DOI:** 10.1001/jamapsychiatry.2025.4625

**Published:** 2026-02-18

**Authors:** Amanda Jones, Jennifer Warner-Schmidt, Hannah Kwak, Martin Stogniew, Blake Mandell, Terence H.W. Ching, Murray B. Stein, Benjamin Kelmendi

**Affiliations:** 1 Transcend Therapeutics, New York, New York; 2 Department of Psychiatry, Yale School of Medicine, New Haven, Connecticut; 3 Department of Psychiatry and School of Public Health, University of California San Diego, La Jolla

## Abstract

**Importance:**

The phase 2 data presented here support the development of TSND-201 for posttraumatic stress disorder (PTSD), a disorder for which there is a significant unmet need for rapid-acting and effective treatments. TSND-201 (methylone) is a highly selective, rapid-acting neuroplastogen that releases serotonin, norepinephrine, and dopamine without direct activity at 5-hydroxytryptamine (5-HT) 2A receptors that has shown rapid, robust, and long-lasting benefit for preclinical PTSD-related behaviors and has been well tolerated in phase 1 studies of healthy volunteers.

**Objective:**

To evaluate the efficacy and safety of TSND-201 vs placebo in adults with PTSD.

**Design, Setting, Participants:**

A Study to Assess the Use of Methylone in the Treatment of PTSD (IMPACT-1) part B was a phase 2, multicenter, double-blind, placebo-controlled, 10-week randomized clinical trial of TSND-201 in people with PTSD conducted between November 29, 2023, and February 19, 2025, across 16 sites in the US, UK, and Ireland. Adults aged 18 to 65 years who met *DSM-5* criteria for current PTSD and 6 months or more of symptoms (Clinician-Administered PTSD Scales for *DSM-5* [CAPS-5] ≥35) were eligible.

**Interventions:**

Participants were randomized 1:1 to receive TSND-201 or placebo. There were 4 once-weekly oral dosing sessions (150 mg followed by 100 mg or placebo). No psychotherapy was provided; however, dosing sessions were monitored by mental health professionals using a nondirective approach. Participants were followed up for 6 weeks after the last dose.

**Main Outcomes and Measures:**

The primary end point was change from baseline to day 64 in the CAPS-5 total severity score. Secondary end points included changes in PTSD Checklist for *DSM-5* (PCL-5), Sheehan Disability Scale (SDS), and Montgomery-Åsberg Depression Rating Scale (MADRS) scores. Other measures included response (≥50% improvement from baseline), remission (≤11 total severity score), loss of PTSD diagnosis, changes in CAPS-5 symptom clusters, and incidence of treatment-emergent adverse events (TEAEs). Safety was assessed by monitoring adverse events, vital signs, and Columbia-Suicide Severity Rating Scale.

**Results:**

Among the 65 participants (mean [SD] age, 43.7 [10.5] years; 39 female [60.0%]), TSND-201 demonstrated significantly greater improvement in CAPS-5 total score than placebo (least-squares mean difference, 9.64; 90% CI, −16.48 to −2.80; *P* = .01). PCL-5 (−28.46 vs −19.47; LS mean treatment difference, −8.99; 90% CI, −17.81 to −0.17), SDS (−8.29 vs −3.57; LS mean treatment difference, −4.72; 90% CI, −8.84 to −0.61), and MADRS (−13.94 vs −7.73; LS mean treatment difference, −6.21; 90% CI, −12.41 to −0.27) scores were also improved. Common TEAEs in the TSND-201 group included headache, decreased appetite, nausea, dizziness, blood pressure increased, dry mouth, insomnia.

**Conclusions and Relevance:**

Results of this randomized clinical trial reveal that TSND-201 demonstrated statistically significant efficacy and was well tolerated, supporting its potential as a rapid-acting, durable treatment for PTSD.

**Trial Registration:**

ClinicalTrials.gov Identifier: NCT05741710

## Key Points

**Question:**

Is the neuroplastogen TSND-201 (methylone) efficacious and well tolerated in people with posttraumatic stress disorder (PTSD)?

**Findings:**

In this phase 2, double-blind, placebo-controlled randomized clinical trial in 65 people with severe PTSD, acute intermittent treatment with TSND-201 was associated with a statistically significant and clinically meaningful reduction in PTSD symptoms, measured by Clinician-Administered PTSD Scales for *DSM-5* scores, compared with placebo. TSND-201 was generally safe and well tolerated; adverse events were typically transient, occurring on the day of dosing and resolving within a day.

**Meaning:**

Study results demonstrate that TSND-201 has rapid, robust, and durable efficacy and is well tolerated in people with PTSD, supporting its further development as a treatment for PTSD.

## Teaser

This randomized clinical trial investigates if TSND-201 (methylone) is efficacious and safe in people with posttraumatic stress disorder (PTSD).

## Introduction

There is an urgent unmet need for efficacious, rapid-acting treatments for posttraumatic stress disorder (PTSD). PTSD is a common, costly, often severe and debilitating disorder characterized by symptoms of intrusive memories, avoidance behaviors, negative changes in thinking and mood, and heightened arousal or reactivity.^1,2^ Currently, only 2 pharmacological agents, paroxetine and sertraline—both selective-serotonin reuptake inhibitors—have been US Food and Drug Administration approved to treat PTSD. Both have limited effectiveness, delayed onset of efficacy, and the potential for significant, chronic adverse effects.^3,4^ Newer compounds with acute and intermittent dosing paradigms may be clinically useful as they have the potential to produce rapid efficacy with limited and/or transient adverse effects.

TSND-201 is a highly selective, rapid-acting neuroplastogen in clinical development for PTSD. TSND-201 is the β-ketone analog of 3-4 methylenedioxymethamphetamine (MDMA), which has shown benefit for PTSD in clinical trials in combination with psychotherapy.^5,6^ Despite its structural similarity, TSND-201 shows distinct pharmacological and subjective effects compared with MDMA,^7,8,9,10^ due at least in part to greater selectivity for the serotonin, norepinephrine, and dopamine transporters.^10,11,12,13,14,15^

TSND-201 increases the release of serotonin, norepinephrine, and dopamine, leading to rapid and long-lasting effects on neuroplasticity in brain areas affected by PTSD, including increased neurotrophic support as well as direct effects on neurite outgrowth.^10,16,17^ It has no direct agonist or antagonist activity at the serotonin 2A (5-hydroxytryptamine [5-HT] 2A) receptor, consistent with no hallucinogenic activity in humans^7,8,9,18^ or head-twitch responses in animals.^19^ It does not deplete brain monoamines (ie, serotonin, dopamine), due at least in part to its lack of activity at the vesicular monoamine transporter 2 (VMAT2)^11,12,13^ and is highly selective with no agonist/antagonist activity observed at 168 G-protein coupled receptors.^10^ In preclinical paradigms, TSND-201 has shown fast-acting, robust, long-lasting anxiolytic and antidepressantlike activity^20,21^ and improved fear extinction learning and recall,^16,22^ which is thought to be disrupted in PTSD.^23^ It has also been well tolerated in clinical studies of healthy volunteers.^7,8,9^ Together, these results support the rationale for developing TSND-201 as a fast-acting, durable treatment for PTSD. Here, we report results from a phase 2 study to evaluate the use of methylone for the treatment of PTSD.

## Methods

A Study to Assess the Use of Methylone in the Treatment of PTSD (IMPACT-1) trial was a 2-part multicenter study to assess TSND-201 for the management of symptoms of PTSD. The trial protocol and statistical analysis plan are available in Supplement 1 and Supplement 2, respectively. Part A was an open-label, noncontrolled assessment of TSND-201 to assess the safety and feasibility of TSND-201 before initiation of the placebo-controlled portion (part B). Part A results have been previously reported^18^ (eTable 1 and 2 and eFigure 1 and 2 in Supplement 3) and are included in the eAppendix in Supplement 3. Here, we present the results of part B, a double-blind, placebo-controlled randomized clinical study conducted between November 29, 2023, and February 19, 2025, at 16 sites across the US, UK, and Ireland. The trial was conducted in accordance with the International Council of Harmonisation guidelines for Good Clinical Practice, and relevant regulations in the countries where the research was conducted. The trial protocol and written informed consent were reviewed and approved by relevant ethics committee or institutional review board for each country. Results are consistent with the Consolidated Standards of Reporting Trials (CONSORT) reporting guidelines.

### Participants and Randomization

Eligible participants were adults aged 18 to 65 years who met the *DSM-5* criteria for current PTSD with at least 6 months of symptoms at screening assessed by the Clinician-Administered PTSD Scales for *DSM-5* (CAPS-5).^24^ A CAPS-5 total score of 35 or greater at screening and 28 or greater at baseline was required. All participants must have tried at least 1 treatment for PTSD (eg, pharmacotherapy or psychotherapy). Participants self-reported race and ethnicity information, which included African American or Black, Asian, White, and other (ie, race not reported). Participant race and ethnicity information was collected per US Food and Drug Administration requirement. Key exclusion criteria were a primary diagnosis of any other *DSM-5* disorder; concurrent use of antidepressants or other PTSD treatments including psychotherapy, antipsychotics, lithium, or other mood stabilizers; moderate or severe substance/alcohol use disorder (moderate alcohol use disorder in early remission was allowed); active suicidal ideation within 2 months of screening or any history of suicidal behavior within the last 5 years assessed by the Columbia-Suicide Severity Rating Scale (C-SSRS)^25^; and use of a psychedelic within 12 months of screening. The current study had broad PTSD criteria, including complex PTSD without restrictions on age of trauma, duration since trauma, or trauma type (combat trauma was allowed). Full eligibility criteria are provided in the protocol in Supplement 1.

Eligible participants were randomized to receive either TSND-201 or placebo using an interactive web response system. The 1:1 randomization used a 4-block size without stratification by any covariates. TSND-201 (or matched placebo) was dosed orally as an initial administration of 150 mg (3 capsules), followed by a booster administration of 100 mg (2 capsules) 90 minutes later. TSND-201 and placebo capsules were identical. Because effects of methylone on blood pressure, for example, appear to track closely with its peak plasma concentrations (Cmax),^7,8^ a split or booster dose strategy was used to optimize efficacy while reducing Cmax and potential adverse effects. Doses were administered on days 1, 8, 15, and 22. Participants fasted for 2 hours before the initial dose and 2 hours after the booster dose. After treatment, participants entered a 6-week follow-up period. All participants, trial site personnel, central raters conducting efficacy assessments, and the sponsor were blinded to treatment assignment.

### Procedures

Following a screening period up to 28 days, eligible participants met with their dosing session monitor during a predosing visit to establish rapport and clarify any questions they may have about the dosing sessions. Each dosing session lasted at least 8 hours or until all study drug effects subsided. Participants attended a total of 4 dosing sessions, each separated by 1 week. During the dosing session, dosing session monitors monitored participants via a nondirective approach (ie, attending silently, letting participants talk or responding with simple utterances or minimal open-ended questions, rather than engaging in directive, structured psychotherapy). Dosing session monitors had graduate-level professional training, with clinical experience in psychotherapy, and were licensed to practice independently. The sponsor oversaw the monitors, including reviewing session videos (with participant consent) to ensure adherence to the nondirective approach. Monitors received regular supervision from trainers to support compliance with this approach. Efficacy assessments occurred 2 days after each dosing session. After the treatment period, follow-up efficacy and safety visits were conducted on days 29, 36, 43, 57, and 64 (end of study).

### Outcomes

The primary end point was the mean change in CAPS-5 total score from baseline to day 64 (week 10) compared with placebo. The CAPS-5 is a validated structured interview used to assess PTSD diagnostic status and track symptom severity over time.^24^ The total score is calculated by summing severity scores for 20 *DSM-5* PTSD-symptom items that are scored from 0 (absent) to 4 (extreme), whereas symptom cluster scores are calculated by summing individual-item severity scores for symptoms in a given cluster (criterion B [intrusion]: items 1-5; criterion C [avoidance]: items 6-7; criterion D [negative alterations in cognitions and mood]: items 8-14; criterion E [arousal and reactivity]: items 15-20). The CAPS-5 total score ranges from 0 to 80 points, with higher scores indicating greater PTSD symptom severity. The past-month version of the CAPS-5 was completed at screening, and the past-week version was used at all other visits.

Additional end points related to the CAPS-5 were the percentage of participants with a treatment response (≥50% improvement from baseline on CAPS-5 total severity score), remission (a score of ≤11 on the CAPS-5 total severity score),^26,27^ and loss of PTSD diagnosis.

Secondary end points included mean changes in PTSD Checklist for *DSM-5* (PCL-5) score, Sheehan Disability Scale (SDS) mean domain score, and Montgomery-Åsberg Depression Rating Scale (MADRS) score from baseline to end of study.

### Safety Assessments

Adverse events (AEs) were collected from randomization until the end of study. Treatment-emergent AEs (TEAEs) were defined as any AE that occurred after the first administration of study drug and not more than 7 days after the last dose unless suspected to be related to study drug. Safety assessments included TEAEs, vital signs, electrocardiogram, and the C-SSRS to monitor suicidal ideation and behavior.

### Blinding Assessment

After completion of all 4 treatment visits (day 28), participants were asked to guess which treatment they received. Then, they selected a number from 0 to 10 corresponding to their confidence in that choice (0 = strong belief they received placebo; 10 = strong belief they received active drug). Scores of 0 to 3 were categorized as “likely received placebo,” scores of 4 to 7 were categorized as “unsure which treatment received,” and scores of 8 to 10 were categorized as “likely received active treatment.”

### Statistical Analysis

A sample size of 64 participants was estimated to provide approximately 90% power to detect a between-treatment effect of approximately 11 points on the CAPS-5 with a pooled standard deviation of 12 points, anticipating an approximate 15% dropout rate, at a 2-sided significance test of .05. These assumptions were based on the phase 3 trial of MDMA for PTSD.^5^

All efficacy analyses were performed using the modified intention-to-treat (mITT) population defined as all randomized participants who received at least 1 dose of study drug and had at least 1 valid postbaseline assessment. The primary efficacy end point, past-week CAPS-5 total severity score change from baseline to day 64 compared with placebo, was estimated using a mixed model for repeated measures (MMRM) with SAS software, version 9.4 (SAS Institute). There was no additional statistical imputation for missing data. The MMRM model included treatment, visit, and treatment by visit interaction as fixed effects with visit as the repeated effect. The end points baseline value and sex were covariates. Least-squares (LS) mean change from baseline, SE, and the LS mean difference between TSND-201 and placebo at end of study and 90% CI were calculated. For the additional end point analyses, the percentage of participants meeting the predefined criteria was calculated and summarized by group. The between-group comparisons were calculated using the Cochran-Mantel-Haenszel test. All statistical tests were based on a 1-sided significance level of .05, and therefore, 2-sided 90% CI were calculated, as specified in the statistical analysis plan (Supplement 2), and finalized before unblinding of the study data.

Safety assessments were analyzed using the safety population, which included all participants who received at least 1 administration of study drug. The number of participants experiencing TEAEs and SAEs was summarized by treatment group.

## Results

### Participants

**Figure 1.**
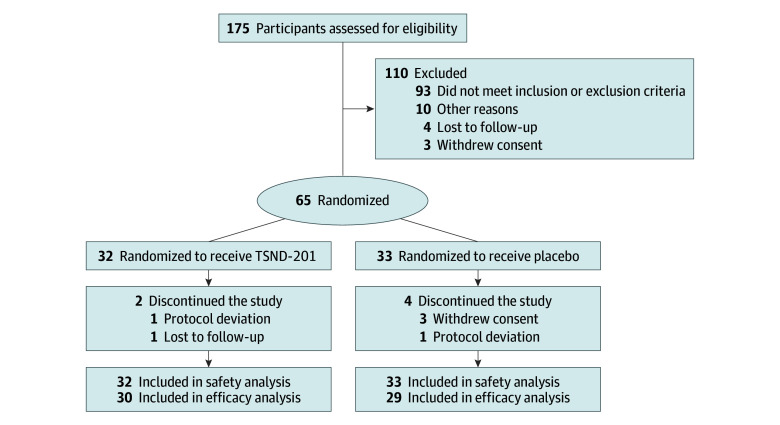
Consolidated Standards of Reporting Trials (CONSORT) Flow Diagram for the IMPACT-1 Trial (All Population) A, The modified intent-to-treat population, used for efficacy analysis, included all participants who were randomized, received at least 1 dose of study drug, and have at least 1 valid postbaseline assessment. B, The safety population included all participants who received at least 1 administration of study treatment. IMPACT-1 indicates A Study to Assess the Use of Methylone in the Treatment of PTSD; PTSD, posttraumatic stress disorder.

A total of 175 participants were screened, and 65 (mean [SD] age, 43.7 [10.5] years; 39 female [60.0%]; 26 male [40.0%]) were randomized to receive TSND-201 (n = 32) or placebo (n = 33) (Figure 1). Participants self-reported the following races and ethnicities: 2 African American or Black (3.1%), 2 Asian (3.1%), 58 White (89.2%), and 3 other race and ethnicity (4.6%). Dosing compliance remained high (>80%) in both groups for all dosing sessions. The safety population included all 65 participants, and the mITT population included 60 participants (TSND-201, n = 30; placebo, n = 30). Six participants from the safety population terminated from the trial early (TSND-201, n = 2; placebo, n = 4), with the most common reasons being withdrawal of consent followed by protocol deviation and loss to follow-up.

Baseline demographics and characteristics were similar between groups (Table 1). The mean (SD) baseline CAPS-5 total scores were 45.8 (7.11) and 46.0 (5.42) in the TSND-201 and placebo groups, respectively.

**Table 1.** Baseline Demographics and Characteristics (Safety Population)

Characteristic	TSND-201 (n = 32)	Placebo (n = 33)	Overall (N = 65)
Age, mean (SD), y	45.1 (10.60)	42.2 (10.34)	43.7 (10.49)
Sex, No. (%)			
Female	19 (59.4)	20 (60.6)	39 (60.0)
Male	13 (40.6)	13 (39.4)	26 (40.0)
Race, No. (%)			
African American or Black	0	2 (6.1)	2 (3.1)
Asian	1 (3.1)	1 (3.0)	2 (3.1)
White	29 (90.6)	29 (87.9)	58 (89.2)
Other^a^	2 (6.3)	1 (3.0)	3 (4.6)
Body mass index, mean (SD)^b^	26.83 (5.331)	27.05 (5.212)	26.94 (5.229)
Duration of PTSD symptoms, mean (SD), y	19.68 (14.332)	18.41 (13.187)	19.04 (13.669)
Age at end date of index trauma, mean (SD), y	23.59 (11.042)	23.33 (10.304)	23.46 (10.590)
Prior PTSD treatments, No. (%)			
Pharmacotherapy	23 (71.9)	20 (60.6)	43 (66.2)
Psychotherapy	24 (75.0)	26 (78.8)	50 (76.9)
Baseline CAPS-5 total score, mean (SD)^c^	45.8 (7.11)	46.0 (5.42)	45.9 (6.27)
Trauma type, No. (%)			
Sexual trauma	17 (53.1)	12 (36.4)	29 (44.6)
Military	4 (12.5)	1 (3.0)	5 (7.7)
Other^d^	11 (34.4)	20 (60.6)	31 (47.7)

Abbreviations: CAPS-5, Clinician-Administered PTSD Scales for *DSM-5*; PTSD, posttraumatic stress disorder.

^a^ Other includes not reported race.

^b^ Calculated as weight in kilograms divided by height in meters squared.

^c^ Reported CAPS-5 baseline scores for modified intent-to-treat population.

^d^ Other includes traffic accident, abuse, exposure to a violent event or crime, near-death experience, or death of a relative.

### Primary Efficacy End Point

**Figure 2.**
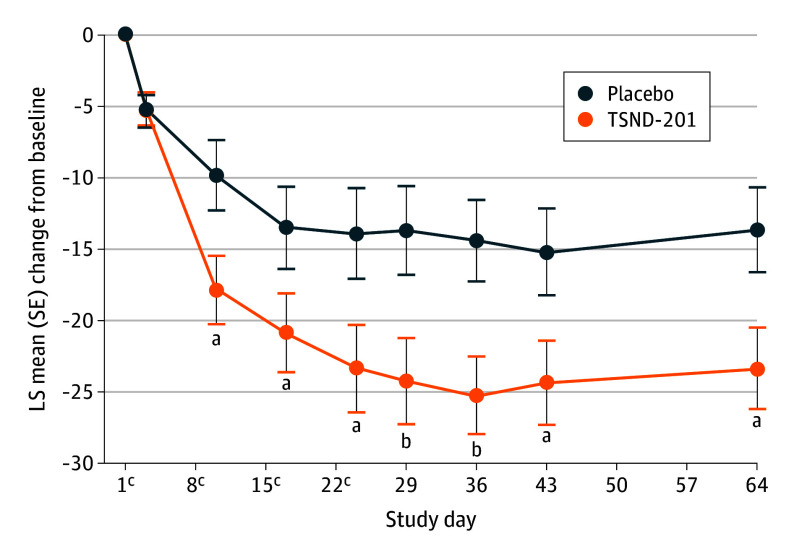
Line Graph Depicting the Clinician-Administered Posttraumatic Stress Disorder (PTSD) Scale for the *DSM-5* (CAPS-5) Over Time All efficacy analyses were performed using the modified intent-to-treat population, defined as all randomized participants who received at least 1 dose of study drug and had at least 1 valid postbaseline assessment. LS indicates least squares. ^a^*P* < .05. ^b^*P* < .01. ^c^Denotes dosing days 1, 8, 15, and 22. Assessments took place on 2 days after dosing (days 3, 10, 17, and 24) and during follow-up (days 29, 36, 43, and 64).

TSND-201 demonstrated significantly greater improvement from baseline to day 64 in CAPS-5 total score (LS mean [SE] change, −23.28 [2.84]) compared with placebo (LS mean [SE] change, −13.64 [2.95]), with an LS mean difference of −9.64 (90% CI, −16.48 to −2.80; *P* = .01). Greater improvement was demonstrated in the TSND-201 group from day 10 onward, with an LS mean (SE) change of 17.83 (2.38) for TSND-201 compared with 9.83 (2.49) for placebo (LS mean difference, −8.00; 90% CI, 13.75-2.26; *P* = .01) (Figure 2).

### Additional End Points

**Figure 3.**
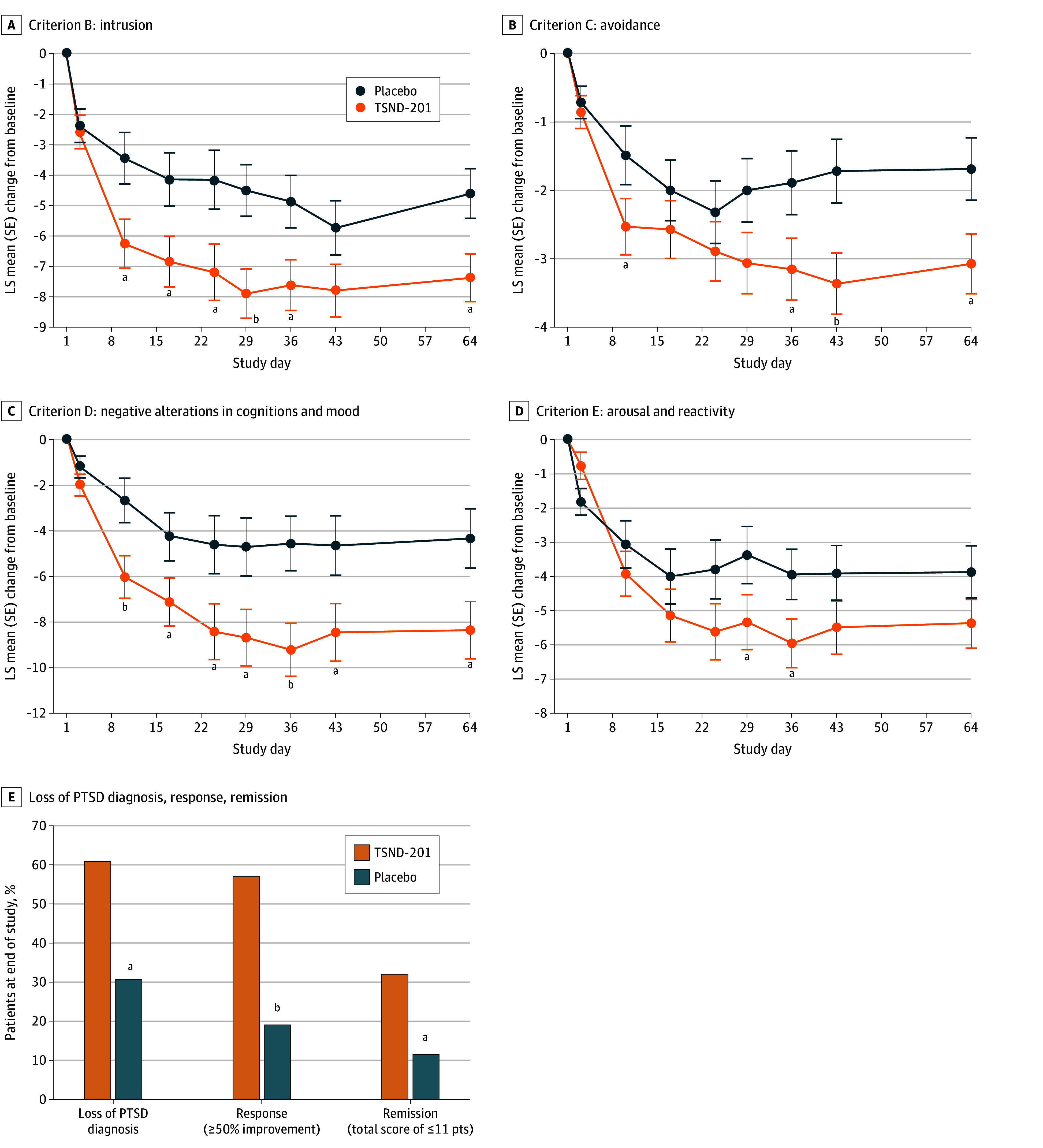
Line Graphs and Bar Chart of the Clinician-Administered PTSD Scale for the *DSM-5* (CAPS-5) Cluster Analysis and Loss of PTSD Diagnosis, Response, and Remission in the mITT Population Efficacy analysis of TSND-201 vs placebo for different CAPS-5 clusters is shown. Criterion B (intrusion) (A), criterion C (avoidance) (B), criterion D (negative alterations in cognitions and mood) (C), or criterion D (arousal and reactivity) (D). E, Rates of the loss of PTSD diagnosis, response (50% improvement or greater), and remission (total CAPS-5 score ≤11). LS indicates least squares; mITT, modified intention to treat; PTSD, posttraumatic stress disorder. ^a^*P* <.05. ^b^*P* <.01.

Across each of the 4 CAPS-5 symptom clusters, TSND-201 demonstrated meaningful improvements over placebo for criterion B (intrusion) (Figure 3A), criterion C (avoidance) (Figure 3B), criterion D (negative alterations in cognitions and mood) (Figure 3C), and criterion E (arousal and reactivity) (Figure 3D)).

TSND-201 treatment resulted in more patients achieving a treatment response (≥50% improvement from baseline on CAPS-5) at day 64 than placebo (57.1% vs 19.2%; treatment difference, 37.9%; 90% CI, 18.0%-57.9%; number needed to treat [NNT] = 3). Remission rates (total score ≤11 on the CAPS-5) at the end of study were greater after treatment with TSND-201 compared with placebo (32.1% vs 11.5%; treatment difference, 20.6%; 90% CI, 2.8%-38.4%; NNT = 5). At the end of study, PTSD diagnostic status was lost in a greater number of TSND-201-treated participants compared with placebo (60.7% vs 30.8%; treatment difference, 29.9%; 90% CI, 8.7%-51.2%; NNT = 3).

Secondary end point analyses revealed that at the end of study, TSND-201 showed a reduction in patient-reported PTSD symptoms on the PCL-5 (−28.46 vs −19.47; LS mean treatment difference, −8.99; 90% CI, −17.81 to −0.17), an improvement in functioning based on the SDS total score (−8.29 vs −3.57; LS mean treatment difference, −4.72; 90% CI, −8.84 to −0.61), and an improvement in depressive symptoms for patients with significant depression symptoms at baseline (MADRS >20; n = 27 and n = 28 for TSND-201 and placebo, respectively) on the MADRS (−13.94 vs −7.73; LS mean treatment difference, −6.21; 90% CI, −12.41 to −0.27).

### Safety

TEAEs were generally mild or moderate. All participants in the TSND-201 group reported at least 1 TEAE, compared with 24 (72.7%) in the placebo group (Table 2). TEAEs were transient, with most occurring on the day of dosing and resolving shortly thereafter. The most commonly reported TEAEs occurring in at least 20% of participants administered TSND-201 were headache, decreased appetite, nausea, dizziness, increased blood pressure, dry mouth, and insomnia.

**Table 2.** Adverse Events (Safety Population)

Preferred term	No. (%)			
	TSND-201		Placebo	
	Anytime	Started during dosing session	Anytime	Started during dosing session
Any AE	32 (100.0)	30 (93.8)	24 (72.7)	19 (57.6)
Severe AE	1 (3.2)	0	0	0
Serious AE	1 (3.2)	0	0	0
AE resulting in study drug discontinuation	0	0	0	0
Treatment-emergent adverse events				
Headache	21 (65.6)	11 (34.4)	14 (42.4)	10 (30.3)
Decreased appetite	16 (50.0)	12 (37.5)	3 (9.1)	2 (6.1)
Nausea	12 (37.5)	7 (21.9)	7 (21.2)	5 (15.2)
Dizziness	11 (34.4)	6 (18.8)	3 (9.1)	3 (9.1)
Blood pressure increased	10 (31.3)	9 (28.1)	0	0
Dry mouth	10 (31.3)	9 (28.1)	1 (3.0)	1 (3.0)
Insomnia	10 (31.3)	1 (3.1)	0	0
Muscle tightness	8 (25.0)	7 (21.9)	1 (3.0)	1 (3.0)
Preexisting condition improved^a^	8 (25.0)	8 (25.0)	2 (6.1)	1 (3.0)
Feeling abnormal	7 (21.9)	5 (15.6)	3 (9.1)	3 (9.1)

Abbreviation: AE, adverse event.

^a^ Due to limitations with MedDRA coding, the following reported terms were coded to “preexisting condition improved” as they related to improvements in their overall condition: feeling optimistic, increased focus, emotional openness, clearheaded, increased openness, increased confidence, and feeling insightful.

There were no significant trends of suicidal ideation or behavior in either treatment group. One unrelated severe AE of suicidal ideation in a participant treated with TSND-201 occurred 6 days after the last dose during follow-up stemming from an isolated family incident. On the C-SSRS, there were no cases of suicidal behavior in either treatment arm and only 1 ideation score of 4 (active ideation with some intent to act without a specific plan) in the placebo arm. There was 1 serious AE of seizure reported within the TSND-201 group, which occurred 7 days after the last dose. The event lasted a few seconds, occurring during a blood draw, and the participant quickly recovered. After the event, she reported experiencing a similar event a few years prior. Based on the participant’s prior history and time since the last dose (>25 half-lives), the event was considered by the investigator as unrelated to the study drug.

### Blinding Questionnaire

When indicating how likely participants thought they received TSND-201 or placebo, 16 patients (53.3%) who received placebo correctly guessed that they likely received placebo, and 21 participants (70%) who received TSND-201 correctly guessed that they likely received active treatment.

## Discussion

In the phase 2 IMPACT-1 trial of TSND-201 in individuals with PTSD, TSND-201 was associated with a statistically significant, clinically meaningful 9.64-point greater reduction in CAPS-5 total score compared with placebo at the end of study. Statistically significant improvements with TSND-201 were observed after the second dose (−8.00 points vs placebo, day 10), continued to decrease and were maintained until the end of the trial (day 64). The current study had broad PTSD criteria, including complex PTSD without restrictions on age of trauma or duration since trauma.

TSND-201 was generally safe and well tolerated with an AE profile consistent with a neuroplastogen with mild stimulant properties and without hallucinogenic effects more typical of psychedelics. Nearly all participants had at least 1 TEAE (100% or 73% in the TSND-201 and placebo groups, respectively), and there were no study discontinuations due to TEAEs. The most common TEAEs in the TSND-201 group were mild or moderate in intensity, occurred on the day of dosing, and resolved within a day.

Improvements on secondary end points (ie, patient-reported PTSD symptoms [PCL-5], functioning [SDS], and depression [MADRS]) were observed with TSND-201 treatment, demonstrating consistent therapeutic benefit across multiple domains typically affected by PTSD.

Despite its short (approximately 6 hours) half-life,^7^ weekly dosing with TSND-201 produced clinical effects lasting 6 weeks after the final dose. This durability may be due to its activity as a rapid-acting neuroplastogen, which promotes synaptic plasticity, neurite outgrowth, and neurotrophin expression^10,16^—processes disrupted by PTSD.^28,29,30^ Rapid and durable effects on neuroplasticity mechanisms may account for its prolonged efficacy and distinguishes TSND-201 from traditional antidepressants that require continuous daily dosing.

Although structurally related to MDMA, TSND-201 has distinct pharmacological and subjective effects^7,8,9,10^ likely due to greater selectivity for the monoamine transporters.^10,11,12,13,14,15^ Unlike MDMA, which is used as an adjunct to psychotherapy,^5,6^ there was no psychotherapy accompanying TSND-201 treatment in the current trial—raising the question of how such a brief, intermittent dosing regimen yields durable outcomes. One explanation is that TSND-201-induced neuroplasticity,^10,16^ a mechanism that underlies the activity of classic antidepressants and rapid-acting treatments in development,^31,32^ drives rapid and lasting changes in the brain circuitry that is affected by PTSD.^28,33^

### Limitations

In the IMPACT-1 trial, there were a limited number of individuals with military trauma, a cohort that shows high rates of PTSD^34,35^ and typically shows poorer treatment outcomes compared with civilians.^36^ The generalizability of the results may also be limited by the high rate of screening failures. Also, for statistical analyses, a 1-sided *P* value was used due to this being a small phase 2 study; however, statistical significance is maintained for the primary (and additional) end points when a 2-sided test is applied. Finally, due to the sample size, it was not possible to draw definitive conclusions regarding predictors of responsiveness that have been considered in prior studies (eg, type of trauma, age at which trauma occurred, single vs multiple trauma, etc). This will be examined more closely in future, larger studies of TSND-201.

Blinding questionnaires are becoming more common in clinical trials involving psychoactive medications. Our results suggest that 70% of participants were able to correctly guess that they received active treatment. This proportion of participants is similar to a recent meta-analysis of antidepressants, which reported between 45% and 71% of participants correctly guessed their treatment.^37^

## Conclusions

In the IMPACT-1 randomized clinical trial including adults with severe PTSD, intermittent treatment with TSND-201 (4 doses, 1 week apart) was generally well tolerated, safe, and associated with statistically significant and clinically meaningful improvements in PTSD symptoms that persisted well beyond the initial dosing period. Results support the further development of TSND-201 as a potential acute, intermittent treatment for PTSD. Together, these results demonstrate that TSND-201 has the potential to be a highly efficacious, rapid-acting and durable medication for PTSD. These promising results will require replication in larger upcoming studies.

## Notes

Supplement 1.
Trial Protocol.

Supplement 2.
Statistical Analysis Plan.

Supplement 3. **eAppendix.** Part A of IMPACT-1 (Open-Label) Overview and Results **eTable 1.** Demographics and Baseline Characteristics (IMPACT-1, Part A) **eFigure 1.** CAPS-5 Change from Baseline Over Time (IMPACT-1, Part A) **eFigure 2.** Response and Remission on CAPS-5 at Day 64 (IMPACT-1, Part A) **eTable 2.** Treatment-Emergent AEs Occurring in >1 Participant (IMPACT-1, Part A)

Supplement 4.
Data Sharing Statement.
