# Supplementary material for: Efficacy and Safety of the Neuroplastogen TSND-201 for the Treatment of PTSD: A Randomized Clinical Trial
Source: JAMA Psychiatry. 2026 Feb 18;83(5):469–77. doi: 10.1001/jamapsychiatry.2025.4625 (PMC12917749; doi:10.1001/jamapsychiatry.2025.4625)
Supplement: Supplement 1. — Trial Protocol. [file jamapsychiatry-e254625-s001.pdf]

Clinical Study Protocol

A Pilot Study to Assess the Use of Methylone in the Treatment of PTSD
IMPACT-1 (Investigation of Methylone for Post-Traumatic Stress Disorder [PTSD])

Investigational Medicinal Product: Methylone

Sponsor Reference: TT-TSND-201

IND Number: 167109

Amendment #7 Protocol Version and Date: V 6.0, USA, 11 Oct 2024
Amendment #6 Protocol Version and Date: V 5.2, USA, 15 Jul 2024
Amendment #5 Protocol Version and Date: V 5.1, USA, 17 May 2024
Amendment #4 Protocol Version and Date: V 5.0, 08 Feb 2024
Amendment #3 Protocol Version and Date: V 4.0, 21 Apr 2023
Amendment #2 Protocol Version and Date: V 3.1, 11 Oct 2022
Amendment #1 Protocol Version and Date: V 2.0, 08 Jun 2022
Original Protocol Version and Date: V 1.0, 11 March 2022
Clinical Development Phase: 1b/2a

Sponsor: Transcend Therapeutics
80 5th Avenue, 18th Floor
New York, New York
10011
United States of America

CONFIDENTIALITY STATEMENT

The information provided in this document is strictly confidential and is available for review to Investigators, potential Investigators, appropriate ethics committees and other national authorities. No disclosure should take place without the written authorisation from the Sponsor, except to the extent necessary to obtain informed consent from potential participants.

SPONSOR SIGNATURE PAGE

For and on behalf of the Study Sponsor:

Signatures:

[redacted]

Site Investigator Signature Page

PROTOCOL NUMBER:	TT-TSND-201
PROTOCOL TITLE:	A Pilot Study to Assess the Use of Methylone in the Treatment of PTSD<br>IMPACT-1 (Investigation of Methylone for Post-Traumatic Stress Disorder [PTSD])
Protocol Date and Version:	US V6.0, 11Oct2024

The undersigned confirms that the following Protocol has been agreed and accepted and that the Site Investigator agrees to conduct the study in compliance with the approved Protocol and in accordance with the revised Declaration of Helsinki 1996. I will adhere to all ICH, GCP, FDA and local applicable regulations and guidelines regarding clinical studies during and after study completion. I will ensure that all sub-investigators and other study staff members have read and understand all aspects of this protocol. I agree to cooperate fully with Transcend and its designated vendors during the study. I agree to ensure that the confidential information contained in this document will not be used for any other purpose other than the evaluation or conduct of the clinical study without the prior written consent of the Sponsor.

I also confirm that I will, when required by the Sponsor, make the findings of the study publicly available through publication or other dissemination tools without any unnecessary delay and that an honest accurate and transparent account of the study will be provided; and that any discrepancies and serious breaches of GCP from the study as planned in this Protocol will be explained.

Site Investigator Name:

Signature:

.... Date:....../....../......

KEY STUDY CONTACTS

Sponsor	Transcend Therapeutics<br>80 5th Avenue, 18th Floor<br>New York, NY<br>10011<br>United States of America
Sponsor Contact	[redacted]<br><br>[redacted]

TABLE OF CONTENTS

SPONSOR SIGNATURE PAGE **2**
KEY STUDY CONTACTS **4**
TABLE OF CONTENTS **5**
LIST OF TABLES **9**
1 PROTOCOL SUMMARY **10**
1.1 Synopsis 10
1.2 Study Design Schema 14
1.3 Schedule of Assessments (SoA) 15
1.4 List of Abbreviations 17
2 INTRODUCTION **19**
2.1 Background 19
2.1.1 Post-Traumatic Stress Disorder *19*
2.2 Study Rationale 20
2.3 Nonclinical Development 20
2.4 Clinical Development 21
2.4.1 Completed Open-Label Study in PTSD Patients (IMPACT-1 Part A) *21*
2.4.2 Completed Healthy Volunteer Studies *22*
2.4.3 Retrospective Case Series *23*
3 OBJECTIVES AND ENDPOINTS **25**
4 STUDY DESIGN **28**
4.1 Study Design Overview 28
4.2 Study Visit Breakdown 29
4.3 Planned Duration of Study 34
4.4 Rationale for Study Design 35
4.5 Dose Rationale 35
4.6 End of Study 37
4.6.1 End-of-Study Definition *37*
4.6.2 Exit Plan *37*
4.7 Data and Safety Monitoring Board 37
4.8 Criteria For Termination of Dosing for An Individual Participant 38
4.9 Criteria for Interruption of Randomization/Dosing of New Participants 39
5 STUDY TREATMENTS **41**
5.1 Name and Description of Investigational Product(s) 41
5.2 Study Drug Administration 41
5.3 Psychological and Medical Support on Dosing Days 41

5.3.1 Psychological Support 41
5.3.2 Medical Support 42
5.4 Discharge Criteria 42
5.5 Concomitant Medication 43
5.5.1 Prohibited Medications 43
5.6 Special Warnings and Guidance 44
5.6.1 General Considerations 44
5.6.2 Recorded Content 44
5.6.3 Management of Suicidality 44
5.6.4 Management of Psychological Events 44
5.6.5 Management of Cardiovascular Events 45
5.6.6 Management of Thermoregulation 45
5.6.7 Management of Osmoregulation 46
5.7 Assessment of Compliance with Treatment 46
5.8 Blinding 46
5.9 Emergency Unblinding 46
6 SELECTION OF PARTICIPANTS 47
6.1 Inclusion Criteria 47
6.2 Exclusion Criteria 48
6.3 Study Restrictions 50
6.3.1 Lifestyle Restrictions 50
6.3.2 Meals and Dietary Restrictions 51
6.4 Participant Withdrawal Criteria 51
6.5 Follow-Up Procedures 52
7 SAFETY ASSESSMENTS 53
7.1 Clinical Laboratory Evaluations 53
7.2 12-Lead Electrocardiogram 53
7.3 Vital Signs and Temperature 54
7.4 Physical Examinations 54
7.5 Pregnancy Testing 54
7.6 Columbia-Suicide Severity Rating Scale 54
7.7 Adverse Event Reporting 55
7.7.1 Definition and Criteria 55
7.8 Evaluating Adverse Events and Serious Adverse Events 57
7.8.1 Reporting Procedures and Requirements 58
7.8.2 Prompt Reporting of Serious Adverse Events 60

7.8.3 Special Considerations 60
7.9 Other Assessments 61
8 PSYCHOMETRIC ASSESSMENTS 62
8.1 Clinician-Administered Psychometric Assessments 62
8.1.1 Clinician-Administered PTSD Scale for DSM-5 (CAPS-5) 62
8.1.2 Mini International Neuropsychiatric Interview 62
8.1.3 Structured Clinical Interview for DSM-5 Personality Disorders (SCID-5-PD) 62
8.1.4 Structured Interview Guide for the Montgomery-Åsberg Depression Rating Scale (MADRS) (SIGMA) 63
8.1.5 Clinician Global Impressions Scale – Severity 63
8.1.6 Clinician Global Impressions Scale – Improvement 63
8.2 Participant-Completed Psychometric Assessments 63
8.2.1 PTSD Checklist for DSM-5 (PCL-5) 63
8.2.2 Life Events Checklist for DSM-5 (Extended Version) 63
8.2.3 Structured Clinical Interview for DSM-5 Screening Personality Questionnaire (SCID-5-SPQ) 64
8.2.4 Sheehan Disability Scale 64
8.2.5 Patient Global Impression of Change 64
8.2.6 Patient Global Impression of Severity 64
8.2.7 Warwick-Edinburgh Mental Wellbeing Scale 64
8.2.8 Post-Traumatic Growth Inventory 64
8.2.9 Beck Depression Inventory-II 64
8.3 Sleep Quality Assessments 65
8.3.1 Pittsburgh Sleep Quality Index 65
[redacted]
8.4.3 Blinding Integrity Questionnaire (Part B only) 65
9 STATISTICS AND DATA ANALYSIS 66
9.1 Randomization and Participant Allocation 66
9.2 Sample Size Determination 66
9.3 Statistical Analysis Plan 66
9.4 Analysis Sets 66
9.4.1 Modified Intent-to-Treat (mITT) Population 66
9.4.2 Per-Protocol Population 67
9.4.3 Safety Population 67

9.5 Statistical Analysis ... 67
9.5.1 Safety Statistical Analysis ... 67
9.5.2 Efficacy Statistical Analysis ... 68
9.6 Handling of Missing or Incomplete Data ... 68
10 DATA HANDLING AND RECORD KEEPING ... 69
10.1 Collection of Data ... 69
10.2 Inspection of Records ... 69
10.3 Retention of Records ... 69
10.4 Confidentiality and Data Protection ... 70
11 MONITORING, AUDIT, AND INSPECTION ... 71
11.1 Study Monitoring ... 71
11.2 Audits and Inspections ... 71
11.3 Quality Control and Quality Assurance ... 71
12 ETHICAL AND REGULATORY CONSIDERATIONS ... 72
12.1 Ethical Conduct of the Study ... 72
12.2 Approval by Institutional Review Board / Ethics Committee ... 72
12.3 Written Informed Consent ... 72
13 REFERENCES ... 73
14 APPENDICES ... 76
14.1 Appendix 1– Sampling Summary ... 76
14.2 Appendix 2 – Prohibited Medications – Psychoactive Substances ... 77
14.3 Appendix 3 – Prohibited Medications/Elimination Half-Life - Drug Interactions ... 78
14.4 Appendix 4 – Childbearing Potential ... 80

LIST OF TABLES

Table 1: Objectives and Endpoints for Part A 25
Table 2: Objectives and Endpoints for Part B 26
Table 3: Study Interventions Administered 41
Table 4: Clinical Laboratory Evaluations 53

1 PROTOCOL SUMMARY

1.1 Synopsis

Study Title: A Pilot Study to Assess the Use of Methylone in the Treatment of PTSD
IMPACT-1 (Investigation of Methylone for Post-Traumatic Stress Disorder [PTSD])

Investigational Medicinal Product: Methylone

Clinical Phase: 1b / 2a

Rationale:
Methylone is a new and potentially effective treatment option for participants with PTSD. The purpose of this pilot study is to evaluate the safety, tolerability, and efficacy of methylone in adult participants with PTSD. The study will be conducted in two parts. Part A will be open-label and will enroll approximately 15 evaluable participants with PTSD in the United States (US), United Kingdom (UK) and Europe. After completion of Part A, enrollment will begin for Part B, which will be double-blind, placebo-controlled multi-site global study and will enroll approximately 64 evaluable participants with PTSD.

Objectives and Endpoints:
This is a two-part study with Part A focusing on safety as the primary objective and Part B focusing on efficacy as the primary objective. Therefore, each Part of the study has separate objectives and endpoints as presented in the tables below.

**Part A**	
**Objective**	**Endpoints**
**Primary**	
To assess the safety and tolerability of oral methylone administered weekly over 4 weeks in participants with PTSD.	• Incidence and severity of TEAEs.<br>• Incidence and severity of AESIs.<br>• Change in HR, SBP, DBP and temperature.<br>• Clinically significant changes in ECG.<br>• Changes from baseline in clinical laboratory parameters (clinical chemistry, hematology and urinalysis).
**Secondary**	
To assess the efficacy of methylone in treating PTSD symptoms.	Mean change from baseline to Week 10 in CAPS-5 (total severity score assessed over 1 week)<br><br>Percentage of participants having:<br>• Treatment response, defined as a<br>o ≥ 10 point reduction on the CAPS-5 from baseline<br>o 30% improvement from baseline on CAPS-5<br>o 50% improvement from baseline on CAPS-5<br>• Remission, defined as a score of ≤ 11 on the CAPS-5
To assess the effect of methylone on sleep quality, functional disability, treatment satisfaction, quality of life, and physical function in participants with PTSD.	Mean change from baseline in the following scales:<br>• CGI-S<br>• MADRS<br>• SDS<br>• PCL-5<br>• PGI-S<br>• BDI-II<br>• WEMWBS<br>• PSQI

	Percentage of participants with improvement on the following scales:<br>• PGI-C<br>• CGI-I
[redacted]	
[redacted]	• [redacted]<br>• PTGI

Part B	
**Objectives**	**Endpoints**
**Primary**	
To assess the efficacy of methylone in treating PTSD symptoms.	Mean change from baseline to Week 10 compared with placebo in CAPS-5 total severity score.
**Secondary**	
To assess the effect of methylone compared to placebo on sleep quality, functional disability, treatment satisfaction, quality of life, and physical function in participants with PTSD.	Mean change from baseline compared with placebo in the following scales:<br>• CGI-S<br>• MADRS<br>• SDS<br>• PCL-5<br>• PGI-S<br>• BDI-II<br>• WEMWBS<br>• PSQI<br><br>Percentage of participants having:<br>• Treatment response, defined as a<br>○ ≥ 10 point reduction on the CAPS-5 from baseline<br>○ 30% improvement from baseline on CAPS-5<br>○ 50% improvement from baseline on CAPS-5<br>• Remission, defined as a score of ≤ 11 on the CAPS-5<br>• Improvement on the PGI-C<br>• Improvement on the CGI-I
To assess the safety and tolerability of oral methylone compared to placebo administered weekly over 4 weeks in participants with PTSD.	• Incidence and severity of TEAEs<br>• Incidence and severity of AESIs<br>• Change in HR, SBP, DBP and temperature during each dosing session<br>• Clinically significant changes in ECG<br>• Changes from baseline in clinical laboratory parameters (clinical chemistry, hematology, and urinalysis)
[redacted]	
[redacted]	Effect of the following scales, compared with placebo:<br>• PTGI<br>• [redacted]

Abbreviations: [redacted] AE = adverse event; AESI = adverse event of special interest; BDI-II = Beck Depression Inventory-II; DBP = diastolic blood pressure; CAPS-5 = Clinician-Administered PTSD Scale for DSM-5; CGI-I = Clinician Global Impression of Improvement; CGI-S = Clinical Global Impression of Severity; C-SSRS = Columbia Suicide Severity Rating Scale; DBP = diastolic blood pressure; ECG = electrocardiogram; HR = heart rate; LEC-5 = Life Events Checklist for DSM-5; MADRS = Montgomery-Åsberg Depression Rating Scale; [redacted] MINI = Mini International Neuropsychiatric Interview; PCL-5 = PTSD Checklist for DSM-5; PGI-C = Patient Global Impression of Change; PGI-S = Patient Global Impression of Severity; PSQI = Pittsburgh Sleep Quality Index; PTGI = Post-traumatic Growth Inventory; PTSD = Post-traumatic stress disorder; SDS = Sheehan Disability Scale; SBP = systolic blood pressure; TEAEs = treatment-emergent adverse events; WEMWBS = Warwick-Edinburgh Mental Wellbeing Scale.

Overall Design

Brief Summary:

This is a two-part study to assess methylone for the management of the symptoms of PTSD. Part A is an open-label, non-controlled assessment in approximately 15 evaluable participants with PTSD to assess early safety and efficacy and to confirm procedures included in the blinded portion (Part B) of the study. After completion of Part A, enrollment for Part B will begin in approximately 64 participants. Unless otherwise stated, Part B is identical to Part A with the exception of the inclusion of a placebo arm in Part B.

The open-label treatment in Part A is:

- Methylone 150 mg, with a booster administration of 100 mg administered 90 (±10) minutes after the initial administration, during each dose session.

Part B is a randomized, double-blind, parallel-group, placebo-controlled assessment of methylone for the management of the symptoms of PTSD. This multi-center study will be conducted in up to 25 sites globally. Planned enrollment includes approximately 79 evaluable participants (approximately 15 in Part A and approximately 64 in Part B). There are 4 planned dose sessions for each participant. Participants in Part B will be randomized 1:1 to the two study treatment arms and will receive the randomized treatment at each of the weekly dose sessions for the duration of the study.

The two blinded study treatment arms in Part B are:

- Methylone 150 mg, with a booster administration of 100 mg administered 90 (±10) minutes after the initial administration, during each dose session.
- Matched placebo at each timepoint (initial + booster) during each dose session.

For each participant in Parts A and B, the study will consist of:

- **Screening Period (Day -28 to Baseline):** Informed consent, eligibility assessment, and enrollment of eligible participants.
- **Baseline/Preparatory Session (Day -4 to Day -1):** Baseline assessments, confirmation of eligibility, and a preparatory psychoeducation session leading to enrollment confirmation.
- **Treatment Period (Day 1 to Day 24):** Four weekly dose sessions, with associated remote sessions. The dose sessions will last at least 8 hours, and until all effects (physical and psychological) have resolved (whichever is longer). Each dose session will be followed by a safety phone call 1 day after dosing and efficacy assessments 2 days after dosing. Each dosing period will be recorded for quality and training purposes. The recordings may be reviewed to ensure the dosing session monitor is adhering to the dosing session monitor training.
- **Follow-Up Period (Day 29 to Day 64):** Follow-Up visits for safety and efficacy will occur at 1, 2, 3 and 6 weeks post-final study drug administration. In addition, there will be Reflection Sessions 1, 2 and 3 weeks post-final study drug administration. Participants will be contacted via telephone on Day 57 (±2 days) for Follow-Up.

The expected duration of participation for each participant who completes all study visits, from Screening to the EOS visit, is up to 16 weeks.

Study Participants:

Medically healthy adult participants aged between 18 to 65 years, inclusive. Participants must meet the diagnostic and statistical manual of mental disorders, fifth edition (DSM-5) criteria for current moderate to severe PTSD diagnosis with a symptom duration of at least 6 months at Screening, as assessed by the CAPS-

5 and the Life Events Checklist for DSM-5 (LEC-5) and must have failed at least one treatment for PTSD (either psychotherapy or pharmacological treatment).<br>In addition, participants must not have a primary diagnosis of any other DSM-5 disorder, as assessed by the MINI Version 7.0.2, or have any history, physical or psychological symptoms, medication or other relevant findings that would make a participant unsuitable for the study based on the clinical judgement of study personnel.
**Treatment Duration:**<br>The treatment duration for both Parts A and B will be the same: four weeks as weekly dosing (Day 1, Day 8 [±1 day], Day 15 [±1 day], and Day 22 [±1 day]).
**Data Monitoring**<br>The objective of the Data and Safety Monitoring Board (DSMB) is to provide an independent review of safety data (Parts A and B) during the clinical trial.<br>DSMB meetings will be scheduled to occur after 8 participants in Part A have completed the Day 29 Follow-Up visit, or EOS if the participant withdraws early (Safety Run-in Phase), and once at the conclusion of enrollment in Part A and after all participants in Part A have the opportunity to complete the Day 29 Follow-Up visit.<br>For Part B, DSMB meetings will occur at intervals of approximately 20 participants completing the Day 29 Follow Up Visit or EOS if the participant withdraws early. Ad hoc DSMB meetings can occur if needed.
**Dose and Route of Administration:**<br>Participants will be fasted for 2 hours predose and until 2 hours after the initial administration during each dose session. Methylone (or matched placebo in Part B) will be orally administered on Day 1, Day 8 (±1 day), Day 15 (±1 day) and Day 22 (±1 day). An initial administration of 150 mg methylone (or matched placebo in Part B) will be given, followed by a booster administration of 100 mg methylone (or matched placebo in Part B) 90 (±10) minutes later.
**Criteria for Evaluation:**<br>Safety will be assessed through adverse event (AE) reporting, monitoring of AESIs, 12-lead ECG, vital signs, physical examinations and clinical laboratory evaluations; suicidal ideation and behavior will be evaluated using the Columbia-Suicide Severity Rating Scale (C-SSRS).<br><br>Efficacy will be assessed through several psychometric assessments and scales that assess PTSD symptoms and quality of life.<br><br>In addition, assessments will be made of sleep quality, treatment satisfaction, and psychedelic effects.
**Statistical Analysis:**<br>Safety parameters will be listed and summarized using descriptive statistics for Parts A and B.<br><br>All efficacy endpoint data will be listed for individual participants. All continuous efficacy endpoints will also be summarized by descriptive statistics (n, arithmetic mean, standard deviation [SD], median, minimum, and maximum) by study part, overall, and by treatment (for Part B) and study period. All categorical efficacy endpoints will be summarized by descriptive statistics (n, percentage).<br><br>For Part A the change from baseline to each timepoint will be presented along with a Paired *t*-test p-value.<br><br>A Mixed Model for Repeated Measures (MMRM) will be fitted for select efficacy endpoints to analyze the difference between treatments for Part B. The model will include treatment, visit, and treatment by visit interaction as fixed effects, where visit is the repeated effect. The baseline value will be included (if applicable) as a covariate. All tests will be based on two-sided significant level of 0.05.

1.2 Study Design Schema

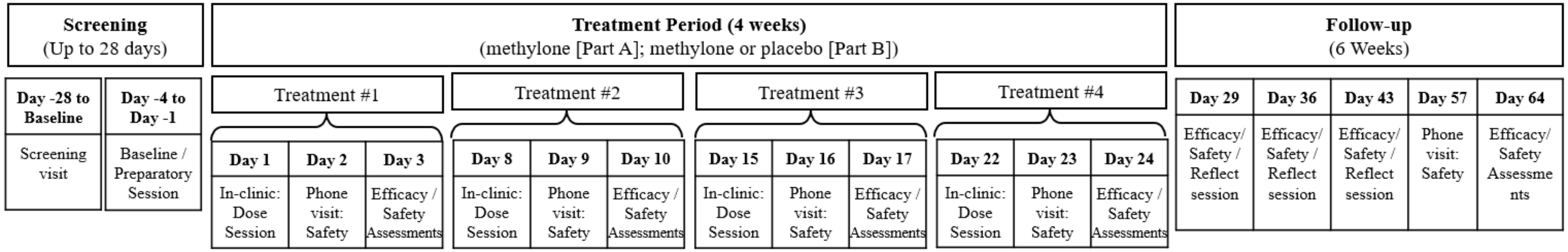

1.3 Schedule of Assessments (SoA)

	**Screening**		**Treatment Period**												**Follow-Up Period**					**ET**
**Visit Number**	**1**	**2**	**3**	**4**	**5**	**6**	**7**	**8**	**9**	**10**	**11**	**12**	**13**	**14**	**15**	**16**	**17**	**18**	**19**	**-**
**Visit Session**	**Scrn**	**BL/ Prep**	**Treatment 1**			**Treatment 2**			**Treatment 3**			**Treatment 4**			**FU1**	**FU2**	**FU3**	**TC**	**FU4 /EOS**	**ET visit**
**Study Day(s)**	**-28 to BL**	**-4 to -1**	**1**	**2**	**3 (+1)**	**8 (±1)**	**9**	**10 (+1)**	**15 (±1)**	**16**	**17 (+1)**	**22 (±1)**	**23**	**24 (+1)**	**29 (±1)**	**36 (±2)**	**43 (±2)**	**57 (±2)**	**64 (±3)**	**-**
ICF	X																			
Demography	X																			
Confirm Eligibility	X	X	X																	
Med Hx	X																			
Substance Use Hx	X																			
Drug Use Assessment[1]	X	X	X			X			X			X								
Pregnancy Test[2]	X		X			X			X			X			X					X[3]
Physical Exam	X[4]														X				X[4]	X[3]
Vital Signs[5]	X	X	X[6]			X[6]			X[6]			X[6]			X					X[3]
12-Lead ECG	X		X[7]			X[7]			X[7]			X[7]			X					X[3]
Clinical Labs[8]	X								X						X					X[3]
MINI (central rater)	X																			
LEC-5	X																			
SPQ	X																			
SCID-5-PD (Central rater)[12]	X																			
CAPS-5 (past month; central rater)	X																			
C-SSRS[9]	X	X	X	X	X	X	X	X	X	X	X	X	X	X	X	X	X	X	X	X
Preparatory Session[10]		X																		
Randomization[11]			X																	
Study Drug Admin[13]			X			X			X			X								

[1] Urine drug screen must be confirmed as negative prior to enrollment and each dose. Site must confirm patient is not otherwise impaired prior to dosing.
[2] Urine test for WOCBP only. Pregnancy test must be confirmed as negative prior to enrollment and each dose. Serum FSH may be conducted at Screening to confirm non-childbearing potential.
[3] Only required if the ET visit occurs prior to the Day 29 visit.
[4] Height and weight will be collected at Screening; weight will be conducted at the end of study.
[5] Includes heart rate, blood pressure, and temperature.
[6] Vital signs during the dose session: BP and HR - predose, every 30-minutes post-initial dose, and prior to the booster dose; temperature- predose (within 10 min), and every 60 minutes.
[7] ECGs will be captured predose and at the end of the dosing session.
[8] Clinical labs will be assessed by a central lab.
[9] C-SSRS should be conducted at any unscheduled visits.
[10] To be performed after all baseline assessments.
[11] Subjects enrolled in Part B only.
[12] Completed only if participant endorses a positive response to paranoid personality disorder, antisocial personality disorder, and borderline personality disorder on the SPQ.

	Screening		Treatment Period												Follow-Up Period					ET
**Visit Number**	**1**	**2**	**3**	**4**	**5**	**6**	**7**	**8**	**9**	**10**	**11**	**12**	**13**	**14**	**15**	**16**	**17**	**18**	**19**	**-**
**Visit Session**	**Scrn**	**BL/ Prep**	**Treatment 1**			**Treatment 2**			**Treatment 3**			**Treatment 4**			**FU1**	**FU2**	**FU3**	**TC**	**FU4 /EOS**	**ET visit**
**Study Day(s)**	**-28 to BL**	**-4 to -1**	**1**	**2**	**3 (+1)**	**8 (±1)**	**9**	**10 (+1)**	**15 (±1)**	**16**	**17 (+1)**	**22 (±1)**	**23**	**24 (+1)**	**29 (±1)**	**36 (±2)**	**43 (±2)**	**57 (±2)**	**64 (±3)**	**-**
[redacted]			[redacted]			[redacted]			[redacted]			[redacted]								
AEs			X	X	X	X	X	X	X	X	X	X	X	X	X	X	X	X	X	X
Con Meds	X	X	X	X	X	X	X	X	X	X	X	X	X	X	X	X	X	X	X	X
CAPS-5 (past-week; central rater)		X			X			X			X			X	X	X	X		X	X
MADRS (central rater)		X			X			X			X			X	X	X	X		X	X
CGI-S (central rater)		X			X			X			X			X	X	X	X		X	X
CGI-I (central rater)					X			X			X			X	X	X	X		X	X
PCL-5	X	X			X			X			X			X	X	X	X		X	X
BDI-II		X									X				X		X		X	X
SDS		X			X			X			X			X	X	X	X		X	X
WEMWBS		X									X				X		X		X	X
PGI-S		X			X			X			X			X	X	X	X		X	X
PGI-C					X			X			X			X	X	X	X		X	X
PSQI		X													X				X	X
PTGI		X													X				X	X
BIQ[11]															X					X
Reflection session															X	X	X			

Abbreviations: [redacted] AE = adverse event; BDI-II = Beck Depression Inventory-II; BIQ = blinding integrity questionnaire; BL = baseline; BP = blood pressure; CAPS-5 = Clinician-Administered PTSD Scale for DSM-5; CGI-I = Clinician Global Impression of Improvement; CGI-S = Clinical Global Impression of Severity; C-SSRS = Columbia Suicide Severity Rating Scale; ECG = electrocardiogram; EOS = end of study; ET = early termination; FSH = follicle stimulating hormone; FU = follow up; HR = heart rate; LEC-5 = Life Events Checklist for DSM-5; MADRS = Montgomery-Åsberg Depression Rating Scale; MINI = Mini International Neuropsychiatric Interview; [redacted] PCL-5 = PTSD Checklist for DSM-5; PGI-C = Patient Global Impression of Change; PGI-S = Patient Global Impression of Severity; PSQI = Pittsburgh Sleep Quality Index; PTGI = Post-traumatic Growth Inventory; Scrn = screening; SCID-5-PD = Structured Clinical Interview for DSM-5 Personality Disorders; SDS = Sheehan Disability Scale; SPQ = Screening Personality Questionnaire; TC = telephone call; WEMWBS = Warwick-Edinburgh Mental Wellbeing Scale; WOCBP = women of childbearing potential.

[13] Study drug is administered in clinic and patients are monitored for at least 8 hours following each dose. The dose is given in two divided doses: an initial dose, followed by a booster dose 90 minutes later.

1.4 List of Abbreviations

Abbreviation	Definition
[redacted]	[redacted]
5-HT	serotonin
AE	adverse event
ADHD	attention deficit hyperactivity disorder
ADL	activities of daily living
AESI	Adverse Events of Special Interest
ALT	alanine aminotransferase
AST	aspartate aminotransferase
ARCI	Addiction Research Center Inventory
BDI-II	Beck Depression Inventory – II
BIQ	Blinding Integrity Questionnaire
BMI	body mass index
BP	blood pressure
CAPS-5	Clinician-Administered PTSD Scale for DSM-5
CGI-I	Clinician Global Impressions Scale – Improvement
CGI-S	Clinician Global Impressions Scale – Severity
CNS	central nervous system
C-SSRS	Columbia-Suicide Severity Rating Scale
CTCAE	Common Terminology Criteria for Adverse Events
DA	dopamine
DAT	dopamine transporters
DBP	diastolic blood pressure
DED	dread of ego dissolution
DMT	dimethyltryptamine
DSMB	Data and Safety Monitoring Board
DSM-5	Diagnostic and Statistical Manual of Mental Disorders, Fifth Edition
EC	ethics committee
ECG	electrocardiogram
eCRF	electronic case report form
eGFR	glomerular filtration rate
EOS	End-of-Study
FSH	follicle stimulating hormone
GCP	Good Clinical Practice
GGT	gamma-glutamyl transferase
GLP	Good Laboratory Practice
HR	heart rate
HRT	hormonal replacement therapy
HV	healthy volunteers
IB	Investigator Brochure
ICH	International Council for Harmonisation
ICF	informed consent form
IM	intramuscularly
IMP	investigational medicinal product
IRB	institutional review board
ITT	Intention-to-Treat
LEC-5	Life Events Checklist for DSM-5
LFTs	Liver Function Tests
LSD	lysergic acid diethylamide
MADRS	Montgomery-Åsberg Depression Rating Scale
MAOI	monoamine oxidase inhibitors
MDD	major depressive disorder
MedDRA	Medical Dictionary for Regulatory Activities

Abbreviation	Definition
[redacted]	[redacted]
MDMA	3,4-methylenedioxymethamphetamine
MDPV	methylenedioxypyrovalerone
MINI	Mini International Neuropsychiatric Interview
MMRM	Mixed Model for Repeated Measures
NCS	non-clinically significant
NE	norepinephrine
NET	Norepinephrine transporters
OB	oceanic boundlessness
OTC	over-the-counter
PCL-5	PTSD Checklist for DSM-5
PGI-C	Patient Global Impression of Change Scale
PGI-S	Patient Global Impression of Severity Scale
PK	pharmacokinetic
PSQI	Pittsburgh Sleep Quality Index
PT	preferred term
PTSD	post-traumatic stress disorder
PTGI	post-traumatic growth inventory
QTcF	QT interval corrected for heart rate using Fridericia's formula
REC	research ethics committee
SAE	serious adverse event
SAP	Statistical Analysis Plan
SBP	systolic blood pressure
SCID-5-PD	Structured Clinical Interview for DSM-5 Personality Disorders
SD	standard deviation
SDS	Sheehan Disability Scale
SERT	serotonin transporter
SIGMA	Structured Interview Guide for the MADRS
SNRI	serotonin and norepinephrine reuptake inhibitors
SOC	system-organ class
SOP	Standard Operating Procedure
SPQ	Screening Personality Questionnaire
SSRI	selective serotonin reuptake inhibitor
SUSAR	Suspected Unexpected Serious Adverse Reactions
TEAE	treatment-emergent adverse event
ULN	upper limit of normal
VESSPA-SS	Evaluation of Subjective Effects of Substances with Abuse Potential questionnaire
VMAT-2	vesicular monoamine transporter 2
VRS	visionary re-structuralization
WEMWBS	Warwick-Edinburgh Mental Wellbeing Scale
WHO	World Health Organization
WOCBP	woman of childbearing potential
WONCBP	woman of non-childbearing potential

2 INTRODUCTION

2.1 Background

Methylone (3,4-methylenedioxy-N-methylcathinone) is a synthetic empathogen, psychostimulant and member of the drug class known as synthetic cathinones (Poyatos 2021, Poyatos 2022, Poyatos 2023, WHO 2014). Methylone acts by altering the function of plasma membrane transporters for serotonin (5-HT), dopamine (DA), and norepinephrine (NE) (Baumann 2018, Cameron 2013). Methylone and 3,4-methylenedioxymethamphetamine (MDMA) are structurally and pharmacologically similar but have distinct differences in potency and activity. Both methylone and MDMA act as mixed reuptake inhibitor/releasing agents. However, methylone has lower effects on serotonin (4x) and dopamine (3x) but a greater (2x) effect on norepinephrine release compared to MDMA. In addition, methylone's affinity for the vesicular monoamine transporter 2 ($VMAT_2$) is about 13 times lower than that of MDMA (Eshleman 2013). Unlike MDMA, methylone does not directly interact with $5\text{-}HT_{2A}$ receptors which likely contributes to the lack of hallucinogenic effect. These results suggest that the subjective experience and physiological effects of methylone may be less pronounced than MDMA.

Methylone was first synthesized and proposed as a potential antidepressant in the 1990s (Shulgin 1996). Based on healthy volunteer and nonclinical studies, methylone has physiological effects that may translate into a fast acting treatment for psychiatric disorders such as PTSD. Nonclinical studies show that methylone has fast-acting, robust, long-lasting anxiolytic and antidepressant-like activity (Warner-Schmidt 2023). Methylone also facilitates fear extinction in a preclinical model of PTSD (Yu 2022), like MDMA (Baumann 2018) and is consistent with the potential therapeutic benefit. Data from studies in healthy volunteers (Poyatos 2021, Poyatos 2022, Poyatos 2023) suggest a shorter acting pharmacokinetic profile compared with MDMA, which is further is supported by uncontrolled clinical use in a case series of methylone in patients with PTSD (Kelmendi 2022).

Methylone likely involves MDMA-like entactogenic effects with a strong sense of emotional openness, enhanced empathy and reduced fear. These physiological effects could be particularly well-suited as treatment for persistent neuropsychiatric disorders characterized by excessive anxiety, fear, or that arise following exposure to traumatic events, potentially with milder side effects than is seen with MDMA.

2.1.1 Post-Traumatic Stress Disorder

Post-traumatic stress disorder (PTSD) is a serious debilitating disorder that occurs in people who have experienced or witnessed a traumatic event such as a natural disaster, a serious accident, or who have been threatened with death, sexual violence, or serious injury. Current estimates suggest that 1 in 3 people that experience a traumatic event will be affected by PTSD, however there is no clear biomarker that explains why some people develop the condition and others do not (NHS 2023). Post-traumatic stress disorder has a point prevalence estimated at nearly 3% in the UK (McManus 2009). International studies of lifetime prevalence show considerable variations in rates between nations, however it is estimated that 6.8% of US adults and one in 11 people in the UK will be diagnosed with PTSD in their lifetime (McManus 2009, NIMH 2023, Greenberg 2015). Symptoms of PTSD include intrusive thoughts and memories, negative effects on cognition and mood, hyperarousal and reactivity, and avoidance that do not remit for at least 1 month subsequent to exposure to a traumatic event (Koenen 2017). Individuals with PTSD may experience a substantial reduction in quality of life and relationships, and the disability resulting from PTSD can have further negative consequences such as obesity, hypertension, comorbid mental health conditions, and suicidality (Scott 2008,

Kibler 2009, Dorrington 2014, Tarrier 2004). Approximately half of people diagnosed with PTSD also have a diagnosis of major depressive disorder (MDD) across a range of populations with varying health and social histories (Flory 2015, Kessler 1995, Peconga 2020, Stein 2001).

First-line pharmacological treatments for PTSD include selective serotonin reuptake inhibitor (SSRI) medications, and psychotherapy (Arroll 2005, Cipriani 2018, Forbes 2010, Hoskins 2015, Kato 2018, Stein 2006). Unfortunately, many people suffering from PTSD either do not receive treatment or are not responsive to existing treatments (Stein 2006). Thus, there is a substantial treatment gap for patients with PTSD, which creates considerable burdens on patients and healthcare systems.

2.2 Study Rationale

Methylone is a new and potentially effective treatment option for participants with PTSD. This consideration is based on:

1. Nonclinical data supporting the efficacy of methylone in animal models of depression, anxiety, and PTSD (Schmidt-Warner 2023, Yu 2022)
2. The efficacy and safety demonstrated in the clinical use of methylone in patients with PTSD (Kelmendi 2022) and MDD (see Section 2.4.3).
3. The similarity of the physiological responses to methylone and MDMA (see Sections 2.4.2 and 2.4.2) with the knowledge that MDMA has demonstrated effectiveness for PTSD (Mitchell 2021).
4. The belief that the reduced intensity of those physiological responses to methylone will improve tolerability compared with MDMA.

The purpose of this two-part study is to evaluate the safety, tolerability, and efficacy of four boosted administrations of methylone (two administrations per dose session) at weekly intervals in participants with PTSD. This study will be open-label, non-controlled in Part A and double-blind, placebo-controlled in Part B. Part A of the study will enroll approximately 15 evaluable participants with PTSD and will provide early safety and efficacy information as well as confirm study procedures for the study. Part B is identical to Part A with the exception of the addition of a placebo arm. Findings from this study will be used to design and power future clinical trials to further evaluate the efficacy and safety of methylone for the treatment of PTSD.

2.3 Nonclinical Development

Methylone has been assessed in multiple GLP and non-GLP primary pharmacology, safety pharmacology, pharmacokinetic (PK) and toxicology nonclinical studies. These are described in detail in the Investigator Brochure (IB); key safety findings are summarized below:

- Methylone demonstrates fast-acting, robust, long-lasting anxiolytic and antidepressant-like activity (Warner-Schmidt 2023).
- Methylone readily crosses the blood-brain barrier and exhibits rapid kinetics, with peak blood concentrations (C_{max}) occurring 1.5 hours after oral administration. Methylone plasma concentrations increased in a dose-related manner; the half-life of methylone in rats is relatively short (approximately 3 hours).
- Mild hyperthermia has been observed in pharmacological safety studies (maximum +1.2°C at 15 mg/kg/day in beagle dogs and +1.2°C at 50 mg/kg/day in Sprague-Dawley rats).

- Methylone does not inhibit any cardiac ion channels (Cardiac Profiler Panel, Eurofins), or bind to serotonin receptor 5HT2B, a known cardiotoxic signal.
- Methylone hydrochloride-related respiratory effects included dose-dependent increases in respiratory rate and minute volume, and concomitant decreases in tidal volume. Respiratory effects were noted for all dose groups and did not resolve by the end of monitoring (≥6 hours post-dose). These results are consistent with the known pharmacology of methylone and other psychomotor stimulants.
- Notable effects were observed on all measured neurobehavioral domains (activity, autonomic, excitability, neuromuscular, physiological, and sensorimotor) in some animals following 25, 50, and 75 mg/kg doses in Sprague-Dawley rats. These effects generally peaked by 1.5 to 6 hours post-dose and resolved by 24 hours post-dose. In the highest dose group, effects arousal and decreased pupil response persisted past 24 hours.
- Repeat dose toxicology study in dogs noted no abnormal coagulation, clinical chemistry, or urinalysis. There was no indication of damage to the renal or hepatic systems following repeated dosing with methylone

2.4 Clinical Development

Methylone is currently in development for post-traumatic stress disorder (PTSD) and other neuropsychiatric conditions. To date methylone has been formally evaluated in 51 participants, including 14 PTSD patients from Part A of this study (Jones 2023) and 37 healthy volunteers from two completed pharmacokinetic studies (Poyatos 2021, Poyatos 2022, Poyatos 2023). Additionally, two case series in 21 patients with PTSD (Kelmendi 2022) and 7 patients with MDD (Averill 2023) have been published. These case series supported the initial therapeutic rationale of methylone for neuropsychiatric conditions.

2.4.1 Completed Open-Label Study in PTSD Patients (IMPACT-1 Part A)

Part A of this study, completed in the UK, was an open-label evaluation of the safety, tolerability, and efficacy of methylone in adults with severe PTSD (CAPS-5 ≥ 35; Jones 2023). Fourteen (14) participants were treated with open-label methylone once a week for 4 weeks (4 treatment sessions). During each treatment session, participants received an initial dose of 150 mg methylone followed by 100 mg of methylone 90 minutes later. Following the Treatment Period, participants entered a 6-week Follow-up Period and a final study visit at Week 10. The primary efficacy endpoint was the change from baseline on the CAPS-5 total severity score. At baseline, the mean CAPS-5 score was 47.8 points.

Treatment with methylone resulted in rapid, robust, and durable effects. Two days after the first dose, the mean change from baseline in CAPS-5 total severity score was -8.4 points. Methylone provided robust improvements in CAPS-5, with the mean change from baseline of -23.3 points two days after the 2nd dose. Six weeks after the last dose, the results were durable with a mean change from baseline in CAPS-5 of -36.2 points. Remission, defined as a CAPS-5 total score of ≤11, was achieved by the majority of participants (61.5%) at the end of study.

Methylone was well tolerated with no drug-related or treatment-emergent serious adverse events to date. The most commonly occurring treatment-emergent adverse event (TEAE) was headache (43%), followed by decreased appetite (29%), non-cardiac chest pain (21%), and fatigue (21%). One unrelated serious adverse event of 'victim of assault' occurred after a participant was off study drug for 20 days.

2.4.2 Completed Healthy Volunteer Studies

Two healthy volunteer studies have been conducted evaluating the safety, pharmacokinetics (PK), and physiological effects of methylone.

Open Label Comparison of Methylone and MDMA Study

An open-label study was conducted to evaluate the acute subjective and physiological effects of methylone compared to MDMA after oral administration (Poyatos 2021). The study enrolled 14 participants who received single oral doses of methylone (n=8) ranging from 100 mg to 300 mg (mean: 187.5 mg) or MDMA (n=6) ranging from 75 mg to 100 mg (mean: 87.5 mg). Subjects were assessed for vital signs, subjective effects using visual analogue scales (VAS), the Addiction Research Centre Inventory (ARCI) short form, and the Evaluation of the Subjective Effects of Substances with Abuse Potential (VESSPA-SSE) questionnaire, and oral fluid concentrations.

Acute pharmacological effects produced by methylone followed the prototypical psychostimulant and empathogenic profile associated with MDMA, although they were less intense. Oral fluid concentrations of MDMA and methylone peaked at 2 hours and rapidly decreased by 4 hrs.

Methylone, at doses up to 300 mg, was well tolerated without safety concerns. Both methylone and MDMA were well-tolerated, and no SAEs were reported. Subjects mentioned slight feelings of dizziness and headache after methylone and MDMA administration. MDMA produced a higher mean increase in both systolic (SBP) and diastolic (DBP), whereas methylone produced a slightly higher mean increase in heart rate, with similar time to maximal effect for both compounds.

Randomized Double-Blind Phase 1 Clinical Trial

A randomized, double-blind, placebo-controlled, cross-over study was conducted to evaluate the pharmacokinetic and subjective experiences in 29 healthy volunteers. The study was divided into two parts, a methylone dose-range finding study in 12 healthy volunteers (Poyatos 2022), followed by a study evaluating 200 mg methylone, 100 mg MDMA, and placebo in 17 healthy volunteers (Poyatos 2023).

Each subject participated in a total of 3 sessions during which they received single oral doses of study drug, each separated by 1 week. In Part 1 three methylone dose escalation cohorts and one MDMA comparison was evaluated:

- 50 mg, 100 mg, placebo (n=3)
- 100 mg, 150 mg, placebo (n=2)
- 150 mg, 200 mg, placebo (n=4)
- 200 mg methylone, 100 mg MDMA, placebo (n=3)

In Part 2, all subjects (n=17) received 200 mg methylone, 100 mg MDMA, and placebo. A 200 mg methylone dose was selected because it evoked similar subjective effects to the 100 mg dose of MDMA (e.g., high or liking feelings), which is considered a common dose used in previous pharmacological studies.

Study outcome measures included pharmacokinetic parameters of methylone, MDMA, and their respective metabolites, subjective effects (eg, VAS, ACRI, and VESSPA), physiological effects (SBP, DPB, HR), and drug tolerability. All subjects completed the methylone doses without any serious adverse effects, including psychiatric symptoms, psychotic episodes, or hallucinations.

Subjective Effects

Subjective effects of methylone on VAS subscales related to euphoria (stimulated, high, good effects, liking, feeling close to others), ARCI MBG subscale (euphoria), and the VESSPA SOC (pleasure and sociability) subscale, displayed dose-dependent increases in effects with higher doses. All methylone doses ≥100 mg were significantly different from placebo.

Changes in subjective effects measured using VAS and questionnaires were comparable between methylone and MDMA. The magnitude of the peak effects was similar between active drug treatments, however, methylone typically reached maximal response earlier (0.5 h vs 0.75 h) and resolved more quickly compared to MDMA (2.5-3 h vs 4 h). Changes in VAS were mainly related to stimulation and well-being, altered perception, and empathy.

Methylone was well tolerated, with no serious adverse events at doses ranging from 50 mg to 200 mg. A total of 3 subjects (21.4%) treated with methylone experienced a total of 5 treatment-emergent adverse events (TEAE). No TEAE term occurred in more than 1 subject. None of the TEAEs were considered to be related to methylone administration.

Dose-dependent increases in blood pressure and heart rate occurred after treatment with methylone. The maximum mean increase in systolic blood pressure was 25.2 mmHg at 0.75 h following administration of 200 mg methylone. Blood pressure increases were transient and returned to baseline values within approximately 3 hours at all doses. Temperature was relatively unchanged following methylone administration.

Overall, both methylone and MDMA administration resulted in significant physiological, subjective, and psychomotor effects compared to placebo. However, these effects occurred earlier and dissipated more quickly following methylone administration compared to MDMA administration. These results indicate that the pharmacological effects of methylone and MDMA occur before maximal plasma concentrations.

2.4.3 Retrospective Case Series

PTSD Patients

In a clinical case series of 21 patients with severe PTSD, treatment with methylone produced acute and enduring improvements in PTSD symptoms, without any notable lasting adverse effects.

Starting doses of methylone were between 100 mg and 270 mg. In 19 of 21 cases, an additional "booster" dose of methylone was administered 1 h after the initial dose to extend the therapeutic window and optimize clinical response.

All patients achieved at least minimal improvement on the clinical global impression of improvement (CGI-I score of 1, 2 or 3) following treatment, with 17 achieving "much" or "very much improved" ratings. This trend was observed even for patients who received only a single dose of methylone (n=9), where 8 patients (89%) achieved CGI-I scores of 1 or 2. For patients with multiple methylone dosing sessions (n=12), initial improvement was noted after the first session in 83% (n=10) of patients. Methylone was well tolerated, and no patients discontinued treatment due to adverse events.

These results suggest that methylone has the potential to be a safe, rapid, and effective treatment for PTSD.

Major Depressive Disorder (MDD) Patients

In a clinical case series of 7 patients with MDD, treatment with methylone produced acute and enduring improvements in MDD symptoms, without any notable lasting adverse effects.

Following methylone dosing session(s), which included single methylone dosing sessions in 2 of the 7 MDD patients, a CGI-I score of 1 or 2, corresponding to “much improved” or “very much improved” was achieved by all participants.

Four of the 7 case narratives included information on durable response, of greater than 5 years in 1 patient, greater than 2 years in 2 patients, and of unknown duration in 1 patient.

3 OBJECTIVES AND ENDPOINTS

This is a two-part study with Part A focusing on safety as the primary objective and Part B focusing on efficacy as the primary objective. Therefore, each Part of the study has separate objectives and endpoints as presented in the tables below.

Table 1: Objectives and Endpoints for Part A

Objective	Endpoints
**Primary**	
To assess the safety and tolerability of oral methylone administered weekly over 4 weeks in participants with PTSD.	• Incidence and severity of TEAEs.<br>• Incidence and severity of AESIs.<br>• Change in HR, SBP, DBP and temperature.<br>• Clinically significant changes in ECG.<br>• Changes from baseline in clinical laboratory parameters (clinical chemistry, hematology and urinalysis).
**Secondary**	
To assess the efficacy of methylone in treating PTSD symptoms.	Mean change from baseline to Week 10 in CAPS-5 (total severity score assessed over 1 week)<br><br>Percentage of participants having:<br>• Treatment response, defined as a<br>○ ≥ 10 point reduction on the CAPS-5 from baseline<br>○ 30% improvement from baseline on CAPS-5<br>○ 50% improvement from baseline on CAPS-5<br>• Remission, defined as a score of ≤ 11 on the CAPS-5
To assess the effect of methylone on sleep quality, functional disability, treatment satisfaction, quality of life, and physical function in participants with PTSD.	Mean change from baseline in the following scales:<br>• CGI-S<br>• MADRS<br>• SDS<br>• PCL-5<br>• PGI-S<br>• BDI-II<br>• WEMWBS<br>• PSQI<br><br>Percentage of participants with improvement on the following scales:<br>• PGI-C<br>• CGI-I
[redacted]	
[redacted]	• [redacted]<br>• PTGI

Abbreviations: [redacted] AE = adverse event; AESI = adverse event of special interest; BDI-II = Beck Depression Inventory-II; DBP = diastolic blood pressure; CAPS-5 = Clinician-Administered PTSD Scale for DSM-5; CGI-I = Clinician Global Impression of Improvement; CGI-S = Clinical Global Impression of Severity; C-SSRS = Columbia Suicide Severity Rating Scale; DBP = diastolic blood pressure; ECG = electrocardiogram; HR = heart rate; LEC-5 = Life Events Checklist for DSM-5; MADRS = Montgomery-Åsberg Depression Rating Scale; [redacted]

[redacted] MINI = Mini International Neuropsychiatric Interview; PCL-5 = PTSD Checklist for DSM-5; PGI-C = Patient Global Impression of Change; PGI-S = Patient Global Impression of Severity; PSQI = Pittsburgh Sleep Quality Index; PTGI = Post-traumatic Growth Inventory; PTSD = Post-traumatic stress disorder; SDS = Sheehan Disability Scale; SBP = systolic blood pressure; TEAEs = treatment-emergent adverse events; WEMWBS = Warwick-Edinburgh Mental Wellbeing Scale.

Table 2: Objectives and Endpoints for Part B

Objectives	Endpoints
**Primary**	
To assess the efficacy of methylone in treating PTSD symptoms.	Mean change from baseline to Week 10 compared with placebo in CAPS-5 total severity score.
**Secondary**	
To assess the effect of methylone compared to placebo on sleep quality, functional disability, treatment satisfaction, quality of life, and physical function in participants with PTSD.	Mean change from baseline compared with placebo in the following scales:<br>• CGI-S<br>• MADRS<br>• SDS<br>• PCL-5<br>• PGI-S<br>• BDI-II<br>• WEMWBS<br>• PSQI<br><br>Percentage of participants with:<br>• Treatment response, defined as a<br>○ ≥ 10 point reduction on the CAPS-5 from baseline<br>○ 30% improvement from baseline on CAPS-5<br>○ 50% improvement from baseline on CAPS-5<br>• Remission, defined as a score of ≤ 11 on the CAPS-5<br>• Improvement on the PGI-C<br>• Improvement on the CGI-I
To assess the safety and tolerability of oral methylone compared to placebo administered weekly over 4 weeks in participants with PTSD.	• Incidence and severity of TEAEs<br>• Incidence and severity of AESIs<br>• Change in HR, SBP, DBP and temperature during each dosing session<br>• Clinically significant changes in ECG<br>• Changes from baseline in clinical laboratory parameters (clinical chemistry, hematology, and urinalysis)
[redacted]	
[redacted]	Effect of the following scales, compared with placebo:<br>• PTGI<br>• [redacted]

Abbreviations: [redacted] AE = adverse event; AESI = adverse event of special interest; BDI-II = Beck Depression Inventory-II; DBP = diastolic blood pressure; CAPS-5 = Clinician-Administered PTSD Scale for DSM-5; CGI-I = Clinician Global Impression of Improvement; CGI-S = Clinical Global Impression of Severity; C-SSRS = Columbia Suicide Severity Rating Scale; DBP = diastolic blood pressure; ECG = electrocardiogram; HR = heart rate; LEC-5 = Life Events Checklist for DSM-5; MADRS = Montgomery-Åsberg Depression Rating Scale; [redacted] MINI = Mini International Neuropsychiatric Interview; PCL-5 = PTSD

Checklist for DSM-5; PGI-C = Patient Global Impression of Change; PGI-S = Patient Global Impression of Severity; PSQI = Pittsburgh Sleep Quality Index; PTGI = Post-traumatic Growth Inventory; PTSD = Post-traumatic stress disorder; SDS = Sheehan Disability Scale; SBP = systolic blood pressure; TEAEs = treatment-emergent adverse events; WEMWBS = Warwick-Edinburgh Mental Wellbeing Scale.

4 STUDY DESIGN

4.1 Study Design Overview

This is a two-part study to assess methylone for the management of the symptoms of PTSD. Part A is an open-label, non-controlled assessment in approximately 15 evaluable participants with PTSD to provide early safety and efficacy information and to confirm procedures for the study. After completion of Part A, Part B will begin and enroll approximately 64 participants. Part B is identical to Part A with the exception of the inclusion of a placebo arm in Part B.

The open-label treatment in Part A is:

- Methylone 150 mg, with a booster administration of 100 mg administered 90 (±10) minutes after the initial administration, during each dose session.

Part B is a randomized, double-blind, parallel-group, placebo-controlled portion of the study designed to assess the utility of methylone for the management of the symptoms of PTSD. This is a multi-center global study that will be conducted in up to approximately 25 sites.

Planned enrollment includes approximately 79 evaluable participants (approximately 15 in Part A and approximately 64 in Part B). There are 4 planned dose sessions for each participant at weekly intervals. During each dose session, participants will receive two administrations of study drug (initial dose plus a booster dose). Participants in Part B will be randomized 1:1 to receive either methylone or placebo at each of the weekly dose sessions for the duration of the study.

The first 8 participants from Part A who have the opportunity to complete the Day 29 Follow-Up visit are considered the Safety Run-in participants. The DSMB will be convened to review the safety data of these participants. The DSMB will subsequently be convened to review the safety data after all participants in Part A have the opportunity to complete the Day 29 Follow-Up visit.

The two blinded study treatment arms for Part B are:

- Methylone, an initial administration of 150 mg, followed by a booster administration of 100 mg 90 (±10) minutes after the initial administration, during each dose session.
- Matched placebo at each timepoint (initial + booster) during each dose session.

For Part B, DSMB meetings will occur at intervals of approximately 20 participants completing the Day 29 Follow Up Visit or EOS if the participant withdraws early.

For each participant in Parts A and B, the study will consist of:

- **Screening Period:** Informed consent, eligibility assessment, and enrollment of eligible participants.
- **Baseline/Preparatory Session:** Baseline assessments, confirmation of eligibility, and a preparatory psychoeducation session leading to enrollment confirmation.
- **Treatment Period:** Four weekly dose sessions, with associated remote sessions. The dose sessions will last at least 8 hours, and until all effects (physical and psychological) have resolved (whichever is longer). Each dose session will be followed by a safety phone call 1 day after dosing and efficacy assessments 2 days after dosing. Each dosing period may be recorded for quality and training purposes. The recordings will be reviewed to ensure the dosing session monitor is adhering to the dosing session monitor training and for training purposes.

- **Follow-Up Period:** Follow-Up visits for efficacy and safety will occur at 1, 2, 3 and 6 weeks post-final study drug administration. In addition, there will be Reflection Sessions 1, 2 and 3 weeks post-final study drug administration. Participants will be contacted via telephone on Day 57 (±2 days) for ensuring continuity.

4.2 Study Visit Breakdown

**Screening Period**		
**Study Visit**	**Visit Timing**	**Brief Description of Events**
**Screening (Visit 1)**	Day -28 to Baseline	Obtain informed consent and assess all Screening measures including Clinician Administered PTSD Scale for DSM-5 (CAPS-5; past month version), MINI 7.0.2, PCL-5, LEC-5, SPQ (and SCID-5-PD, if needed) C-SSRS, demographics and medical history, substance use history and pre-study medications.<br>The CAPS-5, MINI, and SCID-5-PD (if needed) will be administered by the Central rater and recorded for rater fidelity.<br>Physical examinations (including height and body weight), vital signs, temperature, 12-lead ECG, and labs (including safety labs, urine drugs test, and pregnancy test [women of childbearing potential (WOCBP) only, see Section 14.4], plus serum follicle stimulating hormone [FSH] test [if needed, see Section 14.4]) and a review of eligibility criteria will also be performed.

**Baseline/Preparatory Session**		
**Study Visit**	**Visit Timing**	**Brief Description of Events**
**Baseline/ Preparatory Session (Visit 2)**	Day -4 to Day -1	**Baseline:** The following safety assessments will be assessed prior to evaluations by the central rater to ensure the participant continues to meet eligibility requirements: urine drugs test, vital signs, concomitant medication review, and C-SSRS.<br>Central rater scales will be administered via telephone/video; the CAPS-5 will be performed first followed by MADRS and CGI-S. The CAPS-5, MADRS, and CGI-S will be recorded for rater fidelity.<br><br>This will be followed by the participant completing the self-administered psychometric assessments (PCL-5, BDI-II, SDS, WEMWBS, and PGI-S) as well as the PSQI and PTGI. The order of completion should be: Central rating scales and then the participant reported scales.
		**Preparatory Session:** The following session is to be performed after all baseline assessments have been performed:<br>• Psychoeducation session, conducted by a trained dosing session monitor. The dosing session monitor will proactively provide the participant with an understanding of the anticipated experience following administration of an entactogen medication, like methylone.<br>If needed, a second preparatory session may occur prior to first dosing to ensure the participant is adequately prepared for the treatment period.

**Treatment Period**		
**Study Visit**	**Visit Timing**	**Brief Description of Events**
**Dose Session 1 (Visit 3 –** Within 4 days of Baseline/ Preparatory Session	Day 1 – Predose	Prior to enrollment (Part A) or randomization (Part B), an eligibility criteria review, a negative urine pregnancy test (WOCBP only), and urine drug screen will be required. Once confirmed that the participant meets all criteria, they will be enrolled (Part A) or randomized (Part B) onto the study. Predose assessments will then be performed (including vital signs, temperature, 12-lead ECGs, and C-SSRS).<br>Participants should have fasted (water only) for at least 2 hours prior to dosing and for at least 2 hours after the booster dose.<br>(Methyl) xanthine- and caffeine-containing products should be avoided for 2 hours predose until at least 8 hours after the initial dose.
	Day 1 – Dose Session 1 (~8 hours)	Recording begins immediately prior to dosing.<br>Following dosing, adverse event monitoring begins.<br>The open-label treatment for Part A is:<br>• Methylone 150 mg, with a booster administration of 100 mg administered 90 (±10) minutes later.<br><br>The two blinded study treatment arms for Part B are:<br>• Methylone 150 mg, with a booster administration of 100 mg administered 90 (±10) minutes later.<br>• Matched placebo (initial and booster).<br><br>Safety will be assessed at the following timepoints during the dose session:<br>• BP and HR will be recorded 10 minutes predose and at 30-minute intervals (±15 minutes) post-administration until the end of the dose session<br>○ In addition, BP and HR will be recorded within 15 minutes (+5 minutes) prior to administration of the booster dose (equivalent of 90-minute timepoint)<br>• Temperature will be recorded predose and at 60-minute intervals (±15 minutes) post-initial dose-administration, until end of the dose session<br>• 12-lead ECG will be performed predose and at the end of the dosing session
	Day 1 – Discharge	[redacted]<br>Prior to discharge, the dosing session monitor will encourage the participant to reflect on their experience.
**Telephone Call (Visit 4)**	Day 2	Participants will be contacted remotely to ensure psychological safety and for AE collection and concomitant medication monitoring and completion of the C-SSRS.
**Efficacy Assessments (Visit 5)**	Day 3 (+1 day)	Central rater scales will be administered via telephone/video; the CAPS-5 (past week version) will be performed first, followed by MADRS, CGI-S, and CGI-I. The CAPS-5, MADRS, CGI-S, and CGI-I will be recorded for rater fidelity.<br><br>This will be followed by the participant completing the self-administered psychometric assessments (PCL-5, SDS, PGI-S, and PGI-C). The order of completion should be: Central rating scales and then the participant reported scales.
**Dose Session 2 (Visit 6)**	Day 8 (±1 day) – Predose	Predose assessments will be performed (including urine drugs test urine pregnancy test [WOCBP only], vital signs, temperature, 12-lead ECGs, and C-SSRS).

**Treatment Period**		
**Study Visit**	**Visit Timing**	**Brief Description of Events**
		Participants should have fasted (water only) for at least 2 hours prior to dosing and for at least 2 hours after the booster dose.<br>(Methyl) xanthine- and caffeine-containing products should be avoided for 2 hours predose until at least 8 hours after the initial administration.
	Day 8 (±1 day) – Dose Session 2 (~8 hours)	Recording begins immediately prior to dosing.<br>The open-label treatment for Part A is:<br>• Methylone 150 mg, with a booster administration of 100 mg administered 90 (±10) minutes later.<br><br>The two blinded study treatment arms for Part B are:<br>• Methylone 150 mg, with a booster administration of 100 mg administered 90 (±10) minutes later.<br>• Matched placebo (initial and booster).<br><br>Safety will be assessed at the following timepoints during the dose session:<br>• BP and HR will be recorded 10 minutes predose and at 30-minute intervals (±15 minutes) post-administration until the end of the dose session<br>○ In addition, BP and HR will be recorded within 15 minutes (+5 minutes) before administration of the booster dose (equivalent of 90-minute timepoint)<br>• Temperature will be recorded predose and at 60-minute intervals (±15 minutes) post-initial dose-administration, until end of the dose session<br>• 12-lead ECG will be performed predose and at the end of the dosing session
	Day 8 (±1 day) – Discharge	[redacted]<br>Prior to discharge, the dosing session monitor will encourage the participant to reflect on their experience.
**Telephone Call (Visit 7)**	Day 9	Participants will be contacted remotely to ensure psychological safety and for AE collection and concomitant medication monitoring and completion of the C-SSRS.
**Efficacy Assessments (Visit 8)**	Day 10 (+1 day)	Central rater scales will be administered via telephone/video; the CAPS-5 (past week version) will be performed first, followed by MADRS, CGI-S, and CGI-I. The CAPS-5, MADRS, CGI-S, and CGI-I will be recorded for rater fidelity.<br><br>This will be followed by the participant completing the self-administered psychometric assessments (PCL-5, SDS, PGI-S, and PGI-C). The order of completion should be: Central rating scales and then the participant reported scales.
**Dose Session 3 (Visit 9)**	Day 15 (±1 day) – Predose	Predose assessments will be performed (including urine drugs test, urine pregnancy test [WOCBP only], vital signs, temperature, 12-lead ECGs, safety labs and C-SSRS).<br>Participants should have fasted (water only) for at least 2 hours prior to dosing and for at least 2 hours after the booster dose.<br>(Methyl) xanthine- and caffeine-containing products should be avoided for 2 hours predose until at least 8 hours after the initial administration.
	Day 15 (±1 day) –	Recording begins immediately prior to dosing.<br>The open-label treatment for Part A is:

Treatment Period		
**Study Visit**	**Visit Timing**	**Brief Description of Events**
	Dose Session 3 (~8 hours)	• Methylone 150 mg, with a booster administration of 100 mg administered 90 (±10) minutes later.<br><br>The two blinded study treatment arms for Part B are:<br>• Methylone 150 mg, with a booster administration of 100 mg administered 90 (±10) minutes later.<br>• Matched placebo (initial and booster).<br><br>Safety will be assessed at the following timepoints during the dose session:<br>• BP and HR will be recorded 10 minutes predose and at 30-minute intervals (±15 minutes) post-administration until the end of the dose session.<br>○ In addition, BP and HR will be recorded within 15 minutes (+5 minutes) before administration of the booster dose (equivalent of 90-minute timepoint)<br>• Temperature will be recorded predose and at 60-minute intervals (±15 minutes) post-initial dose-administration, until end of the dose session.<br>• 12-Lead ECG will be performed predose and at the end of the dosing session.
	Day 15 (±1 day) – Discharge	[redacted]<br>Prior to discharge, the dosing session monitor will encourage the participant to reflect on their experience.
**Telephone Call (Visit 10)**	Day 16	Participants will be contacted remotely to ensure psychological safety and for AE collection and concomitant medication monitoring and completion of the C-SSRS.
**Efficacy Assessments (Visit 11)**	Day 17 (+1 day)	Central rater scales will be administered via telephone/video; the CAPS-5 (past week version) will be performed first, followed by other questionnaires. The CAPS-5, MADRS, CGI-S, and CGI-I will be recorded for rater fidelity.<br>This will be followed by the participant completing the self-administered psychometric assessments (PCL-5, BDI-II, SDS, WEMWBS, PGI-S, and PGI-C). The order of completion should be: Central rating scales and then the participant reported scales.
**Dose Session 4 (Visit 12)**	Day 22 (±1 day) – Predose	Predose assessments will be performed (including urine drugs, urine pregnancy test [WOCBP only], vital signs, temperature, 12-lead ECGs, and C-SSRS).<br>Participants should have fasted (water only) for at least 2 hours prior to dosing and for at least 2 hours after the booster dose.<br>(Methyl) xanthine- and caffeine-containing products should be avoided for 2 hours predose until at least 8 hours after the initial administration.
	Day 22 (±1 day) – Dose Session 4 (~8 hours)	Recording begins immediately prior to dosing.<br>The open-label treatment for Part A is:<br>• Methylone 150 mg, with a booster administration of 100 mg administered 90 (±10) minutes later.<br><br>The two blinded study treatment arms for Part B are:<br>• Methylone 150 mg, with a booster administration of 100 mg administered 90 (±10) minutes later.<br>• Matched placebo (initial and booster).

**Treatment Period**		
**Study Visit**	**Visit Timing**	**Brief Description of Events**
		Safety will be assessed at the following timepoints during the dose session:<br>• BP and HR will be recorded 10 minutes predose and at 30-minute intervals (±15 minutes) post-administration until the end of the dose session<br>◦ In addition, BP and HR will be recorded within 15 minutes (+5 minutes) before administration of the booster dose (equivalent of 90-minute timepoint)<br>• Temperature will be recorded predose and at 60-minute intervals (±15 minutes) post-initial dose-administration, until end of the dose session<br>• 12-Lead ECG will be performed predose and at the end of the dosing session.
	Day 22 (±1 day) – Discharge	[redacted]<br>Prior to discharge, the dosing session monitor will encourage the participant to reflect on their experience.
**Telephone Call (Visit 13)**	Day 23	Participants will be contacted remotely to ensure psychological safety and for AE collection and concomitant medication monitoring and completion of the C-SSRS.
**Efficacy Assessments (Visit 14)**	Day 24 (+1 day)	The participant will attend the unit to complete psychometric assessments.<br>Central rater questionnaires will be administered via telephone/video; the CAPS-5 (past week version) will be performed first, followed by MADRS, CGI-S, and CGI-I. The CAPS-5, MADRS, CGI-S, and CGI-I will be recorded for rater fidelity.<br>This will be followed by the participant completing the self-administered psychometric assessments (PCL-5, SDS, PGI-S, and PGI-C). The order of completion should be: Central rating scales and then the participant reported scales.

**Follow-Up Period**		
**Study Visit**	**Visit Timing**	**Brief Description of Events**
**Follow-Up Visit (Visit 15)**	Day 29 (±1 day) – Follow-Up Visit	The CAPS-5 (past week version), MADRS, CGI-S, and CGI-I will be performed (via telephone/video) by a central rater. The CAPS-5, MADRS, CGI-S, and CGI-I will be recorded for rater fidelity.<br>Following the observer-administered scales, self-administered psychometric assessments will then be performed (PCL-5, BDI-II, SDS, WEMWBS, PGI-S, and PGI-C), as well as the PSQI, PTGI, and BIQ (Part B only).<br>Safety will be assessed by the following:<br>• Adverse event collection and concomitant medication monitoring, C-SSRS, 12-lead ECG, safety labs and urine pregnancy test [WOCBP only]).
	Day 29 (±1 day) – Reflection Session	A reflection session (led by the dosing session monitor) assessing the participant's experience will also be performed.
**Follow-Up Visit (Visit 16)**	Day 36 (±2 days) – Follow-Up Visit	The CAPS-5 (past week version), MADRS, CGI-S, and CGI-I will be performed (via telephone/video) by a central rater. Following the observer-administered scales, self-administered psychometric assessments will then be performed (PCL-5, SDS, PGI-S, and PGI-C).<br>Adverse event collection, concomitant medication monitoring and the C-SSRS will be performed.

Follow-Up Period		
**Study Visit**	**Visit Timing**	**Brief Description of Events**
	Day 36 (±2 days) – Reflection Session	A reflection session (led by the dosing session monitor) assessing the participant's experience will also be performed.
**Follow-Up Visit (Visit 17)**	Day 43 (±2 days) – Follow-Up Visit	The CAPS-5 (past week version), MADRS, CGI-S, and CGI-I will be performed (via telephone/video) by a central rater.<br>Following the observer-administered scales, self-administered psychometric assessments will then be performed (PCL-5, BDI-II, SDS, WEMWBS, PGI-S, and PGI-C). The order of completion should be: Central rating scales and then the participant reported scales.<br><br>Adverse event collection, concomitant medication monitoring and the C-SSRS will be performed.
	Day 43 (±2 days) – Reflection Session	A reflection session (led by the dosing session monitor) assessing the participant's experience will also be performed.
**Telephone Call (Visit 18)**	Day 57 (±2 days)	Participants will be contacted remotely for AE collection, concomitant medication monitoring, and completion of the C-SSRS.
**EOS Visit (Visit 19)**	Day 64 (±3 days)	The CAPS-5 (past week version), MADRS, CGI-S, and CGI-I will be performed (via telephone/video) by a central rater.<br>Following the observer administered scales, self-administered psychometric assessments will then be performed (PCL-5, BDI-II, SDS, WEMWBS, PGI-S, and PGI-C), as well as the PSQI and PTGI. The order of completion should be: Central rating scales and then the participant reported scales.<br>Adverse event collection, concomitant medication monitoring, physical examination (including weight), and the C-SSRS will be performed at the EOS visit. Create an Exit Plan for the participant.
**Early Termination**	-	The CAPS-5 (past week version), MADRS, CGI-S, and CGI-I will be performed first (via telephone/video) by a central rater. Following the observer-administered scales. self-administered psychometric assessments will then be performed (PCL-5, BDI-II, SDS, WEMWBS, PGI-S, and PGI-C), as well as the PSQI, and PTGI. The order of completion should be: Central rating scales and then the participant reported scales. The following safety assessments will occur: C-SSRS and AE and concomitant medication monitoring.<br><br>If the participant discontinues prior to Day 29, the following assessments will also occur:<br>• Physical examination, vital signs, adverse event monitoring, 12-lead ECG, safety labs, and urine pregnancy test [WOCBP only]<br>• BIQ (Part B only)

4.3 Planned Duration of Study

The expected duration of participation for each participant who completes all study visits, from Screening to the EOS visit, is up to 16 weeks. The treatment duration for both Part A and Part B of the study are expected to be the same, as procedures are identical.

- Screening may take up to 28 days.

- The Baseline/Preparatory Period could last up to 4 days and should not be performed >4 days prior to Day 1.
- Enrollment confirmation will take place prior to dosing on Day 1; once enrollment has been confirmed, the Treatment Period will commence.
- The 4-week Treatment Period will consist of four dose sessions 1 week apart, with a telephone visit 1 day after each dose session for collection of safety assessments (AEs, concomitant medications, and C-SSRS) and a follow-up visit to assess safety and efficacy 2 days after each dose session.
- After the final session of the Treatment Period (Visit 14), participants will enter the Follow-Up period. Participants will attend a Follow-Up visit and a Reflection Session approximately 1, 2 and 3 weeks after the final study drug administration, followed by an EOS visit 6 weeks after the final study drug administration. Participants will also be contacted via telephone for collection of safety assessments on Day 57 (±2 days).
- Participants withdrawing before completion of the study should be encouraged to attend a visit that would duplicate the EOS visit to collect safety and efficacy information.

4.4 Rationale for Study Design

This two-part study is designed to assess the safety and efficacy of four dose sessions of methylone over 4 weeks in participants with PTSD.

Part A will be an open-label, non-controlled assessment to confirm procedures to be used in Part B; with the exception of inclusion of a placebo-control in Part B, procedures will be identical. Double-blinding with use of placebo is included in Part B of the study to permit a true assessment of the safety and tolerability of methylone and to evaluate the benefits and risks of methylone. This design also allows for an objective assessment of key safety issues such as suicidality, which can be part of the disease process (PTSD) rather than a side effect of treatment.

Also included are standard assessments to evaluate the safety and tolerability, such as vital signs, physical examination, 12-lead ECGs, clinical laboratory evaluations and AE collection.

The battery of clinical outcome assessments to be performed are those routinely used to assess the severity of PTSD symptoms and functional impairment. These include observer-blinded assessments of PTSD severity and self-administered questionnaires that explore the participant's experience of life events. These questionnaires and scales are standardly used in clinical trials investigating psychoactive medications in similar patient populations.

4.5 Dose Rationale

Both efficacy and safety are likely dose-related. [redacted] The relatively short half-life of methylone (translates to duration of effect) recommends the use of an initial dose (150 mg) followed by a booster (100 mg, within an individual dose session) to provide for a longer physiological impact, while reducing the C_{max} (compared with a single dose of 250 mg) which would provide for a safer dosing regimen.

In order to further enhance the likelihood of benefit, it is proposed that each participant should attend four dose sessions.

[redacted]

[redacted]

[redacted] The most common safety findings were increases in blood pressure and heart rate.

[redacted]

The methylone dose level has been selected as it is believed that this dose will maximize the opportunity to observe a clinical effect, but at the same time is anticipated to be well tolerated. To that end, administering methylone over four dose sessions at weekly intervals has been selected. At each dose session, there will be an initial administration of 150 mg followed by a booster administration of 100 mg after 90 minutes (±10 minutes).

- Based on the completed Phase 1 studies and PK modelling, an initial administration of 150 mg would be anticipated to induce meaningful psychological effects that would be extended by the booster administration of 100 mg given 90 minutes later (±10 minutes).
- Based on the retrospective case series, the use of multiple dose sessions would optimize the likelihood of a clinical benefit.
- This dose regimen would provide a total dose during a dose session of 250 mg, but because the dose is split, the C_{max} is anticipated to be lower than if 250 mg was given as a single dose.
  - This initial administration is lower than the maximum single dose given to the healthy volunteers (up to 300 mg) in the open-label Phase 1 study.
  - The proposed dose is significantly lower than the dose given to most patients reported in the Case Series.

Taken together, these data suggest that a dose of 150 mg plus a booster dose of 100 mg administered on four occasions over 4 weeks would provide a physiological response that is believed to predict clinical benefit, and that this regimen would be well tolerated and provide confidence that this proposed study can be conducted safely.

To further ensure the safety of participants, all study drug administration will be under the direct supervision of the Research Site Staff and a dosing session monitor, and a DSMB will regularly review emerging safety data.

4.6 End of Study

4.6.1 End-of-Study Definition

The EOS is defined as the date of the last visit of the last participant in the study.

A participant is considered to have completed the study if the participant has completed all periods of the study including the last visit (Day 64).

4.6.2 Exit Plan

At the conclusion of each participant's participation, they will be provided with an Exit Plan. The Exit Plan summarizes the study treatment, their current medications, and provides them a contact for more information about the study if needed. Participants may request a referral for further therapeutic or medical care if appropriate. Enrolled participants who terminate the study early will be provided an Exit Plan at their last contact. Participants who continue into an extension study are not required to have an Exit Plan.

4.7 Data and Safety Monitoring Board

The objective of the DSMB is to review safety data (Part A and Part B) during the clinical trial.

The DSMB will comprise:

- A psychiatrist, preferably with PTSD experience.
- Cardiologist/Internal medicine specialist.
- A statistician.

Data and Safety Monitoring Board meetings will be scheduled to occur after 8 participants in Part A have completed the Day 29 Follow-Up visit, or EOS if the participant withdraws early

(Safety Run-in Phase) and once after all participants in Part A have the opportunity to complete the Day 29 Follow-Up visit.

For Part B, DSMB meetings will occur at intervals of approximately 20 participants completing the Day 29 Follow Up Visit or EOS if the participant withdraws early. Ad hoc DSMB meetings will occur as needed.

The safety and tolerability data to be reviewed will include, at a minimum, AEs, physical examinations, 12-lead ECGs, vital signs, clinical laboratory evaluation results, and C-SSRS results, summarized by a non-voting Statistician. There are no stopping rules for either efficacy or futility.

Further details on the operation of the DSMB will be defined in a standalone supporting document.

4.8 Criteria For Termination of Dosing for An Individual Participant

Dosing will be terminated for an individual participant if **any** of the following occur:

1) If a participant experiences an intolerable AE.
2) If a participant demonstrates significant non-compliance.
3) If a participant is administered a prohibited medication which, in the opinion of the Investigator and Medical Monitor, has the potential to significantly impact participant safety or data integrity.
4) If a female participant becomes pregnant.
5) If any participant reports Active Suicidal Ideation since last study drug administration, as evidenced by a ‘Yes’ response to the C-SSRS Suicidal Ideation questions 4 or 5.
6) If a participant reports any suicidal behavior since last study drug administration, as evidenced by a ‘Yes’ response to the following suicidal behavior questions on the C-SSRS:
    a) Actual attempt, or
    b) Interrupted attempt, or
    c) Aborted attempt.
7) If a participant experiences Grade 3 Hypertension (as defined in the National Cancer Institute CTCAE [Version 5.0]) during a dosing session:
    a) Grade 3: SBP ≥160 mmHg or DBP ≥100 mmHg, requiring the need for medical intervention with more than one drug or more intensive therapy than previously used.
8) If a participant develops an abnormality of liver function tests (LFTs) that meets the following criteria during the treatment period:
    a) Aspartate aminotransferase (AST) or alanine aminotransferase (ALT):
        i) >5-fold upper limit of normal (ULN), or
        ii) >3-fold ULN with concomitant serum total bilirubin ≥2-fold the ULN with ≥35% direct.
9) If a participant experiences a clinically significant elevation of their temperature by >2.0°C (and ≥38°C) during the dosing session.

10) If a participant experiences a severe psychological event related to dosing that requires an intervention.
11) If a participant experiences a cardiovascular event associated with dosing that requires an intervention.
12) If a participant develops a neurological deficit.
13) If a participant experiences a treatment-related SAE.
    a) Note that an SAE that is considered not treatment-related by the Investigator should not automatically lead to discontinuation of dosing. Instead, that participant and the SAE should be discussed with the Medical Monitor and Sponsor (or delegate) to make a determination as to whether dosing should continue or be terminated.

Any participant who discontinues the study early will complete an Early Termination visit and an Exit Plan will be provided to the participant.

4.9 Criteria for Interruption of Randomization/Dosing of New Participants

If the following occur, enrollment/randomization and dosing of new participants will be interrupted until a DSMB review has indicated that it is safe to continue:

1) If there appears to be an unacceptable tolerability profile based on the nature, frequency and severity of observed AEs and/or clinical safety monitoring.
2) If **two** participants report Active Suicidal Ideation since last study drug administration, as evidenced by a 'Yes' response to the C-SSRS Suicidal Ideation questions 4 or 5.
3) If **two** participants report any suicidal behavior since last study drug administration, as evidence by a 'Yes' response to the following suicidal behavior questions on the C-SSRS:
    a) Actual attempt, or
    b) Interrupted attempt, or
    c) Aborted attempt.
4) If **two** participants experience Grade 3 Hypertension (SBP ≥160 mmHg or DBP ≥100 mmHg), or Grade 2 (SBP ≥140 mmHg or DBP ≥90 mmHg), (as defined in the National Cancer Institute CTCAE [Version 5.0]), persisting over 48 hours post dosing, and require the need for medical intervention with more than one drug or more intensive therapy than previously used.
5) If **two** participants develop an abnormality of LFTs that meets the following criteria during the treatment period:
    a) AST or ALT:
        i) >5-fold ULN, or
        ii) >3-fold ULN with concomitant serum total bilirubin ≥2-fold the ULN with ≥35% direct.
6) If **two** participants experience a clinically significant elevation of their temperature by >2°C (and ≥38°C) during the dosing session.
7) If **two** participants experience a severe psychological event related to dosing that requires an intervention.

8) If **two** participants experience a cardiovascular event associated with dosing that requires an intervention.
9) If **two** participants develop a neurological deficit.
10) If **three** participants experience a treatment-related SAE of any nature.
11) If **two** participants experience the same SAE.

The Investigators and Sponsor have the right to terminate the study or suspend enrollment at any time. The Investigator(s) will be notified by telephone and in writing if the Sponsor decides to suspend or terminate the study for any reason. The written notice will provide the Investigator with the reason that the site and/or study was suspended or terminated, along with instructions on how the site should proceed. Should the Sponsor terminate the study early, all participants will complete an Early Termination visit and an Exit Plan will be provided to the participant.

Reasons for terminating the study may include, but are not limited to, the following:

- Discovery (from this or other studies) of an unexpected, serious or unacceptable health hazard to participants.
- A decision on the part of the Sponsor to suspend or discontinue testing, evaluation or development of the product.
- Evidence from the data that there are sufficient technical problems with the study to believe with a high degree of certainty that participants are being exposed without a realistic expectation of evaluable data.
- Participant enrollment is unsatisfactory.
- Insufficient adherence to Protocol requirements or an unacceptably high rate of missing, erroneous or improperly collected data that threaten the scientific integrity of the study.

Reasons for suspending enrollment at a site may include, but are not limited to, the following:

- Failure of the Investigator to comply with pertinent regulatory regulations.
- Submission of knowingly false information from the research facility to the Sponsor.
- Participant enrollment is unsatisfactory.
- Insufficient adherence to Protocol requirements or an unacceptable high rate of missing, erroneous or improperly collected data that threaten the scientific integrity of the study.

5 STUDY TREATMENTS

5.1 Name and Description of Investigational Product(s)

The study interventions to be administered are detailed in Table 3. Further supportive information will be provided in the Pharmacy Manual.

Table 3: Study Interventions Administered

**Intervention Name**	**Methylone**	**Placebo (Part B)**
**Dose Formulation**	Capsule	Capsule
**Unit Dose Strength**	50 mg methylone capsule	50 mg placebo capsule
**Dosage Level**	Initial Administration: 150 mg (3 × 50 mg methylone capsules) Booster Administration: 100 mg (2 × 50 mg methylone capsules)	Initial Administration: 150 mg (3 × 50 mg placebo capsules) Booster Administration: 100 mg (2 × 50 mg placebo capsules)
**Route of Administration**	Oral	Oral
**Use**	Investigational	Placebo

The Investigational Medicinal Product (IMP) supplied by the Sponsor or delegate is to be used exclusively in the clinical study according to the instructions in this Protocol and the Pharmacy Manual.

The Site Investigator will be responsible for storage and accountability of the IMP taking into account that methylone is a controlled substance (detailed storage/security guidance is provided in the Pharmacy Manual).

5.2 Study Drug Administration

Participants should have fasted (water only) for at least 2 hours prior to initial dosing and for at least 2 hours after the booster dose. Water and clear fluids will be encouraged up to a maximum of 3 liters over the dose session (see Section 5.6).

Study drug (methylone) (or matched placebo for Part B) will be orally administered on Day 1, Day 8 (±1 day), Day 15 (±1 day) and Day 22 (±1 day). An initial administration of 150 mg methylone (or matched placebo for Part B) will be given, followed by a booster administration of 100 mg methylone (or matched placebo for Part B) 90 (±10) minutes later.

5.3 Psychological and Medical Support on Dosing Days

5.3.1 Psychological Support

On the dosing days, participants will be provided unstructured psychological support for at least 8 hours after the dose and until the subjective effects have subsided. The psychological support will be provided by a lead dosing session monitor (in the room) with an assistant monitor reviewing via live remote video monitoring. The lead dosing session monitor must be a licensed healthcare provider with graduate-level professional training and clinical experience in psychotherapy. The lead dosing session monitor must be licensed to practice independently.

Examples of acceptable credentials for the lead dosing session monitor are as follows:

- Clinical or counseling psychologist (PhD or PsyD)
- Psychiatrist or other physician (MD or DO)
- Master of Social Work (MSW)
- Masters Licensed Clinical Professional Counselor (LCPC)

- Licensed Marriage and Family Therapist (LMFT)
- Psychiatric Nurse Practitioner (Psychiatric NP)

Each lead dosing session monitor will undergo formal training prior to working with participants in this study. The assistant monitor must have a bachelor's degree and at least 1 year of clinical experience in a licensed mental healthcare setting.

The clinical sites will make every effort to ensure the same lead dosing session monitor will be present for all sessions with each participant. Their role will be to provide unstructured psychological support as the participant explores their response to the medication. The dosing session monitor will not be involved in any medical, nursing, or other research activities.

During the Preparatory session, the dosing session monitor will provide unstructured psychological guidance. This session will focus on psychoeducation about PTSD, possible physiological and psychological effects of methylone (including possible adverse and challenging effects), building safety for the therapeutic relationship, obtaining the background for the trauma and preparing the participant for dose sessions. Goals and expectations for the dose sessions will also be discussed.

During each dose session, the dosing session monitor will provide non-directive support of psychological well-being continuously during each dose session.

- During the early stages of the dose session, while the acute effects of methylone are active, the dosing session monitor will provide reactive support and engaged listening. The dosing session monitor will provide guidance if the participant falls into a negative pattern.
- At the end of each dose session, the dosing session monitor will encourage the participant to reflect on their experience and on any strong emotions or thoughts they might have encountered.

On Days 29, 36 and 43, post-dosing Reflection Sessions will be performed; the dosing session monitor will help the participant integrate their experiences over the four weeks of treatment, and to draw-out (if necessary) particular aspects of the experience. The dosing session monitor will assess and discuss the participant's symptoms, medical condition and psychological status/well-being following the treatment period.

Each dosing session should be recorded. The recordings will be reviewed to ensure the dosing session monitor is adhering to training and to facilitate training of future dosing session monitors.

5.3.2 Medical Support

During dosing days, a licensed and Basic Life Support (BLS)-certified physician must be on site (or on call and able to reach the clinical site within 15 minutes) in the event of a medical emergency.

5.4 Discharge Criteria

The following criteria must be met prior to discharging a participant:

- No clinically significant suicidal ideation or behaviors are present.
- Vital signs are stable and have returned to near pre-dose values or are otherwise not clinically significant.

- Participant is alert and oriented to person, place, and things. Mental status is appropriate for normal daily activities.

The participant should be discharged to a trusted person.

5.5 Concomitant Medication

Participants should remain on a stable regimen of all allowable medications for the duration of the study.

Participants must agree to abstain from receiving outside psychotherapy or pharmacotherapy to treat their PTSD (or other mental conditions) for the duration of participation in this study (from Screening through to the EOS visit). Prohibited medication may be used at the Investigator's discretion during an acute AE or for the treatment of AEs. Such use may lead to the discontinuation of dosing for that participant.

Section 14.2 and Section 14.3 provides a list of prohibited concomitant medications/therapies.

The Site Investigator and study team will review medication use on a case-by-case basis to determine if its use would compromise participant safety or interfere with study procedures or data interpretation (exclusion criteria relating to concomitant medication are presented in Section 6.2). All concomitant medications used (including over-the-counter [OTC] medications and herbal supplements) will be recorded in the source documents and in the electronic case report form (eCRF).

5.5.1 Prohibited Medications

No formal drug interaction studies with methylone have been conducted in man. Based on the mechanism of action and metabolism of methylone, drug interactions between methylone and other psychoactive substances are possible.

The following medications are prohibited within 8 weeks, or 5 half-lives, whichever is longer of Day 1 and through the End of Study visit:

- Selective serotonin reuptake inhibitor (SSRIs)
- Serotonin and norepinephrine reuptake inhibitors (SNRIs)
- Tricyclic/tetracyclic antidepressants
- Other medications to treat PTSD

The following medications are prohibited within 14 days or 5 half-lives, whichever is longer of Day 1 and through the End of Study visit:

- Lithium and other mood stabilizers
- Antipsychotics
- Stimulants
- Efavirenz
- Strong CYP2D6 inhibitors or inducers
- Serotonin-acting dietary supplements (such as 5-hydroxy-tryptophan or St. John's wort)
- QT-prolonging medications (such as antiarrhythmics and some antibiotics)

The following are restricted within 24 hours of a dosing session:

- Benzodiazepines
- Opioids
- Alcohol

5.6 Special Warnings and Guidance

5.6.1 General Considerations

Each research site should have resuscitation equipment and appropriate staff training, especially the staff member who will remain with the participant during the dose session.

In addition, each research site should be aware of the location of local emergency services/support, how to communicate with such services and how to transport a participant to such services, if needed.

During each dose session, each participant will be in a private space so they cannot see or interact with other individuals for the duration of their dose session. The space will have a quiet, calm atmosphere where they are comfortable talking about personal matters. The space will have ambient temperature controls and access to water.

The participants will be asked to remain in the clinic for the duration of the dose session, for at least 8 hours post-administration.

5.6.2 Recorded Content

All dosing sessions and central rater assessments may be recorded for research and training purposes. Participants may feel uncomfortable with having their sessions recorded. The dosing session recordings are necessary for assessing adherence to the dosing session monitor training and continued development of training material. The recording of central rater assessments is necessary to establish inter-rater reliability.

The sponsor uses encrypted, secure technology to transfer and store recordings, but there is always a risk of a security breach. The sponsor is committed to taking preventative measures to avoid such an event. In the case of a security breach, the participant will be notified, and all efforts will be made to minimize the dissemination of recorded content.

5.6.3 Management of Suicidality

Suicidal ideation or attempts are not anticipated as a result of treatment, but both are recognized complications of PTSD. Furthermore, such thoughts may be provoked by the intense emotional experiences during the Preparatory Session (before any drug administration).

The risk of the development of suicidal ideation is minimized by the inclusion/exclusion criteria and the awareness of the potential issue.

To proactively assess any risk of suicidality, the C-SSRS questionnaire will be administered at every interaction with all participants throughout the duration of the study and used to identify ‘at risk’ participants who should not receive further doses.

Participants experiencing new suicidal ideation or attempt will not receive further dosing. Such participants need immediate medical/psychiatric Follow-Up.

5.6.4 Management of Psychological Events

Drug-related adverse psychological events (e.g., psychotic symptoms) are not anticipated, but may occur during, or following, the dose sessions.

The risk of such events occurring is minimized by the inclusion/exclusion criteria and the Preparatory Session. A physician will be available, defined as the ability to reach the clinical site within 15 minutes, throughout the dosing day to help support any psychological events.

If medical intervention is required, diazepam (5 to 10 mg, orally), lorazepam (1 mg, orally or intramuscularly [IM]) or olanzapine (5 to 10 mg, orally or IM) may be administered, according to the clinical judgement of the responsible physician. Lorazepam and olanzapine should not be given at the same time due to the possibility of excessive sedation and cardiorespiratory depression.

The participant should not be discharged from the clinic until, in the opinion of the Site Investigator, the condition has stabilized.

Participants experiencing a psychological event related to dosing that requires an intervention will not receive further dosing.

If psychological events arise after the participant has left the clinic, they will be instructed to contact the site immediately. Based on the situation, the Investigator and dosing session monitor will determine the most appropriate course of action.

5.6.5 Management of Cardiovascular Events

Elevations in blood pressure and heart rate are to be anticipated.

The risk of such elevations will be minimized by the inclusion/exclusion criteria and frequent monitoring of blood pressure and heart rate during each dose session.

Blood pressure and heart rate will be closely monitored with readings recorded at least every 30 minutes during the dose session. The study team will attend to clinical signs and symptoms of potential rare complications of the cardiovascular effects of methylone, such as stroke or acute myocardial infarction, during dose sessions. Any symptoms such as chest pain, shortness of breath, neurological deficit or confusion, or other potential indicators of end organ effects, will prompt additional vital sign measurements, and intervention if appropriate, and the participant will not receive any further doses of IMP and will be followed until resolution.

If a participant experiences ischemic type chest pain, whether or not it is associated with hypertensive crisis, they will be given 0.4 mg of sublingual nitroglycerin every 5 minutes as needed for chest pain pending transport to a hospital, and the Site Investigator will be promptly notified.

Further evaluation and management will be determined by the appropriate trained healthcare providers.

Participants experiencing a cardiovascular event associated with dosing that requires an intervention will not receive further dosing.

5.6.6 Management of Thermoregulation

Changes in body temperature are not anticipated.

The risk of such changes can be minimized by managing the ambient temperature and minimizing participant activity.

The participant’s temperature will be systematically monitored during the dose session.

If a participant’s temperature rises more than 1°C, or the participant states that they feel hot, attempts will be made to decrease body temperature and increase comfort by removing blankets and layers of clothing, decreasing the ambient temperature and, if necessary, directing a fan toward the participant.

If at any time the temperature rises more than 1.5ºC above pre-dose despite these efforts, the Site Investigator will be consulted for further evaluation and treatment.

Participants experiencing a temperature rise during a dosing session of more than 2ºC above pre-dose (and ≥38ºC) will not receive further dosing.

5.6.7 Management of Osmoregulation

Methylone is not expected to have any risks of osmoregulatory changes.

In order to minimize risk, participants should be encouraged to drink, but intake should be limited to no more than 3 liters over the course of the dose session. Fluid intake should be spread out over the dose session.

If a participant exhibits any signs of toxicity or clinically significant dilutional hyponatremia despite these precautions, they should not receive further dosing.

5.7 Assessment of Compliance with Treatment

All doses will be administered by study staff under direct observation. The administration of the study drug will be recorded within the appropriate pages of the eCRF. Any deviations from the planned dosing procedure will be recorded within the eCRF.

Incorrect dosing will be recorded in the eCRF and reported to the Sponsor or delegate within 48 hours. During this study, any initial administration of methylone greater than 150 mg (>3 capsules), and any booster administration of methylone greater than 100 mg (>2 capsules) will be considered an overdose. In the event of an overdose, the Investigator should:

- Evaluate the participant to determine, in consultation with the Medical Monitor, whether study drug administration should be interrupted (ie, hold the booster dose) or if the booster dose should be reduced.
- Document the quantity of the excess dose.

5.8 Blinding

Part B will be double-blinded (Investigator- and participant-blinded) with observer-blinded assessments of efficacy. The randomization list will be kept in a secure location until the end of the study.

5.9 Emergency Unblinding

The study blind for Part B should not be broken except in a medical emergency where knowledge of the IMP would inform treatment of an emergency.

The Site Investigator has the sole responsibility for determining if unblinding of treatment is necessary for medical management. The Investigators will inform the Medical Monitor and Sponsor of this decision within 48 hours. The applicable procedures will be followed to break the blind.

Emergency unblinding will be performed by the trial supply management system.

After database lock, the overall randomization code will be broken only for reporting purposes.

6 SELECTION OF PARTICIPANTS

The study population includes approximately 15 (Part A) and approximately 64 (Part B) healthy adult participants with PTSD. Each participant must meet all the inclusion criteria and none of the exclusion criteria for this study. Under no circumstances can there be exceptions to this rule.

6.1 Inclusion Criteria

1. Male or female participants aged between 18 to 65 years, inclusive.
2. Participant meets the DSM-5 criteria for current severe PTSD diagnosis, with a symptom duration of at least 6 months at Screening, as assessed by the following psychometric assessments:
    a. CAPS-5 (past month version).
    b. Life Events Checklist for DSM-5 (LEC-5).
    - The PTSD diagnosis will be confirmed at Baseline by the central rater.
3. Participant must have a CAPS-5 score of ≥35 at Screening and ≥28 at Baseline.
4. Participant has failed at least one treatment for PTSD (either psychotherapy or pharmacological treatment).
5. Proficient in reading and writing in local language sufficient to complete questionnaires.
6. Free from any other clinically significant illness or disease that may adversely affect:
    a. the safety of the participant, or,
    b. the integrity of the study (see Section 6.2).
7. Willing to refrain from taking any psychiatric medications or interventions during the study, unless provided by the Site Investigator.
    - No participant will be asked to discontinue potentially beneficial treatment in order to participate in this study.
8. Willing to provide a contact for emergencies.
9. Willing to be contacted via telephone for all necessary telephone contacts.
10. <Removed in Amendment 3>
11. Males who engage in sexual activity that has the risk of pregnancy must agree to use one of the following acceptable contraceptive methods and agree to not donate sperm throughout the study and for at least 90 days after the last dose of the study medication:
    a. Use of a double barrier method (male condom with a diaphragm or a cervical cap)
    b. Female use of hormonal contraceptives (as described below)
    c. Either partner is surgically sterilized (as described below)
    d. Abstinence, if consistent with the participants preferred and usual lifestyle

12. Females of childbearing potential* who are sexually active with a male partner must be willing to use one of the following acceptable contraceptive methods throughout the study and for at least 1 month after the last study drug administration.
    a. Hormonal contraceptives or intrauterine devices: at least 4 weeks before first dosing and must follow that product's package insert instructions including additional protection at times when hormonal contraceptive doses might be missed
    b. Male condom with a diaphragm or cervical cap: consistent use for at least 21 days prior to the first dosing
    c. Sterile male partner: vasectomized for at least 6 months prior to the first dose
    d. Abstinence, if consistent with the participant's preferred and usual lifestyle

    * A non-childbearing female is defined as:
    - Post-menopausal female (absence of menses for 12 months prior to drug administration, bilateral oophorectomy or hysterectomy with bilateral oophorectomy at least 6 months prior to drug administration); OR
    - Surgically sterile female (hysterectomy or tubal ligation at least 6 months prior to drug administration)

    Women of childbearing potential (WOCBP; see Section 14.4) must have a negative highly sensitive urine pregnancy test at Screening, Baseline, and Day 1, and prior to dosing. If a urine test cannot be confirmed as negative (e.g., an ambiguous result), a serum pregnancy test is required to be confirmed as negative prior to dosing.
13. Agree to not participate in any other interventional clinical trials for the duration of this study.
14. Willing to provide written informed consent, which includes compliance with the requirements and restrictions listed in the consent form.

6.2 Exclusion Criteria

1. Participant has a primary diagnosis of any other DSM-5 disorder, as assessed by the MINI Version 7.0.2.
2. Participant has a body mass index (BMI) <18 kg/m^2 or ≥40 kg/m^2.
3. Participant is known (or meets DSM-5 criteria for moderate or severe substance use disorder [mild substance use disorder may be allowed, after consultation with the sponsor, if use of the substance is unlikely to confound the study results]) to abuse illegal drugs or has a positive urine test for illegal drugs at Screening, Baseline, or prior to dosing on Day 1. The Investigator must confirm the participant is not otherwise impaired prior to dosing on Day 1.
4. Known history of:
    a. Hepatitis B,
    b. Hepatitis C, or
    c. Infection with human immunodeficiency virus.
5. Participant smokes an average of >10 cigarettes and/or e-cigarettes per day.
6. Abnormal LFTs at Screening:
    a. AST, ALT or gamma-glutamyl transferase (GGT) >2 × the ULN.

   b. Total bilirubin levels >1.5 × the ULN (participants with a diagnosis of Gilbert's syndrome with high unconjugated bilirubin are eligible provided they meet other LFT criteria).

7. Moderate to severe renal impairment at Screening, with an estimated glomerular filtration rate (eGFR) of ≤80 mL/min according to the Cockcroft-Gault equation.
8. Active suicidal ideation and/or intent within 2 months of Screening, indicated by a 'Yes' response to suicidal ideation question 4 or 5 on the C-SSRS.
9. Any history of suicidal behavior within the last 5 years (excluding suicidal ideation):
   a. Medical history of suicide attempt, or
   b. 'Yes' response to the following suicidal behavior questions on the C-SSRS:
      i. actual attempt, or
      ii. interrupted attempt, or
      iii. aborted attempt.
10. Uncontrolled hypertension at Screening (after 5 minutes rest):
    a. SBP >140 mmHg, or
    b. DBP >90 mmHg.
11. Resting heart rate at Screening of ≥90 bpm (after 5 minutes rest).
12. Have any history, physical or psychological symptoms, medication or other relevant findings that would make a participant unsuitable for the study based on the clinical judgement of study personnel. This would include (but is not limited to):
    a. A need to continue active psychotherapy or pharmacotherapy during the study period.
    b. Moderate or severe alcohol use disorder (as assessed by the MINI Version 7.0.2) *Note: Moderate alcohol use disorder in early remission is not exclusionary*:
       i. the inability to refrain from alcohol use for 24 hours before Screening and each scheduled dose session.
    c. Medical or psychiatric condition that is incompatible with:
       i. establishment of rapport with the dosing session monitor, or
       ii. safe exposure to methylone.
13. Prior treatment within the 90 days prior to Baseline, with:
    a. deep brain stimulation,
    b. vagus nerve stimulation,
    c. treatment with electroconvulsive therapy, or
    d. transcranial magnetic stimulation.

14. Use of a psychedelic (e.g., lysergic acid diethylamide [LSD], psilocybin, dimethyltryptamine [DMT], mescaline), or entactogens such as MDMA, within 12 months of Screening.
15. Use of prohibited concomitant medications or therapies (see Section 5.5.1).
16. Current or previous history of clinically significant cardiovascular/cerebrovascular conditions.
    a. Presence of underlying cardiovascular or cerebrovascular conditions where an acute rise in blood pressure would pose a clinical concern, including but not limited to, aneurysms or arteriovenous malformations, a history of cardiac or cerebral ischemia.
17. Any clinically significant abnormal 12-lead ECG findings at Screening.
    a. Resting QT interval corrected for heart rate using Fridericia's formula (QTcF) ≥450 msec for males, ≥470 msec for females at Screening, or inability to determine QTcF interval.
    b. Presence of risk factors for Torsades de Pointes.
18. Any history of head injury with loss of consciousness for more than 30 minutes within 12 months of Screening.
19. Clinically relevant history of schizophrenia, psychotic disorder, bipolar disorder, delusional disorder, paranoid personality disorder, antisocial personality disorder, schizoaffective disorder, borderline personality disorder or panic disorder, as assessed by the MINI and SPQ (and SCID-5-PD, if needed).
20. Have a first-degree relative with:
    a. schizophrenia spectrum, or
    b. other psychotic disorders (except substance/medication-induced or due to another medical condition), or
    c. Bipolar I disorder.
21. Participant has participated in a clinical study with an IMP within 3 months, or 5 half-lives (whichever is longer), prior to Screening.
22. Has an allergy or intolerance to any of the materials contained in the investigational formulations (methylone or placebo [Part B]).
23. Donation of blood or plasma of >400 mL within 3 months prior to Screening.
24. Family member of a member of the investigating team, Sponsor or Contract Research Organization.

6.3 Study Restrictions

6.3.1 Lifestyle Restrictions

Participants should agree to abstain from strenuous exercise for 24 hours prior to each dose session. Participants are also not allowed to operate any motor vehicle for at least 24 hours after each administration of study drug. Site staff should ensure participants have organized transport after discharge from the study site during the treatment period.

Participants cannot donate blood for the duration of the study until 3 months after the EOS visit.

6.3.2 Meals and Dietary Restrictions

The participants are required to adhere to the following restrictions:

- Participants should abstain from alcohol for 24 hours prior to Screening and each dose session until after discharge from the study site.
- Participants should abstain from tobacco and nicotine-containing products for 2 hours prior to dosing until at least 8 hours after the initial administration on each dosing day.
- Participants should abstain from (methyl) xanthine- and caffeine-containing products for 2 hours prior to dosing until at least 8 hours after the initial administration on each dosing day.
- Participants should have fasted (water only) for at least 2 hours prior to dosing and for at least 2 hours after the booster dose.
- Participants should refrain from consuming poppy seeds 48 hours prior to Screening and Days 1, 8, 15 and 22 to avoid a positive result on the drugs of abuse screen.

6.4 Participant Withdrawal Criteria

Participants may withdraw from the study at any time for any reason. If a participant elects to withdraw before the completion of the study, the participant should be encouraged to attend an Early Termination visit to capture safety and efficacy data.

For all participants withdrawn from the study, discontinuation procedures as described in Schedule of Assessments (Section 1.3) should be conducted prior to discharge in the study.

Reasons for study withdrawal or study drug discontinuation at any time during the study may include any of the following reasons:

- Pregnancy of participant or partner.
- Adverse event: Clinical or para-clinical events occurred that, in the medical judgement of the Site Investigator for the best interest of the participants, are grounds for discontinuation. This includes serious and non-serious AEs regardless of relation to the study drug (see Section 4.8).
- Withdrawal of consent: The participant desired to withdraw from further participation in the study in the absence of an Investigator determined medical need to withdraw. If the participant gave a reason for withdrawing it should be recorded in the eCRF.
- Investigator decision.
- Lost to Follow-Up: The participant stopped coming for visits and study personnel were unable to contact the participant.
- Other: The participant was discontinued for a reason other than those listed above, such as termination of the study by the Sponsor. The reason(s) should be recorded in the eCRF.
- Participant has a positive urine test for an illegal medication (eg, MDMA or cocaine).

- A positive urine drug test for stimulants or if the subject is otherwise impaired will result in missing that dose session.

6.5 Follow-Up Procedures

The Investigator will make diligent attempts to contact participants who fail to return for a scheduled visit or were otherwise unable to attend the EOS visit. All discontinuations and the reason for early discontinuation will be documented by the Investigator, and if appropriate on the AE form.

7 SAFETY ASSESSMENTS

The participants will attend a safety visit on Day 29 (±1 day), Day 36 (±2 days), Day 43 (±2 days) and Day 64 (±3 days) for Follow-Up procedures as shown in the Schedule of Assessments (Section 1.3).

7.1 Clinical Laboratory Evaluations

Clinical laboratory evaluations and urine drug screens will include the analytes listed in Table 4. Approximate blood sampling volumes are provided in Section 14.1.

All blood and urine analyses will be conducted on-site or at a central laboratory at the sampling times indicated in the Schedule of Assessments (Section 1.3). The clinical laboratory evaluation results will be collected at Screening in time for results to be reviewed prior to the first study drug administration. For abnormal values, additional testing may be performed, or clinical laboratory evaluations may be added to evaluate the abnormal values. Clinically significant values should be followed until resolution or until they reach a stable state.

Table 4: Clinical Laboratory Evaluations

**Blood Chemistry**<br>Amylase<br>BUN<br>Creatinine<br>Glucose<br>Sodium<br>Potassium<br>Phosphate<br>Chloride<br>Calcium<br>AST<br>ALT<br>GGT<br>Alkaline phosphatase<br>Total, direct, indirect bilirubin<br>Uric acid<br>Albumin<br>Total protein<br>Lactate dehydrogenase	**Urinalysis**<br>Glucose<br>Bilirubin<br>Ketone<br>Specific Gravity<br>Blood<br>pH<br>Protein<br>Urobilinogen<br>Nitrite<br>Leukocyte Esterase
**Hematology**<br>Hemoglobin<br>Hematocrit<br>RBC count<br>RBC indices (MCV, MCH, MCHC)<br>Platelet count<br>White blood cell count with differential	**Urine drug screen**<br>Amphetamines<br>Benzodiazepines<br>Cocaine<br>MDMA<br>Opiates
**Other Testing**<br>Urine pregnancy test (WOCBP only)<br>Follicle stimulating hormone[a]	

Abbreviations: ALT = alanine aminotransferase; AST = aspartate aminotransferase; BUN = blood urea nitrogen; GGT = gamma-glutamyl transferase; MCH = mean corpuscular hemoglobin; MCHC = mean corpuscular hemoglobin concentration; MCV = mean corpuscular volume; RBC = red blood cell; WOCBP = women of childbearing potential.
a Suspected perimenopausal female participants only, based on surgical history or <12 months amenorrhea.

7.2 12-Lead Electrocardiogram

The 12-lead ECG will be collected within approximately 30 minutes predose and at the end of the dose session. The 12-Lead ECG should be obtained after the participant has rested in the

supine position for at least 5 minutes. The ECG machine used should automatically calculate the HR and PR, QRS, QT and QTcF intervals.

7.3 Vital Signs and Temperature

Vital signs, including HR, BP, and temperature will be collected after the participant has rested in the seated position for at least 5 minutes. Predose vital signs should be collected within 10 minutes predose. Blood pressure and HR will be collected 10 minutes predose and at 30-minute intervals (±15 min) post-initial dose-administration until the end of the dose session. In addition, BP and HR will be recorded 15 minutes (+5 minutes) before administration of the booster dose. This pre-booster vital signs collection is the equivalent of the 90-minute vital signs collection timepoint. Temperature will be recorded predose (within 10 minutes) and at 60-minute intervals (±15 min) post-initial dose-administration, until end of the dose session.

7.4 Physical Examinations

A physical examination, including body weight (Screening and EOS) and height (Screening only), and assessments of the head, eyes, ears, nose, throat, skin, neurological, lungs, cardiovascular system, abdomen, and lymph nodes will be conducted.

7.5 Pregnancy Testing

Dosing should not proceed until a negative pregnancy test is confirmed.

- Refer to Section 6.1 for pregnancy testing entry criteria.
- A urine pregnancy test will be performed at Screening, and Day 1, to confirm eligibility.
- Pregnancy testing (urine) will be conducted prior to dosing on dosing days and on Day 29.
- A positive or inconclusive urine pregnancy test should be confirmed by a serum pregnancy test (see Section 7.8.3.1).
- Additional serum or urine pregnancy tests may be performed, as determined necessary by the Investigator or required by local regulation, to establish the absence of pregnancy at any time during the participant's participation in the study.
    - The Investigator is responsible for review of medical history, menstrual history and recent sexual activity to decrease the risk for inclusion of a woman with an early undetected pregnancy.

7.6 Columbia-Suicide Severity Rating Scale

The C-SSRS will be used to assess suicide potential or tendency as a study entry criterion and monitored throughout the study.

The C-SSRS is a semi-structured interview designed to assess the severity and intensity of suicidal ideation, suicidal behavior and non-suicidal self-injurious behavior over a specified time period. The measurement of suicidal ideation is based on five 'yes' or 'no' questions with accompanying descriptions arranged in order of increasing severity. If the participant answers 'yes' to either questions 1 or 2, the intensity of ideation is assessed in five additional questions related to frequency, duration, controllability, deterrents and reasons for the most severe suicidal ideation. Suicidal behavior is assessed by asking questions categorizing behaviors into actual, aborted and interrupted attempts; preparatory behavior, and non-suicidal self-injurious behavior.

If any item(s) on the C-SSRS are answered ‘yes’, the Investigator must review the participant’s responses in order to (a) at the Screening visit and Baseline/Preparatory session to determine the participant’s study eligibility and potential need for referral to a mental health professional, and (b) during the study evaluate the participant’s need for appropriate medical management such as a referral to a mental health professional, or appropriateness of further study drug administration in consultation with the Medical Monitor and Sponsor.

A significant risk of suicide is defined as a ‘yes’ in answer to:

(a) questions 4 or 5 on the suicidal ideation section; or,

(b) any questions on any item in the suicidal behavior section.

This must be reported as an AE or SAE as appropriate and followed up accordingly. Additionally, if a participant responds ‘yes’ to any of the suicidal ideation questions 1 through 3, the Investigator should apply clinical judgement to determine the need for reporting this as an AE or SAE and the need for any appropriate referral.

For any participant who responds ‘yes’ to relevant questions post-study drug administration, they will be handled appropriately.

7.7 Adverse Event Reporting

Adverse events will be elicited through non-leading questions and spontaneous reporting by participants.

At all times, the Investigator should be alert to comments that imply an AE.

The Investigator is responsible for evaluating AEs and for the appropriate medical care of participants during the study.

7.7.1 Definition and Criteria

7.7.1.1 Adverse Event

An AE is any untoward medical occurrence in a study participant which either emerges, or worsens from enrollment/randomization, during the clinical study, regardless of its potential relationship to the medicinal product. An AE, therefore, can be any unfavorable or unintended sign, including a clinically significant abnormal laboratory finding, symptom, or disease temporally, whether or not it is considered to be study drug-related.

Examples of an AE include:

- Exacerbation of a pre-existing condition including either an increase in frequency and/or severity of the condition.
- New conditions detected or diagnosed.
- Signs or symptoms of a drug interaction.
- Signs or symptoms of a suspected overdose of either IMP or a concurrent medication (overdose per se should not be reported as an AE/SAE).
- A new laboratory abnormality occurring after the start of the study (i.e., following enrollment/randomization) or are present at baseline and significantly worsen following the start of the study that results in discontinuation of the study drug, medical treatment or further follow-up. The Site Investigator will exercise their medical and scientific

judgement in deciding whether an abnormal laboratory finding, or other abnormal assessment is clinically significant.

Note: abnormal laboratory or other values obtained during Screening that preclude a participant from entering the study are not considered AEs but will be recorded as the reason for screen failure.

Adverse events may include pre- or post-treatment events that occur as a result of Protocol-mandated procedures (i.e., invasive procedures, modification of participant's previous therapeutic regimen).

A medical intervention to address an AE is an "action taken" and not an AE itself.

7.7.1.2 Serious Adverse Event

An SAE is any untoward medical occurrence that, at any dose:

- Results in death,
- Is life-threatening,

Note: The term 'life-threatening' in the definition of 'serious' refers to an event in which the participant was at risk of death at the time of the event. It does not refer to an event, which hypothetically might have caused death, if it were more severe.

- Requires hospitalization or prolongation of existing hospitalization,

Note: In general, hospitalization signifies that the participant has been detained (usually involving at least an overnight stay) at the hospital or emergency ward for observation and/or treatment that would not have been appropriate in the physician's office or outpatient setting. Complications that occur during hospitalization are AEs. If complication prolongs hospitalization or fulfils any other serious criteria, the event is serious. When in doubt whether 'hospitalization' occurred or was necessary, the AE should be considered serious.

- Results in persistent or significant disability or incapacity,

Note: The term 'disability' means a substantial disruption of a person's ability to conduct normal life functions. This definition is not intended to include experiences of relatively minor medical significance such as uncomplicated headache, nausea, vomiting, diarrhea, influenza and accidental trauma (e.g., sprained ankle) which may interfere or prevent everyday life functions but do not constitute a substantial disruption.

- Is a congenital abnormality or birth defect,
- Is an important medical event.

Note: Medical or scientific judgement should be exercised in deciding whether reporting is appropriate in other situations, such as important medical events that may not be immediately life-threatening, or result in death or hospitalization, but may jeopardize the participant or may require medical or surgical intervention to prevent one of the other outcomes listed in the above definition. These should also be considered serious. Examples of such events are invasive or malignant cancers, intensive treatment in an emergency room or at home for allergic bronchospasm, blood dyscrasias or convulsions that do not result in hospitalization, or development of drug dependency or drug abuse.

Note: The terms "serious" and "severe" ARE NOT synonymous. The term "severe" is often used to describe the intensity (severity) of a specific event (as in mild, moderate or severe myocardial infarction); the event itself, however, may be of relatively minor medical

significance (such as severe headache). This is NOT the same as "serious", which is based on participant/event outcome or action criteria described above and are usually associated with events that pose a threat to a participant's life or functioning. A severe AE does not necessarily need to be considered serious. For example, persistent nausea of several hours duration may be considered severe nausea but not an SAE. On the other hand, a stroke resulting in only a minor degree of disability may be considered mild but would be defined as an SAE based on the above criteria. Seriousness (not severity) serves as a guide for defining regulatory reporting obligations.

Only SAEs will be recorded from the time of informed consent until the time of enrollment/randomization.

7.7.1.3 Adverse Events of Special Interest

The Sponsor will pay special attention to a subset of AEs; these AEs will be marked in the eCRF with the denotation AESIs whether serious or non-serious. These should be reported to the Sponsor or delegate; they include:

- Grade 3 hypertension, SBP ≥160 mmHg or DBP ≥100 mmHg, requiring the need for medical intervention with more than one drug or more intensive therapy than previously used.
- Significant cardiovascular events
- AEs related to suicide risk, such as; suicides, suicide attempts, self-injurious behavior associated with suicidal ideation and suicidal ideation judged to be serious or severe in the opinion of the Investigator.
- Signals of abuse potential AE involving terms of behavioral addiction, drug abuser, substance abuser.

If an AESI is an SAE or if it involves suicide risk, it should be reported to the Sponsor or delegate within 24 hours of the site's awareness of the event.

7.8 Evaluating Adverse Events and Serious Adverse Events

Care will be taken not to introduce bias when detecting AEs and/or SAEs. Open-ended and non-leading verbal questioning of the participant is the preferred method to inquire about AE occurrences.

All AEs will be assessed on two descriptive parameters: severity and relationship to the study drug:

- Severity refers to the severity of an event and references impact on a participant's functioning.
- Relationship refers to the likelihood that the event being assessed was caused by the study drug.

The Investigator will make an assessment of severity for each AE and SAE reported during the study. Adverse events will be assessed according to the general guidelines for grading:

- **Mild**: Usually transient and may require only minimal treatment or therapeutic intervention. The event does not generally interfere with usual activities of daily living

- **Moderate**: Usually alleviated with additional specific therapeutic intervention. The event interferes with usual activities of daily living, causing discomfort but poses no significant or permanent risk of harm to the subject
- **Severe**: Interrupts usual activities of daily living, or significantly affects clinical status, or may require intensive therapeutic intervention

For AEs and SAEs, the relationship to the study treatment is to be assessed according to the following definitions:

- **Not related:** There is no reasonable association between the study treatment and the suspected event.
- **Unlikely related**: It is doubtful that there is an association between the study treatment and the suspected event. The event could have been produced by the participant's clinical state or other modes of therapy administered to the participant.
- **Possibly related:** The suspected AE may or may not follow a reasonable temporal sequence from study drug administration. The event could have been produced or mimicked by the participant's clinical state or by other modes of therapy concomitantly administered to the participant.
- **Probably related:** The suspected AE follows a reasonable temporal sequence from study treatment administration, abates upon discontinuation of the treatment; and cannot be reasonably explained by the known characteristics of the participant's clinical state.

When assessing the relationship to the study drug, the following criteria will be considered:

- Known class effect
- Positive rechallenge
- Positive dechallenge (resolution after a dose session, in absence of other interventional treatment)
- Biological plausibility
- Lack of alternative explanation – concomitant drug or disease

7.8.1 Reporting Procedures and Requirements

7.8.1.1 Time Period and Frequency for Collecting AE and SAE Information

All SAEs will be collected from the signing of the informed consent form (ICF) until the EOS visit at the timepoints specified in the Schedule of Assessments (see Section 1.3).

All AEs will be collected from enrollment/randomization until the EOS visit at all interactions with the participants (see Section 1.3). A baseline safety assessment will occur after Screening. Medical occurrences that begin before the start of study intervention but after obtaining informed consent will be recorded as medical history/current medical conditions, not as AEs.

The Investigator is not obligated to actively seek information on AEs or SAEs after conclusion of the study participation. However, if the Investigator learns of any SAE, including a death, at any time after a participant has been discharged from the study, and the Investigator considers the event to be reasonably related to the study intervention or study participation, the Investigator must promptly notify the Sponsor.

7.8.1.2 Adverse Events

All AEs between enrollment/randomization to the EOS visit will be recorded in source data and eCRF, whether or not considered study drug-related. All AEs that are possibly or probably related to study drug will be followed until resolution or database lock, whichever occurs first. Also, the sign, symptom, or disease present before the baseline safety assessment are only considered AEs if they worsen after this point. Any AEs already documented at a previous assessment and designated as ongoing should be reviewed at subsequent visits as necessary. If these have resolved, this should be documented. The Investigator should report all AEs on the AE page(s) of the eCRF and source documents, regardless of seriousness, severity, and causality. Whenever possible, an AE will be reported using a diagnostic term, (e.g., "common cold" or "upper respiratory infection" rather than "runny nose, cough, mild fever") and should be described with the attributes described in Section 7.7.1.1.

7.8.1.3 Serious Adverse Events

Each AE will be assessed to determine whether it meets seriousness criteria (Section 7.7.1.2). If the AE is considered serious the Investigator must report this event to the Sponsor or delegate. The Sponsor or delegate will report the event to the IRB/EC according to relevant standard operating procedures (SOPs). Serious adverse events occurring from the time of signed informed consent to the EOS visit or 7 days after the last administration of study drug (whichever occurs later) will be recorded in source and electronic data capture. All serious, treatment-related adverse events, regardless of the time of onset after treatment, should be reported. All information about SAEs will be collected and reported via the SAE form and sent by email message (contact information will be contained in the Investigator site file). At a minimum, the initial report should include the following information:

- Event
- Seriousness criteria
- Protocol number
- Participant number and year of birth
- Study drug (blinded for Part B unless unblinding is required for medical management)
- Reporter name and contact information

In the case of a "minimum report" (one that solely comprises the information bulleted above), a more detailed Follow-Up report should be sent as soon as more information becomes available but no later than 7 calendar days after the date of the initial report. Each SAE should be followed up until resolution or stabilization and for reported deaths, the Investigator should supply Sponsor, or designee, and the IRB/EC with any additional requested information (e.g., autopsy reports and terminal medical reports), if available. The original SAE form should be kept at the study site. The Investigators will be responsible for determining and in turn, reporting SAEs to the Sponsor. The Sponsor, or designee, will be responsible for reporting SAEs to regulatory authorities according to the applicable regulatory requirements. The Sponsor, or designee, will be responsible for completing the safety reports and for notifying the relevant authorities of any SAE as outlined in the International Council for Harmonisation (ICH) Guidelines and per local regulatory requirements.

7.8.2 Prompt Reporting of Serious Adverse Events

Any SAE, occurring in a participant receiving treatment or if the Investigators become aware of any SAE post-treatment during the EOS visit, must be reported by the Investigators to the Medical Monitor within 24 hours even if the SAE does not appear to be medication-related. An emailed copy of the SAE form, in addition to other related information should be sent to the appropriate contact as outlined in the Investigator site file. Additionally, it may be necessary for the Medical Monitor or Sponsor, or delegate, to directly communicate with the Investigators if additional information is required.

7.8.2.1 Follow-Up of Adverse Events

All AEs will be followed to a satisfactory resolution, until it becomes stable, or until it can be explained by another known cause(s) (i.e., concurrent condition or medication) and clinical judgment indicates that further evaluation is not warranted.

Suspected Unexpected Serious Adverse Reactions (SUSARs)

The Sponsor, or designee, is responsible for the reporting of SUSARs in accordance with regulatory guidelines. An AE will be considered unexpected if the nature, severity, frequency of the event is not consistent with the risk information previously described for the IMP. The 'expectedness' for any SAE will be determined against the reference safety information (RSI) included in the IB.

A list of expected potential adverse reactions are provided in the IB.

7.8.3 Special Considerations

7.8.3.1 Pregnancy of Female Participant and/or of Female Partner of Male Participant

Details of all pregnancies in female participants and female partners of male participants will be collected after the start of the study until the end of the study. The Investigators should report pregnancy to the Sponsor or designee within 24 hours after learning of the pregnancy.

If a pregnancy is reported, the Investigator should contact the designated individual(s) following the pregnancy notification process and record information related to the pregnancy on the designated pregnancy form provided.

While pregnancy itself is not considered to be an AE or SAE, any pregnancy complication or elective termination of a pregnancy for medical reasons will be reported as an AE or SAE.

The event meets the SAE criterion only if it results in a spontaneous abortion, congenital anomaly, or reports of suspected adverse reactions in the neonate that are classified as serious.

The female participant or pregnant female partner will be followed to determine the outcome of the pregnancy. The Investigator will collect Follow-Up information on the female participant or pregnant female partner and the neonate, and the information will be forwarded to the Sponsor.

Any post-study pregnancy-related SAE considered reasonably related to the study intervention by the Investigator will be reported to the Sponsor as described in Section 7.8.2. While the Investigator is not obligated to actively seek this information in former study participants or pregnant female partners, they may learn of an SAE through spontaneous reporting.

For pregnant female participants, no further dosing will be permitted.

7.9 Other Assessments

Assessments to be performed at Screening only are detailed in the Schedule of Assessments (Section 1.3).

8 PSYCHOMETRIC ASSESSMENTS

Psychometric assessments will be administered in a neutral, non-leading manner to minimize the chance for bias. Avoiding a biased administration can be achieved by adhering to questionnaire administration guidelines verbatim and only deviating from the script to clarify, re-direct or query further if behavioral examples are needed to determine the appropriate symptom intensity rating.

All clinician-rated psychometric assessments will be administered by a central rater via telephone or video call. Remote assessments assure that the assessor who is collecting the primary outcome measures will not witness dose sessions and the acute effects of methylone, which strengthens the study blind. The central ratings will be recorded in order to ensure quality ratings and rater fidelity across the central rater pool.

Copies of each scale can be found in the Investigator Site File.

8.1 Clinician-Administered Psychometric Assessments

8.1.1 Clinician-Administered PTSD Scale for DSM-5 (CAPS-5)

The CAPS-5 is a 30-item structured interview that can be used to:

- Make current diagnosis of PTSD.
- Assess change in PTSD symptoms over time.

In addition to assessing the 20 DSM-5 PTSD symptoms, questions target the onset and duration of symptoms, subjective distress, impact of symptoms on social and occupational functioning, improvement in symptoms since a previous CAPS administration, overall response validity, overall PTSD severity and specifications for the dissociative subtype (depersonalization and derealization). The CAPS-5 also rates intrusion symptoms (intrusive thoughts or memories), avoidance, cognitive and mood symptoms, arousal and reactivity symptoms, duration and degree of distress. For each symptom, standardized questions and probes will be provided.

The ‘past month’ version of the CAPS-5 will be performed at Screening. The ‘past week’ version of the CAPS-5 will be performed at each subsequent study visit. The participant will report on the same Criterion A event specified at Screening for all CAPS-5 assessment timepoints.

Each CAPS-5 will be administered by a central rater via telephone or video call.

8.1.2 Mini International Neuropsychiatric Interview

The MINI 7.0.2 is a short, structured diagnostic interview that is compatible with DSM-5 and International Classification of Disease criteria for psychiatric disorders. Each module of the MINI consists of two or three questions where the answer is either ‘Yes’ or ‘No’, and a decision-tree logic is used to determine whether to ask additional questions. The PTSD, suicidality, and antisocial personality disorder modules of the MINI are not required to be administered as these are addressed via other scales.

The MINI will be administered at Screening by a central rater.

8.1.3 Structured Clinical Interview for DSM-5 Personality Disorders (SCID-5-PD)

The SCID-5-PD is a structured diagnostic interview to assess for DSM-5 personality disorders.

In this study, participants who endorse a positive response for paranoid personality disorder, antisocial personality disorder, or borderline personality on the SPQ will be administered the SCID-5-PD for those personality disorders.

The SCID-5-PD, if needed, will be administered at Screening by a central rater.

8.1.4 Structured Interview Guide for the Montgomery-Åsberg Depression Rating Scale (MADRS) (SIGMA)

The SIGMA is a standardized version of the MADRS. The SIGMA maintains the original format of the MADRS, which is a 10-item clinician-rated, diagnostic questionnaire used to measure depression severity, with the exception that the first two items are reversed. Each item is measured on a 7-point scale, from 0 to 6. Higher total MADRS scores indicate more severe depression. Follow-up questions are also provided to clarify symptoms, if required.

The MADRS will be administered at by a central rater.

8.1.5 Clinician Global Impressions Scale – Severity

The CGI-S is designed to acquaint the participant's severity of symptoms with those of other people experiencing the same mental ailment. The CGI-S rates this severity on a 7-point scale, with (1) representing normal symptoms, meaning the participant is not ill, and (7) representing participants among the most severely ill. The rating (4) represents a participant that is defined a participant that is defined as moderately ill.

The CGI-S will be administered at by a central rater.

8.1.6 Clinician Global Impressions Scale – Improvement

The CGI-I is a 7-point scale that requires the assessor to assess how much the participant's illness has improved or worsened relative to a baseline state prior to dosing. The CGI-I scale rates improvement with (1) representing a 'very much improved' participant to (7) representing a participant who has become 'very much worse' due to treatment. The rating (4) represents a participant displaying no change from the treatment.

The CGI-I will be administered at by a central rater.

8.2 Participant-Completed Psychometric Assessments

8.2.1 PTSD Checklist for DSM-5 (PCL-5)

The PCL-5 is a 20 item self-report measure that assesses the presence and severity of the 20 DSM-5 symptoms of PTSD. The PCL-5 has a variety of purposes, including:

- Monitoring symptom changes during and after treatment.
- Screening individuals for PTSD.
- Making a provisional PTSD diagnosis.

Participants will be asked to rate each item from 0 ('not at all') to 4 ('extremely') to indicate the degree to which they have been affected by that particular symptom over the past week; the results will be interpreted by a clinician.

8.2.2 Life Events Checklist for DSM-5 (Extended Version)

The LEC-5 is a brief, 17-item self-report measure designed to screen for potentially traumatic events in a participant's lifetime, to facilitate the diagnosis of PTSD. It is a companion to the PCL-5 and will be used to assess PTSD. The extended version of the LEC-5 should be used to establish the worst event if more than one event occurred.

8.2.3 Structured Clinical Interview for DSM-5 Screening Personality Questionnaire (SCID-5-SPQ)

The SCID-5-SPQ is a self-report screening tool to assess for DSM-5 personality disorders.

Participants will be required to only complete questions specific for assessing personality disorders (paranoid personality disorder, antisocial personality disorder, or borderline personality). In this study, participants who endorse a positive response for paranoid personality disorder, antisocial personality disorder, or borderline personality on the SPQ will be administered the SCID-5-PD for those personality disorders.

8.2.4 Sheehan Disability Scale

The SDS is a brief, 5-item self-report tool that assesses functional impairment in work, social life/leisure activities and family life/home responsibilities. The SDS is designed to measure the extent to which the three major domains in the participant's life are functionally impaired by psychiatric or medical symptoms. Each domain is rated from 0 to 10, with a total score from 0 to 30, where 0 – Unimpaired and 30 – Highly impaired.

8.2.5 Patient Global Impression of Change

The PGI-C is a self-report tool to reflect a participant's belief about the efficacy of treatment. The PGI-C is a 7-point scale depicting a participant's rating of overall improvement. Participants rate their change from '1 – Very much improved' through to '7 – Very much worse'.

8.2.6 Patient Global Impression of Severity

The PGI-S is a self-report tool to measure disease severity. The PGI-S is a 5-point scale, ranging from '1 – None', through to '5 – Very severe'.

8.2.7 Warwick-Edinburgh Mental Wellbeing Scale

The WEMWBS is a scale of 14 positively worded items for assessing a population's mental wellbeing. The items cover both physiological functioning aspects of mental wellbeing (including: optimism, autonomy, agency, curiosity, clarity of thought and positive relationships) and positive effect (feelings, including: confidence, feeling relaxed, cheerful, having the energy to spare). The scale has 5 response categories, summed to provide a single score.

8.2.8 Post-Traumatic Growth Inventory

The Post-Traumatic Growth Inventory measures the extent to which survivors of traumatic events perceive personal benefits, including changes in perceptions of self, relationships with others and philosophy of life, accruing from their attempts to cope with trauma and its aftermath. This 21-item scale includes factors of: new possibilities, relating to others, personal strength, spiritual change and appreciation of life.

8.2.9 Beck Depression Inventory-II

The BDI-II is a 21-item self-report measure assessing depressive symptoms and their severity. Items include hopelessness and irritability, cognitions such as guilt or feelings of being punished, as well as physical symptoms such as fatigue and weight loss. Each item is rated on a 4-point scale, from 0 to 3. Items are summed to create a total score, with higher scores indicating higher levels of depression.

8.3 Sleep Quality Assessments

8.3.1 Pittsburgh Sleep Quality Index

The PSQI is a measure of self-reported sleep quality and sleep disturbance. The PSQI contains 19 self-rated questions, which are combined to form 7 'component' scores, each of which has a range of 0 to 3 points. The 7 components of the PSQI are: subjective sleep quality, sleep latency, sleep duration, habitual sleep efficiency, sleep disturbances, use of sleeping medications and daytime dysfunction. The sleep component scores are summed to yield a total score ranging from 0 to 21, with a higher total score indicating worse sleep quality.

[redacted] [redacted]

[redacted]

[redacted]

- [redacted]
- [redacted]
- [redacted]
- [redacted]

[redacted]

[redacted]

8.4.3 Blinding Integrity Questionnaire (Part B only)

The BIQ is a visual analogue scale that will be used to ascertain the degree of blinding; participants will be asked to circle a number from 0 (Strongly believe they received Placebo) to 10 (Strongly believe they received active drug) indicating which treatment they believed they were administered during the dose sessions.

The BIQ is administered during Part B only.

9 STATISTICS AND DATA ANALYSIS

9.1 Randomization and Participant Allocation

After informed consent is obtained, participants will be allocated a unique study number.

Only participants who comply with all the inclusion criteria, and none of the exclusion criteria will be randomized/enrolled onto the study. For Part A eligible participants will be enrolled into the open-label study.

For Part B, a randomization scheme will be produced by an unblinded Statistician in SAS 9.4 or higher. The participants will be randomized 1:1 in block sizes of 4 (2 treatments, 2 placebo) to the appropriate treatment (methylone or placebo) using an electronic Randomization and Trial Supply Management.

The participants in Part B will be assigned a randomization number in the order of randomization.

9.2 Sample Size Determination

No formal sample size calculation was performed for Part A of the study. The sample size of approximately 15 participants in the open-label study is based on medical and clinical considerations.

Sample size calculations for Part B were performed using PASS 2022 to determine the sample size required to detect a significant difference between CAPS-5 scores in the methylone and placebo groups (the efficacy endpoint). A previous phase 3 trial investigating the effects of MDMA versus placebo on CAPS-5 scores found a placebo-corrected treatment effect at the end of treatment of 11 points and a pooled standard deviation (SD) of 12 (Mitchell 2021). Assuming a treatment effect of 11 points and a pooled SD of 12 in this trial, with a sample size of 20 or 27 evaluable participants in each treatment group, the trial should provide 80% or 90% power, respectively, to detect a treatment difference at two-sided significance level of 0.05. Assuming a dropout rate of 15%, a target sample size of 46 (23 per treatment group) or 64 (32 per treatment group) will provide 80% or 90% power, respectively.

A DSMB will monitor the clinical data.

An N of 20 or more in each treatment group should also provide useful safety and tolerability data, without exposing too many individuals.

9.3 Statistical Analysis Plan

A Statistical Analysis Plan (SAP) will be prepared after finalizing the Protocol and before database lock. The methods describe in these sections may be modified, for example, due to regulatory feedback. The final analysis methods will be described in the Final SAP that must be approved before database lock. The SAP will provide full details of the analyses, data displays, and algorithms to be used for data derivation.

9.4 Analysis Sets

Unless otherwise stated in the SAP, the analysis set definitions apply to both Parts A and B of the study.

9.4.1 Intent-to-Treat (ITT) Population

The ITT population for Part A includes all participants who receive at least one administration of study treatment. Participants are analyzed on the treatment to which they received for Part

A. The ITT population for Part B includes all participants who are randomized. Participants are analyzed on the treatment to which they are randomized to receive for Part B.

9.4.2 Per-Protocol Population

The Per-Protocol population includes all participants in the ITT population who received at least one administration of study treatment and have no important major protocol deviations.

9.4.3 Safety Population

The Safety population includes all participants who receive at least one administration of study treatment. Participants will be summarized according to treatment received. The Safety population will be used to analyze the safety endpoints.

9.5 Statistical Analysis

Statistical analyses will be performed using SAS 9.4 or higher.

9.5.1 Safety Statistical Analysis

Safety assessments will include clinical laboratory evaluations (clinical chemistry, hematology and urinalysis), vital signs (HR, BP and body temperature), 12-lead ECG, physical examinations, C-SSRS and AE monitoring.

9.5.1.1 Adverse Events

All AEs will be listed according to system-organ class (SOC) and preferred term (PT) assigned to the event using Medical Dictionary for Regulatory Activities (MedDRA). The number and percentage of participants experiencing any AEs will be summarized overall and by treatment for Part B, causality and maximum severity. Any SAEs, TEAEs and/or AEs that led to withdrawal will also be listed in the same manner.

The number and percentage of participants experiencing TEAEs, SAEs, and AEs leading to withdrawal from the trial drug will be tabulated by MedDRA SOC, PT, causality, and maximum severity. The AE tables will also include frequency counts for each SOC and PT.

All AESIs will be summarized similarly to the AEs.

9.5.1.2 Clinical Laboratory Evaluations

Clinical Laboratory evaluation results will be listed and compared to laboratory reference ranges, with those values outside of the applicable range flagged as high (H) or low (L). Clinical chemistry and hematology data will be summarized using descriptive statistics (n, arithmetic mean, SD, median, minimum and maximum) for each parameter overall and by treatment for Part B and trial period. Urinalysis data will be listed only.

9.5.1.3 Vital Signs

Vital signs (SBP, DBP, HR, respiratory rate and body temperature) will be listed for individual participants. Descriptive statistics (n, arithmetic mean, SD, median, minimum and maximum) will be calculated and plotted for each parameter overall and by treatment for Part B, trial period, and timepoint.

9.5.1.4 12-Lead Electrocardiogram

Standard 12-lead ECG parameters (PR, QRS, QT, and QTcF intervals, and HR) will be listed for individual participants. Descriptive statistics (n, arithmetic mean, SD, median, minimum and maximum) will be calculated for each parameter overall and by treatment for Part B and trial period. Where multiple values are recorded at a time for a participant, the mean of the values will be used in the descriptive statistics.

9.5.1.5 Physical Examination

Physical examination data will be listed by participant for abnormal findings only.

9.5.1.6 C-SSRS

C-SSRS data, including item scores, suicidal ideation and suicidal behavior will be listed for individual participants. Scores will be summarized using descriptive statistics (n, arithmetic mean, SD, median, minimum and maximum) overall and by treatment for Part B and trial period.

9.5.2 Efficacy Statistical Analysis

The primary efficacy endpoint is the CAPS-5 total score change from baseline to Week 10 as compared to placebo.

All efficacy endpoint data will be listed for individual participants. All continuous efficacy endpoints will also be summarized by descriptive statistics (n, arithmetic mean, SD, median, minimum and maximum) by study part, overall, by treatment (for Part B) and study period. All categorical efficacy endpoints will be summarized by descriptive statistics (n, percentage).

For Part A the change from baseline to each timepoint will be presented along with a paired *t*-test p-value.

A Mixed Model for Repeated Measures (MMRM) will be fitted for select efficacy endpoints to analyze the difference between treatments for Part B. The model will include treatment, visit, and treatment by visit interaction as fixed effects, where visit is the repeated effect. The baseline value will be included (if applicable) as a covariate. All tests will be based on two-sided significant level of 0.05. Full details of the statistical analysis for each endpoint will be described in the SAP.

9.6 Handling of Missing or Incomplete Data

Unrecorded values will be treated as missing. The appropriateness of the method(s) described for handling missing data will be reassessed and documented as the data review prior to database lock. Depending on the extent of the missing values, further investigation may be made into sensitivity of the analysis results to the method(s) specified.

10 DATA HANDLING AND RECORD KEEPING

10.1 Collection of Data

Data collected from each completed participant will be recorded on source documents, which will be entered into an eCRF. The Investigator is responsible for ensuring that all sections of the eCRF are completed correctly and that entries can be verified against the source documents. If certain data are not available or not applicable this will be indicated as such within the appropriate area of the eCRF.

Screening failures are defined as participants who signed consent to take part in the study but were not subsequently assigned to study intervention. Screening failures may fail to meet any inclusion criteria and may meet one or more exclusion criteria or withdraw consent prior to enrollment. All potential participants who begin Screening will be tracked on a Screening Log. Screening failures are not considered evaluable. Minimal data on screening failures will be recorded in the clinical study database. A minimal set of screen failure information is required to ensure transparent reporting of screen failure participants to meet the CONSORT publishing requirements and to respond to queries from regulatory authorities. Minimal information includes demography, screen failure details, eligibility criteria, and any SAEs.

Data produced by automatic devices with original printouts (i.e., clinical laboratory evaluation results, ECG traces) will be attached to the source documents. Clinical laboratory parameters will be provided in laboratory printouts which are to be signed by the Investigators. Comments on all clinically significant abnormal values will be provided and documented by the Investigators or appropriately recorded within the eCRF.

Adverse Events and Medical History will be reported by the MedDRA SOC and PT; the most current version at the time of study start will be applied to all terms. All Medical History and AEs will be included in the data listings. Furthermore, all Prior Medications and Concomitant Medications will be reported using the WHODRUG categorization; the most current version at the time of study start will be applied to all terms.

All Prior Medications and Concomitant Medications will be summarized within the data listings.

Any missing, implausible, or inconsistent recordings within the eCRFs will be referred to the Investigator using data query validation procedures; will be documented and resolved for each individual participant before database lock is declared.

All processes pertaining to the Data Management activities will be detailed within the Data Management Plan.

10.2 Inspection of Records

Authorized representatives of the Sponsor or delegate will be allowed to conduct site visits for the purpose of monitoring any aspect of the study. The Investigator agrees to allow the monitor to inspect the drug storage area, study drug stocks, drug accountability and participant records, study source documents and any other pertinent records relative to study conduct.

10.3 Retention of Records

The Investigator should maintain all documentation relating to the study for a period of 25 years or per local regulations, whichever is longer. If it becomes necessary for the Sponsor or delegate or a regulatory authority to review any documentation relating to the study, the Investigator must permit access to such records.

10.4 Confidentiality and Data Protection

All data collected in this study will be recorded, processed, stored, and handled in such a way that it can be accurately reported, interpreted, and verified, while preserving the confidentiality of the records. Every effort will be made to strictly safeguard the confidentiality of participants. Participants will be assigned a participant number/identifier and study data will be associated only with this number/identifier. Source documents will be stored securely at clinical sites and access will be limited to regulatory agencies, researchers, and individuals analyzing data. Study data will be recorded in a secure EDC system compliant with 21 CFR Part 11 and applicable regional (i.e. EU) directives. All data entered is de-identified and participants are referred by their assigned participant number/identifier.

The Sponsor serves as Data Controller according to the General Data Protection Regulation (GDPR) and maintains robust contractual agreements with any vendor processing participant data. These contractual agreements outline the technical and organizational measures that comply with Data Protection Laws and ensure a level of security appropriate to the risk of processing participants data.

11 MONITORING, AUDIT, AND INSPECTION

11.1 Study Monitoring

Before an Investigator can enter a participant into the study, a representative of the Sponsor or delegate will visit the site to:

- Determine the adequacy of the facilities and staff,
- Discuss with the Investigator and other personnel their responsibilities with regard to Protocol adherence, and the responsibilities of the Sponsor or delegate. This will be documented in a Clinical Trial Agreement between the Sponsor or delegate and the Investigator.

During the study, a Clinical Research Associate from the Sponsor or delegate will have regular contacts with the site, for the following:

- Provide information and support to the Investigator.
- Confirm that facilities remain acceptable.
- Confirm that the investigational team is adhering to the Protocol, that data are being accurately recorded in the eCRFs, and that study drug accountability checks are being performed.
- Perform source data verification. This includes a comparison of the data in the eCRFs with the participant's medical records at the hospital or practice, and other records relevant to the study. This will require direct access to all original records for each participant (e.g., clinic charts).
- Record and report any protocol deviations not previously sent to the Sponsor or delegate.
- Confirm AEs and SAEs have been appropriately documented within eCRFs and confirm any SAEs have been communicated to the Sponsor or delegate and those SAEs that met criteria for reporting have been provided to the IRB/EC.

The Clinical Research Associate will be available between visits if the Investigator or other staff needs information or advice.

11.2 Audits and Inspections

Authorized representatives of the Sponsor or delegate, a regulatory authority, or the IRB/EC may visit the site to perform audits or inspections, including source data verification. The purpose of an audit or inspection is to systematically and independently examine all study-related activities and documents. The inspection determines whether these activities were conducted, in addition to data being recorded, analyzed, and accurately reported according to the Protocol; ICH-GCP and any applicable regulatory requirements. The Investigator is responsible for making contact with the Sponsor or delegate immediately, if approached by a regulatory agency regarding an inspection.

11.3 Quality Control and Quality Assurance

To ensure compliance with GCP and all regulatory requirements, the Sponsor or delegate may conduct a Quality Assurance Audit.

12 ETHICAL AND REGULATORY CONSIDERATIONS

12.1 Ethical Conduct of the Study

The study will be conducted in accordance with this protocol, the principles of Good Clinical Practice and applicable regulatory requirement(s) (i.e. the EU Clinical Trial Regulation 536/2014, the ICH guideline for GCP E6(R2) dated December 2016, and the ethical principles laid down in the Declaration of Helsinki). Current regulatory requirements will be followed, as applicable.

12.2 Approval by Institutional Review Board / Ethics Committee

A valid IRB/EC must review and approve this protocol before study initiation. Written notification of approval is required before shipment of investigational drug supplies and will include the date of the committee's approval and the chairperson's signature.

Until written approval by the IRB/EC has been received by the investigator, no participant may undergo any procedure solely for determining eligibility for this study.

Protocol amendments must also be reviewed and approved in writing by the IRB/EC prior to implementation.

12.3 Written Informed Consent

Potential participants will have a detailed verbal presentation of the nature, purpose, risks and requirements, in addition to receiving detailed written information provided in the Participant Information Sheet. They will have adequate opportunity to ask the healthcare professional presenting the study about any aspect of the study. Participants must also be notified that they are free to discontinue from the study at any time. The participant should be given the opportunity to ask questions and allowed time to consider the information provided. Once the participant is satisfied that they are willing to participate in the study, they will be asked to sign a copy of the study ICF. The participant's signed and dated informed consent must be obtained before conducting any study procedures. The Investigator must maintain the original, signed ICF. A copy of the signed ICF will be provided to the participant.

If the participant withdraws consent for disclosure of future information, the Sponsor may retain and continue to use any data collected before such withdrawal of consent. If a participant withdraws from the study, the participant may request destruction of any samples taken and not tested, and the Investigator must document this in the site study records.

Records up to the time of early termination should be completed. In the event that a participant does not receive a study treatment, the primary reason will be recorded. A list of the procedures conducted at Screening are presented in the Schedule of Assessments (see Section 1.3).

Informed consent will be obtained prior to the participant undergoing procedures that are specifically for the purposes of the study.

13 REFERENCES

Arroll B, Macgillivray S, Ogston S, et al. Efficacy and tolerability of tricyclic antidepressants and SSRIs compared with placebo for treatment of depression in primary care: a meta-analysis. *Annals of Family Medicine*. 2005;3(5):449-456.

Averill LA, Perelman M, Ching THW, Farré M, Mandell B, Stogniew M, et al. A Case Series Providing Clinical Evidence that Methylone Produces Rapid and Robust Improvements in Major Depressive Disorder. *Ann Clin Case Rep*. 2023; 8: 2519

Baumann MH, Walters HM, Niello M, Sitte HH. Neuropharmacology of Synthetic Cathinones. *Handb Exp Pharmacol*. 2018;252:113-142.

Cameron KN, Kolanos R, Solis E Jr, Glennon RA, De Felice LJ. Bath salts components mephedrone and methylenedioxypyrovalerone (MDPV) act synergistically at the human dopamine transporter. *Br J Pharmacol.* 2013;168(7):1750-1757.

Dorrington S, Zavos H, Ball H, et al. Trauma, post-traumatic stress disorder and psychiatric disorders in a middle-income setting: prevalence and comorbidity. *Br J Psychiatry*. 2014;205(5):383-9.

Cipriani A, Williams T, Nikolakopoulou A, et al. Comparative efficacy and acceptability of pharmacological treatments for post-traumatic stress disorder in adults: a network meta-analysis. *Psychological Medicine.* 2018;48(12):1975-1984.

Eshleman AJ, Wolfrum KM, Hatfield MG, et al. Substituted methcathinones differ in transporter and receptor interactions. *Biochem Pharmacol.* 2013;85(12):1803-15.

Flory JD, Yehuda R. Comorbidity between post-traumatic stress disorder and major depressive disorder: alternative explanations and treatment considerations. *Dialogues in Clinical Neuroscience.* 2015;17(2):141-150.

Forbes D, Creamer M, Bisson JI, et al. A guide to guidelines for the treatment of PTSD and related conditions. *Journal of Traumatic Stress*. 2010;23(5):537-552.

Greenberg N, Brooks S, Dunn R. Latest developments in post-traumatic stress disorder: diagnosis and treatment. *Br Med Bull.* 2015;114(1):147-55.

Hassan Z, Bosch OG, Singh D, et al. Novel Psychoactive Substances-Recent Progress on Neuropharmacological Mechanisms of Action for Selected Drugs. *Front Psychiatry.* 2017;8:152.

Hoskins M, Pearce J, Bethell A, et al. Pharmacotherapy for post-traumatic stress disorder: systematic review and meta-analysis. *Br J Psychiatry*. 2015;206(2):93-100.

Jones, A., Warner-Schmidt, J., Stogniew, M., et al. Methylone for the treatment of PTSD: initial results from an open-label study (IMPACT-1), 2023 ACNP, Tampa, FL.

Kato T, Furukawa TA, Mantani A, et al. Optimising first- and second-line treatment strategies for untreated major depressive disorder — the SUN☺D study: a pragmatic, multi-centre, assessor-blinded randomised controlled trial. *BMC Medicine.* 2018;16:103.

Kelmendi B, Pittenger C, Farre M, et al. Clinical Evidence for the Use of Methylone in the Treatment of PTSD: A Case Series with Long-Term Follow-up. *Annals of Clinical Case Reports*. 2022; 7: 2209

Kessler RC, Sonnega A, Bromet E, Hughes M, Nelson CB. Posttraumatic stress disorder in the National Comorbidity Survey. *Archives of General Psychiatry*. 1995;52(12):1048-1060.

Kibler JL. Posttraumatic stress and cardiovascular disease risk. *J Trauma Dissociation*. 2009;10(2):135-50.

Koenen KC, Ratanatharathorn A, Ng L, et al. Posttraumatic stress disorder in the World Mental Health Surveys. *Psychol Med.* 2017;47(13):2260-2274.

McManus S, Bebbington PE, Jenkins R, Brugha T. Mental health and wellbeing in England: Adult Psychiatric Morbidity Survey 2014. Leeds: NHS Digital, 2016.

Mitchell JM, Bogenschutz M, Lilienstein A, et al. MDMA-assisted therapy for severe PTSD: a randomized, double-blind, placebo-controlled phase 3 study. *Nat Med.* 2021;27(6):1025-1033.

National Health Service (NHS). Causes - Post-traumatic stress disorder. Accessed March 27, 2023. https://www.nhs.uk/mental-health/conditions/post-traumatic-stress-disorder-ptsd/causes/

National Institute of Mental Health (NIMH). Post-Traumatic Stress Disorder (PTSD). Accessed March 27, 2023. https://www.nimh.nih.gov/health/statistics/post-traumatic-stress-disorder-ptsd

Peconga EK, Høgh Thøgersen M. Post-traumatic stress disorder, depression, and anxiety in adult Syrian refugees: What do we know? *Scandinavian Journal of Public Health*. 2020;48(7):677-687.

Poyatos L, Papaseit E, Olesti E, et al. A Comparison of Acute Pharmacological Effects of Methylone and MDMA Administration in Humans and Oral Fluid Concentrations as Biomarkers of Exposure. *Biology (Basel)*. 2021;10(8):788.

Poyatos L, Lo Faro AF, Berardinelli D, et al. Methylone and MDMA Pharmacokinetics Following Controlled Administration in Humans. *Int J Mol Sci.* 2022;23(23):14636.

Poyatos L, Pérez-Mañá C, Hladun O, et al. Pharmacological effects of methylone and MDMA in humans. *Front Pharmacol.* 2023;14:1122861.

Scott KM, McGee MA, Wells JE, Oakley Browne MA. Obesity and mental disorders in the adult general population. *J Psychosom Res*. 2008;64(1):97-105.

Shulgin A, inventor; Methylone Patent, International Application Published under the Patent Cooperation Treaty. December 12, 1996.

Stein DJ, Ipser JC, Seedat S. Pharmacotherapy for post traumatic stress disorder (PTSD). *Cochrane Database Syst Rev.*. 2006;(1):CD002795.

Stein MB, Kennedy C. Major depressive and post-traumatic stress disorder comorbidity in female victims of intimate partner violence. *Journal of Affective Disorders*. 2001;66(2-3):133-138.

Tarrier N, Gregg L. Suicide risk in civilian PTSD patients--predictors of suicidal ideation, planning and attempts. *Soc Psychiatry Psychiatr Epidemiol.* 2004;39(8):655-61.

Warner-Schmidt J, Pittenger C, Stogniew M, Mandell B, Olmstead SJ, Kelmendi B. Methylone, a rapid acting entactogen with robust anxiolytic and antidepressant-like activity. *Front Psychiatry.* 2023;13:1041277.

Watterson LR, Hood L, Sewalia K, et al. The Reinforcing and Rewarding Effects of Methylone, a Synthetic Cathinone Commonly Found in "Bath Salts". *J Addict Res Ther.* 2012;Suppl 9:002

World Health Organization. (2014). Methylone (bk-MDMA) critical review report. Expert Committee on Drug Dependence 36th Meeting, Geneva, Switzerland. https://legal-high-inhaltsstoffe.de/sites/default/files/uploads/methylone.pdf Accessed: March 27, 2023.

Yu A, Basu A, Dibbs M, et al. Circuit- and behavioral-level investigation of plasticity after administration of psychedelics and entactogens in mice. *Society for Neuroscience 2022 Annual Meeting*, San Diego, CA, USA

14 APPENDICES

14.1 Appendix 1– Sampling Summary

The following table summarizes the approximate number of samples and volumes for sampling during the study:

Sample Type	Sample Purpose	Number of Samples[a] (Volume Per Sample)	Total Volume (mL)
Blood	Hematology	4 (2 mL)	8 mL
Blood	Clinical Chemistry	4 (5 mL)	20 mL
	**Total Blood:**		**28 mL**

a Additional samples may be drawn if needed for safety reasons.

14.2 Appendix 2 – Prohibited Medications – Psychoactive Substances

Use of any medication that would affect a participant's mental state is prohibited for 8 weeks or 5 half-lives (whichever is longer) prior to Day 1 and for the duration of the study.

Potential medications include the following (not exhaustive):

Drug Class and Generic Name (Brand Name, if available)
**Antidepressants – Tricyclic/Tetracyclic**
Amitriptyline (ELAVIL®)
Amoxapine (ASENDIN®)
Clomipramine (ANAFRANIL®)
Desipramine (NORPRAMIN®)
Doxepin (SINEQUAN®)
Imipramine (TOFRANIL®)
Maprotiline (LUDIOMIL®)
Mirtazapine (REMERON®)
Nortriptyline (PAMELOR®)
Protriptyline (VIVACTIL®)
Trimipramine (SURMONTIL®)
**Antidepressants – SSRI/SNRI**
Citalopram (CELEXA®)
Desvenlafaxine (PRISTIQ®)
Duloxetine (CYMBALTA®)
Escitalopram (LEXAPRO®)
Fluoxetine (PROZAC®)
Fluvoxamine (LUVOX®)
Levomilnacipran (FETZIMA®)
Milnacipran (SAVELLA®)
Paroxetine (PAXIL®)
Sertraline (ZOLOFT®)
Venlafaxine (EFFEXOR®)
**Antidepressants - MAOI**
Isocarboxazid (MARPLAN®)
Phenelzine (NARDIL®)
Selegiline (ENSAM®, ELDEPRYL®)
Tranylcypromine (PARNATE®)
**Antidepressants - OTHER**
Mirtazapine (REMERON®)
Nefazodone (SERZONE®)
Trazodone (DESYREL®, OLEPTRO®)
Vilazodone (VIIBRYD®)
Vortioxetine (TRINTELLIX®)

14.3 Appendix 3 – Prohibited Medications/Elimination Half-Life - Drug Interactions

Use of any medication that might have a drug-drug interaction with methylone is prohibited for 14 days or 5 half-lives (whichever is longer) before Screening and for the duration of the study.

These include (not exhaustive):

Drug Class and Generic Name (Brand Name, if available)
**Uridine Diphosphate (UDP) or Glucuronosyltransferase (UGT) inhibitors or inducers**
Atazanavir (REYATAZ®)
Diclofenac (topical diclofenac formulations are allowed) (VOLTAREN®)
Mycophenolic Acid (MYFORTIC®)
Quinidine (QUINAGLUTE®)
Ritonavir (NORVIR®)
Silybin
Valproate / Valproic acid (CONVULEX®)
Phenobarbital (LUMINAL®)
**Antipsychotics – Traditional**
Chlorpromazine (THORAZINE®)
Fluphenazine (PROLIXIN®)
Haloperidol (HALDOL®)
Loxapine (LOXITANE®)
Mesoridazine (SERENTIL®)
Molindone (MOBAN®)
Perphenazine (TRILAFON®)
Prochlorperazine (COMPAZINE®)
Thioridazine (MELLARIL®)
Thiothixene (NAVANE®)
Trifluoperazine (STELAZINE®)
**Antipsychotics – Atypical**
Aripiprazole (ABILIFY®)
Asenapine (SAPHRIS®)
Brexpiprazole (REXULTI®)
Cariprazine (VRAYLAR®)
Clozapine (CLOZARIL®)
Iloperidone (FANAPT®)
Olanzapine (ZYPREXA®)
Paliperidone (INVEGA®)
Pimavanserin (NUPLAZID®)
Quetiapine (SEROQUEL®)
Risperidone (RISERDAL®)
Ziprasidone (GEODON®)
Olanzapine/samidorphan (LYBALVI®)
**Miscellaneous**
St John's Wort
S-adenosyl-methionine (SAM-e)
Efavirenz (SUSTIVA®)
5-Hydroxytryptophan (5-HTP)
L-Methylfolate ($\geq$ 7.5 mg/day)
Lithium
Lamotrigine
Carbamazepine/ Oxcarbazepine

**Drug Class and Generic Name (Brand Name, if available)**
**Strong CYPD2D6 Inhibitors (not already listed elsewhere)**
Bupropion (WELLBUTRIN®)
**QT-prolonging medications (not already listed elsewhere)**
Amiodarone
Sotalol
Dofetilide
Procainamide
Flecainide
Macrolides
Fluoroquinolones
Methadone
Sumatriptan
Ondansetron
Cisapride

14.4 Appendix 4 – Childbearing Potential

Woman of Childbearing Potential

Women in the following categories are considered WOCBP (fertile):

1. Following menarche
2. From the time of menarche until becoming postmenopausal unless permanently sterile (see below)
    - A postmenopausal state is defined as no menses for ≥12 months without an alternative medical cause.
        - A high FSH level in the postmenopausal range will be used to confirm a postmenopausal state in women not using hormonal contraception or hormonal replacement therapy (HRT) with ≤12 months of amenorrhea.
        - Females on HRT and whose menopausal status is in doubt will be required to use one of the non-estrogen hormonal highly effective contraception methods if they wish to continue their HRT during the study. Otherwise, they must discontinue HRT to allow confirmation of postmenopausal status (i.e., ≥12 months of amenorrhea) before study enrollment.
    - Permanent sterilization methods (for the purpose of this study) include:
        - Documented hysterectomy
        - Documented bilateral salpingectomy
        - Documented bilateral oophorectomy

    For individuals with permanent infertility due to an alternate medical cause other than the above (e.g., Mullerian agenesis, androgen insensitivity, gonadal dysgenesis), Investigator discretion should be applied to determining study entry.

    Note: Documentation can come from the site personnel's review of the participant's medical records, medical examination or medical history review.

If fertility is unclear (e.g., amenorrhea in adolescents or athletes) and a menstrual cycle cannot be confirmed before the first administration of study drug, the participant will be considered as a WOCBP and will be required to follow the contraception guidelines.
