# Supplementary material for: Efficacy and Safety of the Neuroplastogen TSND-201 for the Treatment of PTSD: A Randomized Clinical Trial
Source: JAMA Psychiatry. 2026 Feb 18;83(5):469–77. doi: 10.1001/jamapsychiatry.2025.4625 (PMC12917749; doi:10.1001/jamapsychiatry.2025.4625)
Supplement: Supplement 2. — Statistical Analysis Plan. [file jamapsychiatry-e254625-s002.pdf]

# A Pilot Study to Assess the Use of Methylone in the Treatment of PTSD

## IMPACT-1 (Investigation of Methylone for Post-Traumatic Stress Disorder [PTSD])

## Protocol Number: TT-TSND-201

# STATISTICAL ANALYSIS PLAN FOR PART B

| | |
|---|---|
| **Sponsor:** | Transcend Therapeutics |
| **Clinical Phase** | 2 |
| **Statistical Analysis:** | TCM Groups, Inc. |
| **Version:** | V1.0 |
| **Version Date:** | March 6, 2025 |

### Confidentiality statement

The information contained in this document is confidential and shall not be disclosed by any person unless required by relevant laws and regulations. In any event, individuals who have access to the document shall understand its exclusivity and confidentiality and shall not disclose it to others. The above restrictions on the confidentiality of this document apply to documents that may be accessed in the future and are also marked proprietary or confidential.

# SIGNATURE PAGE - STATISTICAL ANALYSIS

I have read this Statistical Analysis Plan and agree to conduct statistical analysis in accordance with the content of the Statistical Analysis Plan.

[redacted]

# SIGNATURE PAGE - SPONSOR

I confirm and approve that the Statistical Analysis Plan is written in accordance with the clinical research protocol, relevant guidelines and regulations.

[redacted]

# TABLE OF CONTENTS


**SIGNATURE PAGE - STATISTICAL ANALYSIS** 2

**SIGNATURE PAGE - SPONSOR** 3

**TABLE OF CONTENTS** 4

**LIST OF ABBREVIATIONS** 6

**1 INTRODUCTION** 7

**2 STUDY OBJECTIVES** 7

**2.1 Primary Objective** 7

**2.2 Secondary Objectives** 7

[redacted]

**3 STUDY DESIGN** 7

**3.1 Study Design Overview** 7

**4 SAMPLE SIZE DETERMINATION** 8

**5 ANALYSIS SETS** 8

**5.1 Modified Intent-to-Treat (mITT) Population** 8

**5.2 Per-Protocol Population** 8

**5.3 Safety Population** 9

**6 ENDPOINTS AND COVARIATES** 9

**6.1 Efficacy Assessments** 9

**6.2 Safety Assessments** 13

**7 HANDLING OF MISSING VALUES** 14

**7.1 Efficacy Analysis** 14

**7.2 Safety Analysis** 15

**8 STATISTICAL ANALYSIS** 15

**8.1 General Aspects of the Statistical Analysis** 15

**8.2 Subject Information** 16

**8.3 Efficacy Analysis** 18

**8.4 Safety Analysis** 20

[redacted]

**9 DESCRIPTION OF STATISTICAL ANALYSIS PLAN** 23

**9.1 Changes from the Protocol Specified Statistical Analyses** 23

**10 REFERENCE** 24

**11 APPENDIX** 25

**11.1 Appendix 1 Scoring Instructions for the PSQI** 25

**11.2 Appendix 2 Mock Shell of Tables, Figures and Listings** 27

**11.3 Appendix 3 MMRM and CMH SAS Sample Code** 27

**12 REVISION RECORD ____ 28**

# LIST OF ABBREVIATIONS

| Abbreviations | Definition |
|---|---|
| [redacted] | [redacted] |
| AE | Adverse Event |
| AESI | Adverse event of special interest |
| ATC | Anatomical Therapeutic Chemical |
| BIQ | Blinding Integrity Questionnaire |
| BMI | Body mass index |
| BDI-II | Beck Depression Inventory-II |
| CAPS-5 | Clinician-Administered PTSD Scale for DSM-5 |
| CGI-I | Clinician Global Impression of Improvement |
| CGI-S | Clinical Global Impression of Severity |
| CMH | Cochran–Mantel–Haenszel |
| C-SSRS | Columbia Suicide Severity Rating Scale |
| CSR | Clinical study report |
| DBP | Diastolic blood pressure |
| DSMB | Data and Safety Monitoring Board |
| ECG | 12-lead electrocardiograms |
| HR | Heart rate |
| ICF | Informed consent form |
| ICH | The International Council for Harmonisation of Technical Requirements for Pharmaceuticals for Human Use |
| Max | Maximum |
| MADRS | Montgomery-Åsberg Depression Rating Scale |
| MedDRA | Medical Dictionary for Regulatory Activities |
| Min | Minimum |
| mITT | Modified Intent-to-Treat |
| [redacted] | [redacted] |
| MMRM | Mixed Model for Repeated Measures |
| PTGI | Post-traumatic Growth Inventory |
| PTSD | Post-traumatic stress disorder |
| PT | Preferred Term |
| PCL-5 | PTSD Checklist for DSM-5 |
| PGI-C | Patient Global Impression of Change |
| PGI-S | Patient Global Impression of Severity |
| PSQI | Pittsburgh Sleep Quality Index |
| QTcF | Fridericia corrected value of the interval between the Q and T waves |
| SAE | Serious adverse event |
| SAP | Statistical Analysis Plan |
| SBP | Systolic blood pressure |
| SD | Standard Deviation |
| SDS | Sheehan Disability Scale |
| SE | Standard Error |
| SOC | System Organ Class |
| TEAE | Treatment-emergent adverse event |
| WEMWBS | Warwick-Edinburgh Mental Wellbeing Scale |

# 1 INTRODUCTION

This statistical analysis plan (SAP) is based on the protocol TT-TSND-201 of Transcend Therapeutics, 'A Pilot Study to Assess the Use of Methylone in the Treatment of PTSD IMPACT-1 (Investigation of Methylone for Post-Traumatic Stress Disorder [PTSD])'. This SAP is used for Part B of protocol TT-TSND-201.

The purpose of this SAP is to outline in detail the statistical methods, data derivations, method for handling missing data, definition of analysis population and presentations of data. Specifications for tables, figures, and listings are contained in a separate document. Unless otherwise stated with reasoning, the methods discussed in this SAP are the ones outlined in the Protocol.

This SAP is written in compliance with the ICH E3 (Structure and Content of Clinical Study Reports) [1] and ICH E9 (Statistical Principles for Clinical Trials)[2] published by the International Coordination of Technical Requirements for the Registration of Human Drugs (ICH).

# 2 STUDY OBJECTIVES

## 2.1 Primary Objective

The primary objective of this study is to assess the efficacy of methylone in treating PTSD symptoms.

## 2.2 Secondary Objectives

The secondary objectives of this study are as follows:

- To assess the effect of methylone compared to placebo on sleep quality, functional disability, treatment satisfaction, quality of life, and physical function in participants with PTSD.
- To assess the safety and tolerability of oral methylone compared to placebo administered weekly over 4 weeks in participants with PTSD.

## [redacted] [redacted]

[redacted]

# 3 STUDY DESIGN

## 3.1 Study Design Overview

This is a two-part study to assess methylone for the management of the symptoms of PTSD. Part A was an open-label, non-controlled assessment in approximately 15 evaluable participants with PTSD to assess early safety and efficacy and to confirm procedures included in the blinded portion (Part B) of the study. After completion of Part A, enrollment for Part B began. Unless otherwise stated, Part B is identical to Part A with the exception of the inclusion of a placebo arm in Part B. This SAP covers the analysis of the Part B data.

Part B is a randomized, double-blind, parallel-group, placebo-controlled assessment of methylone for the management of the symptoms of PTSD in approximately 64 patients. This multi-center study will be conducted in up to 25 sites globally.

There are 4 planned dose sessions for each participant. Participants in Part B will be randomized 1:1 to the one of the two study treatment arms and will receive treatment at each of the weekly dose sessions for the duration of the study.

The two blinded study treatment arms for Part B are:

- Methylone 150 mg, with a booster administration of 100 mg administered 90 (±10) minutes after the initial administration, during each dose session.
- Matched placebo at each timepoint (initial + booster) during each dose session.

For each participant the study consists of:

- **Screening Period (Day -28 to Baseline)**: Informed consent, eligibility assessment, and enrolment of eligible participants.
- **Baseline/Preparatory Session (Day -4 to Day -1)**: Baseline assessments, confirmation of eligibility, and a preparatory psychoeducation session leading to enrolment confirmation.
- **Treatment Period (Day 1 to Day 24)**: Four weekly dose sessions. Each dose session is followed by a safety phone call 1 day after dosing and efficacy assessments 2 days after dosing.
- **Follow-Up Period (Day 29 to Day 64)**: Follow-Up visits for efficacy and safety will occur at 1, 2, 3 and 6 weeks post-final study drug administration..

The expected duration of participation for each participant who completes all study visits, from Screening to the EOS visit, is up to 16 weeks.

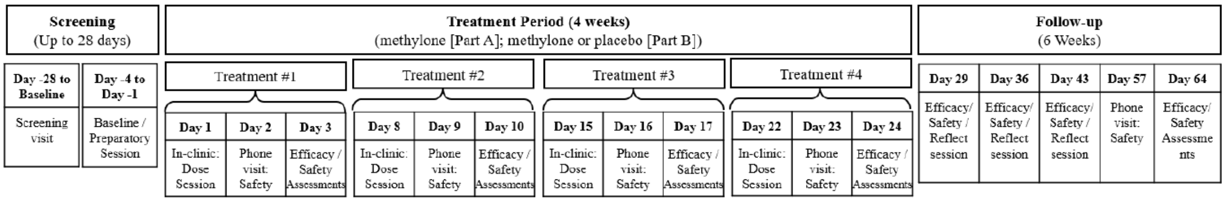


# 4 SAMPLE SIZE DETERMINATION

Sample size calculations for Part B were performed using PASS 2022 to determine the sample size required to detect a significant difference between CAPS-5 scores in the methylone and placebo groups (the efficacy endpoint). A previous phase 3 trial investigating the effects of MDMA versus placebo on CAPS-5 scores found a placebo-corrected treatment effect at the end of treatment of 11 points and a pooled standard deviation (SD) of 12 (Mitchell 2021). Assuming a treatment effect of 11 points and a pooled SD of 12 in this trial, with a sample size of 20 or 27 evaluable participants in each treatment group, the trial should provide 80% or 90% power, respectively. Assuming a dropout rate of 15%, a target sample size of 46 (23 per treatment group) or 64 (32 per treatment group) will provide 80% or 90% power, respectively.

# 5 ANALYSIS SETS

## 5.1 Modified Intent-to-Treat (mITT) Population

The mITT population includes all participants who are randomized, received at least 1 dose of study drug and have at least 1 valid post-baseline assessment. Participants are analyzed on the treatment to which they are randomized to receive.

## 5.2 Per-Protocol Population

The Per-Protocol population includes all participants in the mITT population who received at least one administration of study treatment and have no important protocol deviations.

### 5.3 Safety Population

The Safety population includes all participants who receive at least one administration of study treatment. Participants will be summarized according to treatment received. The Safety population will be used to analyze the safety endpoints.

# 6 ENDPOINTS AND COVARIATES

## 6.1 Efficacy Assessments

### 6.1.1 Clinician-Administered PTSD Scale for DSM-5 (CAPS-5)

The CAPS-5 is a 30-item structured interview that can be used to: Make a current diagnosis of PTSD; Assess change in PTSD symptoms over time.

In addition to assessing the 20 DSM-5 PTSD symptoms, questions target the onset and duration of symptoms, subjective distress, impact of symptoms on social and occupational functioning, improvement in symptoms since a previous CAPS administration, overall response validity, overall PTSD severity and specifications for the dissociative subtype (depersonalization and derealization). The CAPS-5 also rates intrusion symptoms (intrusive thoughts or memories), avoidance, cognitive and mood symptoms, arousal and reactivity symptoms, duration and degree of distress.

The CAPS-5 total severity score (primary efficacy measure) is calculated by summing severity scores for items 1-20. CAPS-5 severity scores range from 0-80 points, with higher scores indicating greater PTSD symptom severity.

The CAPS-5 symptom cluster severity scores are calculated by summing the individual item severity scores for symptoms contained in a given DSM-5 cluster. Thus, the Criterion B (intrusion) severity score is the sum of the individual severity scores for items 1-5; the Criterion C (avoidance) severity score is the sum of items 6 and 7; the Criterion D (cognitions and mood) severity score is the sum of items 8-14; and the Criterion E (arousal and reactivity) severity score is the sum of items 15-20. A symptom cluster score may also be calculated for dissociation by summing items 29 and 30.

PTSD diagnostic status is determined on the CAPS-5 by first dichotomizing individual symptoms as “present” or “absent” then following the DSM-5 diagnostic rule. A symptom is considered present only if the corresponding item severity score is rated 2=Moderate/threshold or higher otherwise a symptom is considered absent. The DSM-5 diagnostic rule requires the presence of least one Criterion B symptom, one Criterion C symptom, two Criterion D symptoms, and two Criterion E symptoms. Criterion G requires a rating of 2=moderate or higher on items 23-25.

### 6.1.2 Structured Interview Guide for the Montgomery-Åsberg Depression Rating Scale (MADRS) (SIGMA)

The SIGMA is a standardized version of the MADRS. The SIGMA maintains the original format of the MADRS, which is a 10-item clinician-rated, diagnostic questionnaire used to measure depression severity, with the exception that the first two items are reversed. Each item is measured on a 7-point scale, from 0 to 6. Higher total MADRS scores indicate more severe depression. Follow-up questions are also provided to clarify symptoms, if required.

Total MADRS score ranges from 0-60 points, with higher total scores indicating more severe depression.

### 6.1.3 Clinician Global Impressions Scale – Severity (CGI-S)

The CGI-S is designed to acquaint the participant’s severity of symptoms with those of other people experiencing the same mental ailment. The CGI-S rates this severity on a 7-point scale,

with (1) representing normal symptoms, meaning the participant is not ill, and (7) representing participants among the most severely ill. The rating (4) represents a participant that is defined as a participant that is defined as moderately ill.

### 6.1.4 Sheehan Disability Scale (SDS)

The SDS is a brief, 5-item self-report tool that assesses functional impairment in work, social life/leisure activities and family life/home responsibilities. The SDS is designed to measure the extent to which the three major domains in the participant's life are functionally impaired by psychiatric or medical symptoms. Each domain is rated from 0 to 10, with higher scores representing more functional impairment. The total score is the mean of the domains.

In addition, the SDS also collects information on the number of days lost and underproductive during the past week.

### 6.1.5 PTSD Checklist for DSM-5 (PCL-5)

The PCL-5 is a 20 item self-report measure that assesses the presence and severity of the 20 DSM-5 symptoms of PTSD. Participants rate each item from 0 ('not at all') to 4 ('extremely') to indicate the degree to which they have been affected by that particular symptom over the past week.

A total symptom severity score ranges from 0 to 80, with higher scores representing more severe symptoms.

### 6.1.6 Patient Global Impression of Severity (PGI-S)

The PGI-S is a self-report tool to measure disease severity. The PGI-S is a 5-point scale, ranging from '1 – None', through to '5 – Very severe'.

### 6.1.7 Beck Depression Inventory-II (BDI-II)

The BDI-II is a 21-item self-report measure assessing depressive symptoms and their severity. Items include hopelessness and irritability, cognitions such as guilt or feelings of being punished, as well as physical symptoms such as fatigue and weight loss. Each item is rated on a 4-point scale, from 0 to 3. Items are summed to create a total score.

A total score ranging from 0 to 63 is possible, with higher scores indicating higher levels of depression.

### 6.1.8 Warwick-Edinburgh Mental Wellbeing Scale (WEMWBS)

The WEMWBS is a scale of 14 positively worded items for assessing a population's mental wellbeing. The items cover both physiological functioning aspects of mental wellbeing (including: optimism, autonomy, agency, curiosity, clarity of thought and positive relationships) and positive effect (feelings, including: confidence, feeling relaxed, cheerful, having the energy to spare). The scale has 5 response categories, ranging from 1 "none of the time" to 5 "all of the time", summed to provide a single score.

A total score ranging from 14 to 70 is possible, with higher scores indicating higher levels of mental wellbeing.

### 6.1.9 Pittsburgh Sleep Quality Index (PSQI)

The PSQI is a measure of self-reported sleep quality and sleep disturbance. The PSQI contains 19 self-rated questions, which are combined to form 7 'component' scores, each of which has a range of 0 to 3 points. The 7 components of the PSQI are: subjective sleep quality, sleep latency, sleep duration, habitual sleep efficiency, sleep disturbances, use of sleeping medications and daytime dysfunction.

The sleep component scores are summed to yield a total score ranging from 0 to 21, with a higher total score indicating worse sleep quality.

See Appendix 1 Scoring Instructions for the PSQI.

### 6.1.10 Patient Global Impression of Change (PGI-C)

The PGI-C is a self-report tool to reflect a participant's belief about the efficacy of treatment. The PGI-C is a 7-point scale depicting a participant's rating of overall improvement. Participants rate their change from '1 – Very much improved' through to '7 – Very much worse'.

### 6.1.11 Clinician Global Impressions Scale – Improvement (CGI-I)

The CGI-I is a 7-point scale that requires the assessor to assess how much the participant's illness has improved or worsened relative to a baseline state prior to dosing. The CGI-I scale rates improvement with (1) representing a 'very much improved' participant to (7) representing a participant who has become 'very much worse' due to treatment. The rating (4) represents a participant displaying no change from the treatment.

### 6.1.12 Post-Traumatic Growth Inventory (PTGI)

The Post-Traumatic Growth Inventory measures the extent to which survivors of traumatic events perceive personal benefits, including changes in perceptions of self, relationships with others and philosophy of life, accruing from their attempts to cope with trauma and its aftermath.

This 21-item scale includes factors of: new possibilities, relating to others, personal strength, spiritual change and appreciation of life.

The PTGI is scored by adding the individual responses (ranging from 0 "I did not experience this change as a result of my crisis" to 5 "I experienced this change to a very great degree as a result of my crisis"). Total scores range from 0 to 105, with higher scores indicating that the person has undergone a positive transformation. Individual factors are scored by adding responses to items on each factor.

| Factor | Item Numbers |
|---|---|
| Factor I: Relating to Others (0 to 35) | Q6; Q8; Q9; Q15; Q16; Q20; Q21 |
| Factor II: New Possibilities (0 to 25) | Q3; Q7; Q11; Q14; Q17 |
| Factor III: Personal Strength (0 to 20) | Q4; Q10; Q12; Q19 |
| Factor IV: Spiritual Change (0 to 10) | Q5; Q18 |
| Factor V: Appreciation of Life (0 to 15) | Q1; Q2; Q13 |

[redacted]

[redacted]

[redacted]

[redacted]

#### 6.1.15 Blinding Integrity Questionnaire (BIQ)

The BIQ is a visual analogue scale that will be used to ascertain the degree of blinding perceived by the participant; participants will be asked to circle a number from 0 (strongly believe they

received Placebo) to 10 (strongly believe they received active drug) indicating which treatment they believed they were administered during the dose sessions.

## 6.2 Safety Assessments

### 6.2.1 Adverse events

#### 6.2.1.1 Adverse Event

An AE is any untoward medical occurrence in a study participant which either emerges, or worsens from enrolment/randomization, during the clinical study, regardless of its potential relationship to the medicinal product. An AE, therefore, can be any unfavorable or unintended sign, including a clinically significant abnormal laboratory finding, symptom, or disease temporally, whether or not it is considered to be study drug-related.

#### 6.2.1.2 Serious Adverse Events

A SAE is any untoward medical occurrence that, at any dose:

- Results in death
- Is life-threatening
- Requires hospitalization or prolongation of existing hospitalization
- Results in persistent or significant disability or incapacity
- Is a congenital abnormality or birth defect
- Is an important medical event

#### 6.2.1.3 Adverse Events of Special Interest

The Sponsor will pay special attention to a subset of AEs; these AEs will be marked in the eCRF with the denotation AESIs whether serious or non-serious. Adverse events of special interest (AESI) include:

- Grade 3 hypertension, SBP ⩾160 mmHg or DBP ⩾100 mmHg, requiring the need for medical intervention with more than one drug or more intensive therapy than previously used.
- Significant cardiovascular events
- AEs related to suicide risk, such as; suicides, suicide attempts, self-injurious behavior associated with suicidal ideation and suicidal ideation judged to be serious or severe in the opinion of the Investigator.
- Signals of abuse potential AE involving terms of behavioral addiction, drug abuser, substance abuser.

### 6.2.2 12-Lead Electrocardiogram

The 12-lead ECG will be collected as described in the protocol. The following ECG parameters are calculated: HR and PR, QRS, QT and QTcF intervals (if not available, QTcF = QT/(RR^0.33)).

### 6.2.3 Vital Signs

Supine vital signs, including HR, BP, and temperature are collected during this study at the intervals described in the protocol.

### 6.2.4 Physical Examination

A physical examination, including body weight (Screening and EOS) and height (Screening only), and assessments of the head, eyes, ears, nose, throat, skin, neurological, lungs, cardiovascular system, abdomen, and lymph nodes will be conducted.

### 6.2.5 Clinical Laboratory Evaluations

All blood and urine analyses will be conducted on-site or at a central laboratory at the sampling times indicated in the Schedule of Assessments. For abnormal values, additional testing may be performed, or clinical laboratory evaluations may be added to evaluate the abnormal values.

### 6.2.6 Columbia-Suicide Severity Rating Scale (C-SSRS)

The C-SSRS is a semi-structured interview designed to assess the severity and intensity of suicidal ideation, suicidal behavior and non-suicidal self-injurious behavior over a specified time period. The measurement of suicidal ideation is based on five 'yes' or 'no' questions with accompanying descriptions arranged in order of increasing severity. If the participant answers 'yes' to either questions 1 or 2, the intensity of ideation is assessed in five additional questions related to frequency, duration, controllability, deterrents and reasons for the most severe suicidal ideation. Suicidal behavior is assessed by asking questions categorizing behaviors into actual, aborted and interrupted attempts; preparatory behavior, and non-suicidal self-injurious behavior.

The C-SSRS is made up of ten categories, all of which maintain binary responses (yes/no) to indicate a presence or absence of ideation (Category 1-5) or behavior (Category 6-10). The ten categories included in the C-SSRS are as follows:

- Category 1 – Wish to be Dead;
- Category 2 – Non - specific Active Suicidal Thoughts;
- Category 3 – Active Suicidal Ideation with Any Methods (Not Plan) without Intent to Act;
- Category 4 – Active Suicidal Ideation with Some Intent to Act, without Specific Plan;
- Category 5 – Active Suicidal Ideation with Specific Plan and Intent;
- Category 6 – Preparatory Acts or Behavior;
- Category 7 – Aborted Attempt;
- Category 8 – Interrupted Attempt;
- Category 9 – Actual Attempt (non-fatal);
- Category 10 – Completed Suicide.

A yes/no binary response is also utilized in assessing Self - injurious Behavior without Suicidal Intent.

C-SSRS Suicidal ideation scores is a numerical score derived from the C-SSRS categories (the maximum suicidal ideation category (1-5 on the CSSRS) present at the assessment. A score of 0 is assigned if no ideation is present.). The score is created at each assessment for each participant and is used for determining treatment emergence. A C-SSRS ideation score of 4 or 5 is termed 'serious ideation', > 0 is termed 'positive ideation'.

Suicidal ideation: A "yes" answer at any time from first administration of study drug to any one of the five suicidal ideation questions (Categories 1-5) on the C-SSRS. Suicidal behavior: A "yes" answer at any time from first administration of study drug to any one of the five suicidal behavior questions (Categories 6-10) on the C-SSRS. Suicidal ideation or behavior: A "yes" answer at any time from first administration of study drug to any one of the ten suicidal ideation and behavior questions (Categories 1-10) on the C-SSRS.

# 7 HANDLING OF MISSING VALUES

## 7.1 Efficacy Analysis

The efficacy analysis will be conducted using the available data without any statistical imputation for missing data for data lost to follow up or otherwise unavailable. As for scales, if any item is

missing, any total or sum involving that item will be considered missing. As SDS total score is calculated as a mean of the domains, up to 1 of 3 questions can be missing per scale design.

### 7.1.1 For Disease Characteristics

For baseline disease characteristics (eg, age at end date of index trauma, time since end date of index trauma), if month and day are missing, July 1 will be imputed; if only the day is missing, the 15th will be imputed.

## 7.2 Safety Analysis

Unless specified below, safety analyses will be based on the observed data.

### 7.2.1 For Adverse Events Onset Dates

If onset date is completely missing, onset date is set to the first dose.

If (year is present and month and day are missing) or (year and day are present and month is missing):

- If year = year of the first dose, then set month and day to month and day of the first dose
- If year < year of the first dose, then set month and day to December 31.
- If year > year of the first dose, then set month and day to January 1.

If month and year are present and day is missing:

- If year = year of the first dose and

  If month = month of the first dose then set day to day of first dose

  If month < month of first dose then set day to last day of month

  If month > month of first dose then set day to first day of month
- If year < year of first dose then set day to last day of month
- If year > year of first dose then set day to first day of month

For all other cases, set onset date to the first dose.

### 7.2.2 For Concomitant Medications and Procedures/Therapies Missing Dates

Start Date:

- If (year is present and month and day are missing) or (year and day are present and month is missing) then set month and day to January 1.
- If year and month are present and day is missing then set day to first day of month.

End Date:

- If (year is present and month and day are missing) or (year and day are present and month is missing) then set month and day to December 31.
- If year and month are present and day is missing then set day to last day of the month.

# 8 STATISTICAL ANALYSIS

## 8.1 General Aspects of the Statistical Analysis

Treatments will be labeled as follows in data summaries for Part B:

- TSND-201
- Placebo

- Total (if needed)

#### 8.1.1 General Rules

Continuous variables will be summarized using the following descriptive summary statistics: the number of subjects (n), arithmetic mean, standard deviation (SD), standard error (SE), median, minimum value (Min), and maximum value (Max). The decimal places of Mean and Median will be formatted to one more than the original value. The decimal places of SD and SE will be formatted for two more than the original value. The decimal places of Min and Max will be formatted with the same as original value. The number of decimal places cannot exceed 4.

Frequencies (n) and percentage (%) will be used to summarize categorical data, and percentage is formatted for one decimal place. If the percentage is 0, then percentage is set to null. If the percentage is 100, then it is formatted as 100.0.

The derived data will reserve one more decimal place than the original. Descriptive statistics of derived data follow the above rules.

#### 8.1.2 Software

SAS 9.4 or higher is used for statistical analysis.

#### 8.1.3 Definition of Baseline

Baseline values will be defined as the worst measurement prior to the initiation of study treatment (or last non-missing if only one measurement was taken), unless specified otherwise for the corresponding endpoint.

#### 8.1.4 Unscheduled Visits

Subject data obtained during unscheduled visits/assessments will not be summarized, but will be included in subject data listings only.

#### 8.1.5 Multiplicity

Findings from this study will be used to design and power future clinical trials to further evaluate the efficacy and safety of methylone for the treatment of PTSD.

As this trial is not a confirmatory study, multiplicity is not applicable.

#### 8.1.6 Tests

All tests will be based on one-sided significant level of 0.05. In addition to the tests, two-sided 90% confidence intervals will be reported.

#### 8.1.7 Covariates

For the analysis of select efficacy endpoints, the baseline value will be included (if applicable) as a covariate. Sex will also be used as a covariate.

### 8.2 Subject Information

#### 8.2.1 Subject Disposition

Subject disposition will be tabulated with the following numbers (%) of subjects: total screened, screen failure (along with screen failure primary reason), randomized, study drug treated, completed study drug (early discontinued from study drug, along with primary reasons), completed study (discontinued from study, along with primary reasons).

The number of subjects in each analysis populations (mITT Population, Per-protocol Population, and Safety Population) will be reported.

Data listings will include subject disposition and subjects included in each analysis population.

#### 8.2.2 Protocol Deviations

Important protocol deviations will be classified and documented before analysis and will be summarized in terms of frequency and percentage by category.

All protocol deviations will be presented in a data listing.

#### 8.2.3 Demographic and Baseline Characteristics

##### 8.2.3.1 Demographic Data

Age, sex, race, ethnicity, height, weight, body mass index (BMI), and childbearing potential will be summarized and listed.

##### 8.2.3.2 Baseline Characteristics

Summary of duration of PTSD symptoms (in years, from CAPS-5 conducted at screening), age at end date of index trauma, time since end date of index trauma (years = (ICF date-end date of index trauma+1)/365.25), type of traumatic event (PT MedDRA coding of index event), prior PTSD treatment type, prior psychedelics used, and dissociative PTSD symptoms (Yes = Item 29 or 30 have a severity$\geqslant$2) will be summarized.

Lifetime C-SSRS (Lifetime accounts for all suicidal ideation and behavior prior to the study): positive lifetime suicidal ideation (ideation score >0), serious lifetime suicidal ideation (ideation score of 4 or 5), positive lifetime suicidal behavior (behavior score >0) will be summarized. The same summary for past 2 months suicidal ideation and past 5 years suicidal behavior will be presented.

Data listings will include the information of PTSD duration and other baseline characteristics.

##### 8.2.3.3 Medical History

Medical history will be coded by MedDRA (Medical Dictionary for Regulatory Activities, version 25.1 or higher). Medical history will be summarized according to Preferred Term (PT).

Data listings will include the medical history of subjects.

#### 8.2.4 Prior and Concomitant Medication and Procedures/Therapies

Medications will be coded by WHO Drug Dictionary (version Sep 2022 or higher). Medication is summarized by incidence rate according to Anatomical Therapeutic Chemical 4 (ATC4) and Preferred Name. Procedures/therapies will be coded by MedDRA (version 25.1 or higher), and summarized by incidence rate according to PT.

Prior medication/procedure is any medication that started and stopped before initial dosing of study drug. Concomitant medication/procedure is any medication/procedure received at or after the dose of study drug, medication/procedure that was received before initial dosing and continued after initial dosing of study drug, or medication/procedure with a missing stop date. If a medication/procedure has a missing or partial missing start/end date and it cannot be determined whether it was taken before initial dosing or concomitantly, it will be considered as concomitant.

Prior and concomitant medication and procedures will be listed separately.

#### 8.2.5 Treatment Exposure and Compliance

The number of doses administered by dosing session will be summarized.

Drug administration information will be listed by subject.

## 8.3 Efficacy Analysis

These analyses will be based on the mITT Population. All efficacy endpoint data will be listed for individual participants.

### 8.3.1 Statistical Methods

#### 8.3.1.1 Analyses for Continuous Efficacy End Points

A Mixed Model for Repeated Measures (MMRM) will be fitted for select efficacy endpoints to analyze the difference between treatments. The model will include treatment, visit, and treatment by visit interaction as fixed effects, where visit is the repeated effect. The endpoints baseline value (if applicable) and sex will be included as covariates.

The covariance structure will be modeled using an "unstructured" (UN). Other covariance structures may be considered if the UN model dose not converge, such as AR(1), and CS. The visit-by-treatment interaction allows for potential differences in the treatment effect at different time points. Pairwise contrasts will be constructed to compare the methylone group to the placebo group at each visit specifically.

Besides, summary statistics will be provided for the actual values and for the change from baseline.

#### 8.3.1.2 Analyses for Categorical Efficacy End Points

Frequency tables and the Cochran–Mantel–Haenszel (CMH) test will be applied to the actual value of each visit. The descriptive statistics (n and percentage) for the treatment arms, and difference between treatment arms as well as the two-sided confidence interval of the treatment difference will be presented. The confidence interval will be constructed based on normal approximation.

See Appendix 3 MMRM and CMH SAS Sample CodeAppendix 3 MMRM and CMH SAS Sample Code.

### 8.3.2 Efficacy Endpoints

#### 8.3.2.1 Primary Endpoint

The primary efficacy endpoint is the CAPS-5 total severity score change from baseline as compared to placebo. Mean change from baseline compared with placebo in CAPS-5 total severity score using MMRM.

#### 8.3.2.2 Secondary Endpoint

##### 8.3.2.2.1 CAPS-5

The CAPS-5 total severity score (absolute, change from baseline) will also be summarized by visit.

Summary percentage of participants by visit having

- Treatment response, defined as:
  - ⩾10 point reduction on the CAPS-5 from baseline
  - ⩾30% improvement from baseline on CAPS-5
  - ⩾50% improvement from baseline on CAPS-5
- Remission, defined as:
  - A score of ≤ 11 on the CAPS-5

##### 8.3.2.2.2 MADRS

Mean change from baseline compared with placebo in MADRS total score using MMRM.

The MADRS total score (absolute, change from baseline) will also be summarized by visit.

Summary percentage of participants by visit having

- Treatment response, defined as:
  - ⩾50% improvement from baseline on the MADRS
- Remission, defined as:
  - A score of ≤ 10 on the MADRS

**8.3.2.2.3 SDS**

Mean change from baseline compared with placebo in mean SDS total score using MMRM.

The mean SDS total score and each domain (absolute, change from baseline) will be summarized by visit. The number of days lost and days underproductive will also be summarized by visit.

**8.3.2.2.4 PCL-5**

Mean change from baseline compared with placebo in PCL-5 score using MMRM.

Besides, the PCL-5 score (absolute, change from baseline) will also be summarized by visit.

**8.3.2.2.5 CGI-S**

Mean change from baseline compared with placebo in CGI-S using MMRM.

Besides, the CGI-S (absolute, change from baseline) will also be summarized by visit.

The CGI-S will also be summarized by N (%) of the categorical values (i.e., 1, 2, 3, 4, 5, 6, 7).

**8.3.2.2.6 PGI-S**

Mean change from baseline compared with placebo in PGI-S using MMRM.

Besides, the PGI-S (absolute, change from baseline) will also be summarized by visit.

The PGI-S will also be summarized by N (%) of the categorical values (i.e., 1, 2, 3, 4, 5).

**8.3.2.2.7 BDI-II**

Mean change from baseline compared with placebo in BDI-II score using MMRM.

Besides, the BDI-II score (absolute, change from baseline) will also be summarized by visit.

**8.3.2.2.8 WEMWBS**

Mean change from baseline compared with placebo in WEMWBS score using MMRM.

Besides, the WEMWBS score (absolute, change from baseline) will also be summarized by visit.

**8.3.2.2.9 PSQI**

Mean change from baseline compared with placebo in PSQI score using MMRM.

Besides, the PSQI score (absolute, change from baseline) will also be summarized by visit.

**8.3.2.2.10 PGI-C**

The number and percentage of each level the PGI-C will be summarized.

Improved items include: “Very much improved”, “Much improved”, “Slightly improved”.

The number and percentage of improved rate on the PGI-C will be summarized and compared between treatment groups using CMH test.

**8.3.2.2.11 CGI-I**

Improved items include: “Very much improved”, “Much improved”, “Minimally improved”.

The number and percentage of improved rate on the CGI-I will be summarized and compared between treatment groups using CMH test.

### 8.4 Safety Analysis

All safety analyses will be performed on the Safety Population. No formal statistical analyses of safety analysis were planned.

Safety assessments will include clinical laboratory evaluations (clinical chemistry, hematology and urinalysis), vital signs (HR, BP and body temperature), 12-lead ECG, physical examinations, C-SSRS and AE monitoring.

To assess the safety and tolerability of oral methylone compared to placebo administered weekly over 4 weeks in participants with PTSD:

- Incidence and severity of TEAEs
- Incidence and severity of AESIs
- Change in HR, SBP, DBP and temperature during each dosing session
- Clinically significant changes in ECG
- Changes from baseline in clinical laboratory parameters (clinical chemistry, hematology, and urinalysis)

#### 8.4.1 Adverse Events

Treatment-emergent AEs (TEAE) are defined as any AE with an onset date on or after the date of first administration of study treatment and not more than 7 days after the last dose unless otherwise suspected to be related to study drug. For AEs with missing or partial start dates, if there is no clear evidence that the AEs started before or after study treatment, the AEs will be classified as TEAEs. Suspected to be drug related TEAEs include Relationship to study drug: “Possibly Related”, “Probably Related”.

An overall summary of the number and percentage of subjects with below will be presented:

- Any TEAEs
- serious TEAEs
- TEAEs related to study drug
- serious TEAEs related to study drug
- TEAEs leading to study discontinuation
- TEAEs leading to drug withdrawn
- TEAEs leading to death
- TEAEs of special interest.

Also, the TEAEs will be presented by System Organ Class (SOC), and PT. Subjects will be counted only once for each SOC and PT.

In addition, subjects with TEAEs in various severity categories will be presented based on the maximum severity of a specific TEAE for a subject. Similarly, subjects with TEAEs in various relationship categories will be presented based on the most related relationship (from ‘not related’, ‘unlikely related’, ‘possibly related’, to ‘probably related’) of a specific TEAE for a subject.

All AEs will be presented in individual by-subject data listings. Listings of any subject experiencing TEAEs related to study drug, AESI and an SAE will be provided separately.

#### 8.4.2 Laboratory Tests

Clinical laboratory data will be summarized using descriptive statistics for the measured values and change from baseline for each parameter. The shift table (high, normal, low) is used to describe the clinical evaluation changes of laboratory tests prior and post the first dose.

Data listings will include the results of clinical laboratory evaluation, drug screen use, and pregnancy test.

#### 8.4.3 Vital Signs

**Per dosing session:** Baseline is defined as the measurements of pre-initial dose during each dosing session.

**Per participant:** Baseline is defined as the measurements collected at Visit 2. Post-baseline vitals were collected at Visit 15.

Where multiple values are recorded at a time for a participant, the mean of the values will be used in the descriptive statistics.

Descriptive statistics for the measured values and change from baseline will be calculated and plotted for each parameter by treatment and timepoint.

Vital signs (SBP, DBP, HR, respiratory rate and body temperature) will be listed for individual participants.

#### 8.4.4 12-Lead ECG

**Per dosing session:** Baseline is defined as the measurements of pre-initial dose during each dosing session.

**Per participant:** Baseline is defined as the measurements collected at Visit 1. Post-baseline ECG was collected at Visit 15.

Where multiple values (e.g. triplicate ECG) are recorded at a time for a participant, the mean of the values will be used in the descriptive statistics.

Descriptive statistics for the actual values and changes from baseline will be calculated for each parameter by treatment and timepoint. The shift table (clinical significance) is used to describe the changes of 12-Lead ECG results prior and post dose at each timepoint.

Standard 12-lead ECG parameters (PR, QRS, QT, QTcF intervals, and HR) will be listed for individual participants.

#### 8.4.5 Physical Examination

Shift table (clinical significance) is used to describe the changes of physical examination prior and post dose visit. Abnormal findings of physical examination data will be listed by participant.

#### 8.4.6 C-SSRS

The number and percentage of subjects with suicidal ideation (Category 1-5) or suicidal behavior (Category 6-10), and Self-injurious behavior without suicidal intent based on the C-SSRS after first administration of study drug will be summarized.

Two shift tables will demonstrate changes in C-SSRS, most severe suicidal ideation (0 to 5), and C-SSRS categories (No suicidal ideation or behavior, suicidal ideation, suicidal behavior) from baseline to post-treatment.

C-SSRS results will be listed for individual participants.

[redacted]

[redacted]

[redacted]

[redacted]

[redacted]

# 9 DESCRIPTION OF STATISTICAL ANALYSIS PLAN

This SAP describes the analysis for Part B of the IMPACT-1 study. The planned analyses identified in this SAP may be included in regulatory submissions and/or future manuscripts. Exploratory analyses, not identified in this SAP, may be performed to support the clinical development program. Any post-hoc or unplanned analyses that are performed but not identified in this SAP will be clearly identified in the clinical study report (CSR).

## 9.1 Changes from the Protocol Specified Statistical Analyses

This SAP is drafted on the basis of the content in the protocol.

There are some changes from the protocol:

1. This SAP includes additional derivative analysis of the CAPS-5 and MADRS scores, which are not directly described in the current protocol.
2. The SAP Section 7 added missing data or date imputation methods.
3. Protocol Section 9.5.1.5: Physical examination data will be listed by participant for abnormal findings only. In the SAP, Section 10.3.5: Shift table (clinical significance) is used to describe the changes of physical examination prior and post dose.
4. SDS in protocol is “a total score from 0 to 30, where 0-Unimpaired and 30-Highly impaired.” In the SAP, mean of each of the 3 domains (0-10) has been used.
5. The definition of ITT population has been revised to mITT population.
6. All tests will be based on “one-sided significant level of 0.05” instead of “two-sided significant level of 0.05”.
7. Add sex as a covariate. The endpoints baseline (if applicable) and sex will be as covariates in the analysis model.

In consideration of unpredictable changes of final data collection in the clinical trial, there may be minor adjustments to this SAP.

# 10 REFERENCE

[1] International Conference on Harmonization of Technical Requirements for Registration of Pharmaceuticals for Human Use, ICH Harmonised Tripartite Guideline, Structure and Content of Clinical Study Reports (E3), 30 November 1995.
[2] International Conference on Harmonisation of Technical Requirements for Registration of Pharmaceuticals for Human Use, ICH Harmonised Tripartite Guideline, Statistical Principles for Clinical Trials (E9), 5 February 1998.

# 11 APPENDIX

## 11.1 Appendix 1 Scoring Instructions for the PSQI

The PSQI contains 19 self-rated questions. Only self-rated questions are included in the scoring. The 19 self-rated items are combined to form seven component scores, each of which has a range of 0-3 points. In all cases, a score of 0 indicates no difficulty, while a score of 3 indicates severe difficulty. The seven component scores are then added to yield one global score, with a range of 0-21 points, 0 indicating no difficulty and 21 indicating severe difficulties in all areas.

- **Component 1: Subjective sleep quality**

Question #6, and assign scores as follows:

| Response | Component 1 score |
|---|---|
| Very good | 0 |
| Fairly good | 1 |
| Fairly bad | 2 |
| Very bad | 3 |

Component 1 score:_____

- **Component 2: Sleep latency**
  1. Question #2, and assign scores as follows:

| Response | Score |
|---|---|
| ≤15 minutes | 0 |
| 16-30 minutes | 1 |
| 31-60 minutes | 2 |
| ＞ 60minutes | 3 |

  2. Question #5a, and assign scores as follows:

| Response | Score |
|---|---|
| Not during the past month | 0 |
| Less than once a week | 1 |
| Once or twice a week | 2 |
| Three or more times a week | 3 |

  3. Add #2 score and #5a score
  4. Assign component 2 scores as follows

| Sum of #2 and #5a | Component 2 Score |
|---|---|
| 0 | 0 |
| 1-2 | 1 |
| 3-4 | 2 |
| 5-6 | 3 |

Component 2 score:_____

- **Component 3: Sleep duration**

  Question #4, and assign scores as follows:

| Response | Component 3 Score |
|---|---|
| >7 hours | 0 |
| 6-7 hours | 1 |
| 5-6 hours | 2 |
| <5 hours | 3 |

Component 3 score:______

- **Component 4: Habitual sleep efficiency**
  1. Write the number of hours slept (question #4) here:
  2. Calculate the number of hours spent in bed:
     Number of hours spent in bed: Getting up time (question #3)-Bedtime (questions #1)
  3. Calculate habitual sleep efficiency as follows:
     Habitual sleep efficiency (%) = (Number of hours slept/Number of hours spent in bed)*100
  4. Assign component 4 scores as follows:

| Habitual sleep efficiency (%) | Component 4 Score |
|---|---|
| >85 | 0 |
| 75-84 | 1 |
| 65-74 | 2 |
| <65 | 3 |

Component 4 score:______

- **Component 5: Sleep disturbances**
  1. Question #5b-5j, and assign scores for each question as follows:

| Response | Score |
|---|---|
| Not during the past month | 0 |
| Less than once a week | 1 |
| Once or twice a week | 2 |
| Three or more tiems a week | 3 |

  2. Sum of #5b-5j: Add the scores for questions #5b-5j
  3. Assign component 5 score as follows:

| Sum of # 5b-5j | Component 5 Score |
|---|---|
| 0 | 0 |
| 1-9 | 1 |
| 10-18 | 2 |
| 19-27 | 3 |

Component 5 score:______

- **Component 6: Use of sleeping medication**
  Question #7 and assign scores as follows:

| Response | Component 6 Score |
|---|---|
| Not during the past month | 0 |
| Less than once a week | 1 |
| Once or twice a week | 2 |
| Three or more times a week | 3 |

Component 6 score:_____

- **Component 7: Daytime dysfunction**
  1. Question #8, and assign scores as follows:

| Response | Score |
|---|---|
| Never | 0 |
| Once or twice | 1 |
| Once or twice each week | 2 |
| Three or more times each week | 3 |

2. Questions #9, and assign scores as follows:

| **Response** | **Score** |
|---|---|
| No problem at all | 0 |
| Only a very slight problem | 1 |
| Somewhat of a problem | 2 |
| A very big problem | 3 |

3. Sum of #8 and #9: Add the scores for question #8 and #9
4. Assign component 7 scores as follows

| **Sum of #8 and #9** | **Score** |
|---|---|
| 0 | 0 |
| 1-2 | 1 |
| 3-4 | 2 |
| 5-6 | 3 |

Component 7 score:_____

- **Global PSQI Score**: Add the seven component scores together.

## 11.2 Appendix 2 Mock Shell of Tables, Figures and Listings

The information and explanatory notes to be provided in the footnote or bottom of each table, figure and listing will include the following information: date and time of output generation; SAS program name; any other output specific details (for example, definition of abbreviations) that require further elaboration.

Some of the TFLs may not have sufficient numbers of subjects or data for presentation. If this occurs, the blank TFL shell will be presented with a message printed in the table, such as, “No data available for this report”.

The treatment and subject number will be included in all data listings. All listings will be sorted by treatment group, subject number, parameter and date, as applicable.

See separate file for mock shell of tables, figures and listings.

## 11.3 Appendix 3 MMRM and CMH SAS Sample Code

This MMRM approach can be implemented in SAS by a code similar to the following:

```
proc mixed data = adef method=REML;
    class Subjid trtpn avisitn;
    model Chg=trtpn avisitn trtpn*avisitn base sex/ddfm=kr; /* The baseline value will be included (if applicable) as a covariate.*/
    repeated avisitn/ subject = Subjid type=un;
    lsmeans trtpn trtpn*avisitn/diff cl;
    ods output Tests3=b1 LSMeans=b2 Diffs=b3;
run;
```

This CMH approach can be implemented in SAS by a code similar to the following:

```
Proc freq data=adef;
    Tables trtpn*outcome/cmh riskdiff;
    Weight count;
Run;
```

# 12 REVISION RECORD

| Version | Version Date | Author | Modify Reason |
|---|---|---|---|
| V1.0 | March 6, 2025 | Sean Wang | Original version |
