# Supplementary material for: Efficacy and Safety of the Neuroplastogen TSND-201 for the Treatment of PTSD: A Randomized Clinical Trial
Source: JAMA Psychiatry. 2026 Feb 18;83(5):469–77. doi: 10.1001/jamapsychiatry.2025.4625 (PMC12917749; doi:10.1001/jamapsychiatry.2025.4625)
Supplement: Supplement 3. — eAppendix. Part A of IMPACT-1 (Open-Label) Overview and Results eTable 1. Demographics and Baseline Characteristics (IMPACT-1, Part A) eFigure 1. CAPS-5 Change from Baseline Over Time (IMPACT-1, Part A) eFigure 2. Response and Remission on CAPS-5 at Day 64 (IMPACT-1, Part A) eTable 2. Treatment-Emergent AEs Occurring in >1 Participant (IMPACT-1, Part A) [file jamapsychiatry-e254625-s003.pdf]

Supplementary Online Content

Jones A, Warner-Schmidt J, Kwak H, et al. Efficacy and safety of the neuroplastogen TSND-201 for the treatment of PTSD: a randomized clinical trial. *JAMA Psychiatry*. Published online February 18, 2026. doi:10.1001/jamapsychiatry.2025.4625

eAppendix. Part A of IMPACT-1 (Open-Label) Overview and Results

eTable 1. Demographics and Baseline Characteristics (IMPACT-1, Part A)

eFigure 1. CAPS-5 Change from Baseline Over Time (IMPACT-1, Part A)

eFigure 2. Response and Remission on CAPS-5 at Day 64 (IMPACT-1, Part A)

eTable 2. Treatment-Emergent AEs Occurring in >1 Participant (IMPACT-1, Part A)

This supplementary material has been provided by the authors to give readers additional information about their work.

© 2026 Jones A et al. *JAMA Psychiatry*.

Supplemental Material

eAppendix. Part A of IMPACT-1 (Open-label)

Part A of IMPACT-1 was an open-label study evaluating the initial feasibility of TSND-201 as a treatment for PTSD. Eligible participants were adults (18 to 65 years) meeting the DSM-5 criteria for PTSD and of moderate or greater severity (CAPS-5 $\geq$ 35 at Screening and $\geq$ 28 at Baseline) and had previously tried a PTSD treatment, either psychotherapy or pharmacotherapy. Participants received 4 oral administrations of TSND-201, each separated by one week. Each dose was given as an initial dose of 150 mg followed by a second dose of 100 mg 90 minutes later. After the 4-week treatment period, participants were followed for an additional 6 weeks, for a total of 64 days from the first dose. CAPS-5 ratings were conducted 2 days after each dose (Day 3, 10, 17, and 24), then on Day 29, 36, 43, and 64.

The study was conducted at 3 sites in the UK between June 2023 and November 2023, a total of 14 participants were enrolled. Participants were predominantly White (93%) and female (71.4%). At baseline, the mean CAPS-5 score was 47.8, representing a severe PTSD population (**Table S1**).

eTable 1: Demographics and Baseline Characteristics (IMPACT-1, Part A)

Characteristic	TSND-201 (N=14)
Age, years (mean [range])	42.4 (23 - 65)
Sex, F (n [%])	10 (71.4%)
Race (n, %)	
White	13 (92.9%)
Not Reported	1 (7.1%)
CAPS-5 total score (mean [range])	47.8 (38 - 59)

Efficacy Results

Treatment with TSND-201 resulted in a rapid and durable improvement in CAPS-5 scores (**Figure S1**). The effects of TSND-201 were rapid, on Day 3 (two days after the first dose), CAPS-5 scores decreased by 8.4 points. By Day 10 (2 days after the second dose) CAPS-5 scores had decreased by 23.3 points. These effects were durable and maintained through 6 weeks after the last dose. At the end of study (Week 10), the CAPS-5 scores had decreased by 36.2 points from baseline.

eFigure 1: CAPS-5 Change from Baseline Over Time (IMPACT-1, Part A)

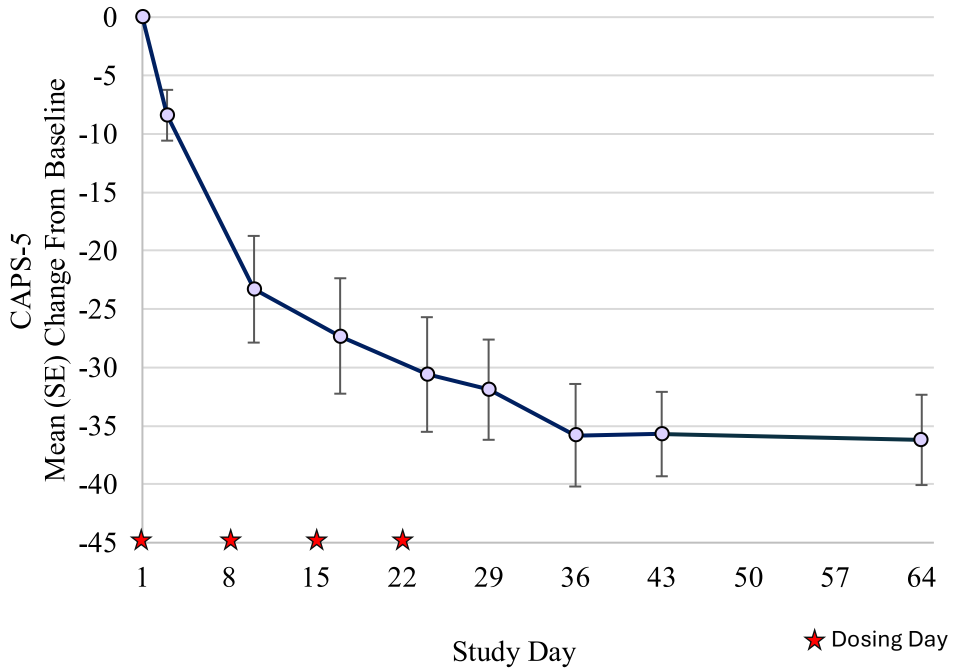

Treatment with TSND-201 resulted in high rates of response (≥ 50% improvement from baseline on CAPS-5) and remission (≤ 11 points on the CAPS-5 total severity score). TSND-201 rapidly induced response and remission, at Day 17 (two days after the 3rd dose), 46.2% of participants had a clinical response, and 38.5% were in remission. These effects were durable, at Day 64 (end of study) nearly 80% of participants had a response and over 60% achieved remission (**Figure S2**).

© 2026 Jones A et al. *JAMA Psychiatry*.

eFigure 2: Response and Remission on CAPS-5 at Day 64 (IMPACT-1, Part A)

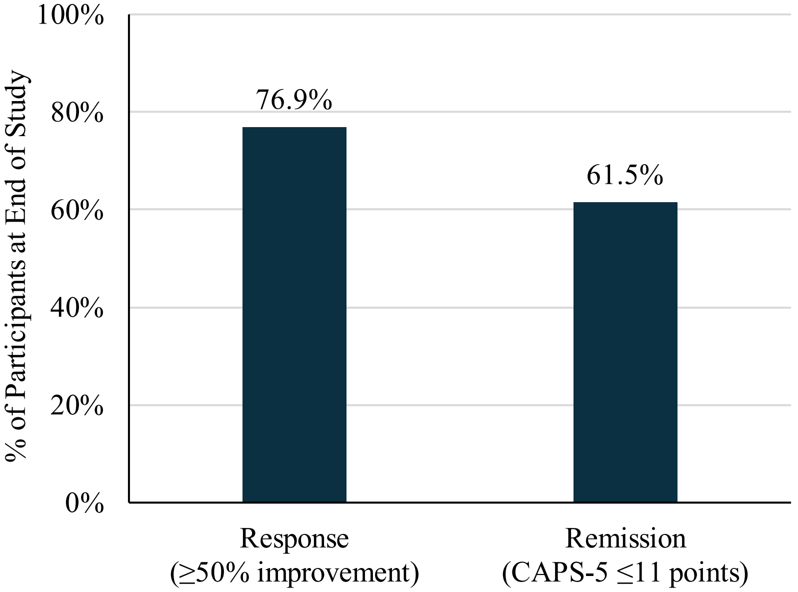

Safety

In Part A of the IMPACT-1 study, TSND-201 was well tolerated. The most commonly occurring treatment-emergent adverse events (TEAE; **Table S2**) were headache (43%), decreased appetite (28.6%), non-cardiac chest pain (21.4%), and fatigue (21.4%). No serious adverse events (SAE) occurred.

eTable 2: Treatment-Emergent AEs Occurring in > 1 Participant (IMPACT-1, Part A)

**Preferred Term**	**TSND-201 (150mg + 100mg) (N=14)** n (%)
At least one TEAE	11 (78.6)
Headache	6 (42.9)
Decreased appetite	4 (28.6)
Non-cardiac chest pain	3 (21.4)
Fatigue	3 (21.4)
Bruxism	2 (14.3)
Dizziness	2 (14.3)
Hyperhidrosis	2 (14.3)
Influenza-like illness	2 (14.3)
Insomnia	2 (14.3)
Nasopharyngitis	2 (14.3)

© 2026 Jones A et al. *JAMA Psychiatry*.

Conclusion

In this small feasibility study, TSND-201 demonstrated rapid and robust efficacy with a safety profile that was well tolerated. Based on the conclusion of the Part A results, the Sponsor and DSMB agreed to progress to Part B of IMPACT-1; a randomized, double-blind, placebo-controlled study appropriately powered to detect a treatment difference between TSND-201 and placebo.

© 2026 Jones A et al. *JAMA Psychiatry*.
