# Supplementary material for: Efficacy and Safety of the Neuroplastogen TSND-201 for the Treatment of PTSD: A Randomized Clinical Trial
Source: JAMA Psychiatry. 2026 Feb 18;83(5):469–77. doi: 10.1001/jamapsychiatry.2025.4625 (PMC12917749; doi:10.1001/jamapsychiatry.2025.4625)
Supplement: Supplement 4. — Data Sharing Statement. [file jamapsychiatry-e254625-s004.pdf]

Data Sharing Statement

Jones. Efficacy and Safety of the Neuroplastogen TSND-201 for the Treatment of PTSD.
JAMA Psychiatry. Published February 18, 2026. doi:10.1001/jamapsychiatry.2025.4625

Data
Additional Information: ClinicalTrials.gov Identifier: NCT05741710
Data available: No

Additional Information
Explanation for why data not available: The data are not publicly available due to proprietary restrictions from the sponsoring company.
